# Network pharmacology and bioinformatics were used to construct a prognostic model and immunoassay of core target genes in the combination of quercetin and kaempferol in the treatment of colorectal cancer

**DOI:** 10.7150/jca.85517

**Published:** 2023-07-03

**Authors:** Chenqiong Gu, LinDong Tang, Yinghui Hao, Shanshan Dong, Jian Shen, FangMei Xie, ZePing Han, WenFeng Luo, JinHua He, Li Yu

**Affiliations:** 1 Department of Biochemistry and Molecular Biology, School of Medicine, Jinan University, Guangzhou, 510632, Guangdong, P. R. China.; 2 Central Laboratory of Panyu Central Hospital, Guangzhou, 511400, Guangdong, P.R. China.

**Keywords:** quercetin, kaempferol, colorectal cancer, network pharmacology, Lasso-cox prognosis analysis, Prognostic model, Immune prediction.

## Abstract

**Purpose:** CRC is a malignant tumor seriously threatening human health. Quercetin and kaempferol are representative components of traditional Chinese medicine (TCM). Previous studies have shown that both quercetin and kaempferol have antitumor pharmacological effects, nevertheless, the underlying mechanism of action remains unclear. To explore the synergy and mechanism of quercetin and kaempferol in colorectal cancer.

**Methods:** In this study, network pharmacology, and bioinformatics are used to obtain the intersection of drug targets and disease genes. Training gene sets were acquired from the TCGA database, acquired prognostic-related genes by univariate Cox, multivariate Cox, and Lasso-Cox regression models, and validated in the GEO dataset. We also made predictions of the immune function of the samples and used molecular docking to map a model for binding two components to prognostic genes.

**Results:** Through Lasso-Cox regression analysis, we obtained three models of drug target genes. This model predicts the combined role of quercetin and kaempferol in the treatment and prognosis of CRC. Prognostic genes are correlated with immune checkpoints and immune infiltration and play an adjuvant role in the immunotherapy of CRC.

**Conclusion:** Core genes are regulated by quercetin and kaempferol to improve the patient's immune system and thus assist in the treatment of CRC.

## Introduction

CRC is a swart tumor extremely threatening to human health. In recent years, CRC has not only shown a trend of younger age but also has a lower survival rate for young patients 1. Due to the insidious early clinical symptoms, about 25% of patients have metastases at initial diagnosis 2. Metastasis also occurs in 50% to 60% of patients after treatment, so a radical surgical resection of CRC is becoming increasingly difficult 3. Therefore, the treatment of CRC has become a major challenge for the medical community. CRC treatment includes surgery, chemotherapy, immunotherapy, and targeted therapy. Nevertheless, the prognosis of CRC patients remains suboptimal due to the limitations of surgery, radiation dose limitation, toxic side effects of chemotherapy, and drug resistance 4. At present, targeted therapy, immunotherapy, and other therapies have made significant progress in CRC treatment, but some adverse reactions will also occur during the treatment process, which will seriously reduce the patient's quality of life 5. Immune checkpoint inhibitors (ICI) for immunotherapy, and ICI activation can cause some toxic side effects, which is a momentous fling down the gauntlet in ICI clinical application 6. Currently, the therapeutic goal of CRC is to prolong overall survival and improve patient quality of life.

Some studies have shown that the curative effect of traditional Chinese medicine on liver cancer 7, breast cancer 8, and so on seems to be very promising. Therefore, it is particularly important to clarify the anticancer mechanism of traditional Chinese medicine treatment. In clinical studies, quercetin is used in the treatment of diabetes 9-13, hyperlipidemia 14, and nonalcoholic fatty liver disease (NAFLD) 15-16. Some preclinical studies have found that quercetin has anticancer effects by inhibiting cell proliferation and metastasis, inducing apoptosis and autophagy 17-20, and can also induce apoptosis in HCT116 and HT29 cells 21-23. However, there are few relevant clinical studies on quercetin and antitumor. In addition, it has been reported that kaempferol can also reduce the risk of skin and liver cancer 24 and inhibits the growth of CRC 25 by accelerating the necrosis and apoptosis of cells. Quercetin and kaempferol not only play an anti-tumor effect alone but also inhibit the proliferation of colorectal cancer cells when used together 26. The above studies show that kaempferol and quercetin are potential drugs in CRC treatment.

In the development of TCM, researchers have achieved good preliminary results in revealing the comprehensive effect of multi-channel, multi-target, and multi-component of TCM by referring to the research ideas of network pharmacology 27-29. However, the pharmacological mechanism of Chinese herbal medicine (CHM) has not been fully elucidated, so the establishment of the CHM database is especially important for network pharmacology research 30 which can be used for mutual verification of network analysis and experiments. Therefore, network pharmacology is being used as a very useful tool to understand drug-target interactions. The purpose of this research was to explore the mechanism of action of quercetin and kaempferol in CRC treatment. The targeting network of quercetin and kaempferol for the treatment of CRC was constructed by the method of network pharmacology, the core genes were screened, and the drug target gene network composed of fifty-seven target genes was obtained. Based on our findings, we can all the better understand the reciprocity between these two drugs and supply theoretical backing for future basic study. Then we analyzed the genes differentially expressed between CRC and para-cancerous tissues to so much better predict the survival time of CRC patients treated with quercetin and kaempferol and to provide a reference for clinical drug use. Univariate Cox together with multivariate Cox regression was used to analyze 49 Differentially expressed genes (DEG) in the drug targeting network and obtain three key prognostic genes. These three prognostic genes were analyzed by the lasso-Cox regression analysis algorithm, and the nomogram predictive map was set up to boost clinical guidance. In addition, the function of genes in this model in tumor occurrence and development and immunologic response was studied by gene cluster enrichment analysis (GSEA).

Finally, to study the immune-related effect of quercetin and kaempferol, we predicted a correlation between the expression of the core genes and immune checkpoint genes and further discussed the effects of quercetin and kaempferol on the immunologic response.

## Materials and Methods

### Data collection

Based on the PubChem database and TCMSP database, quercetin and kaempferol target genes were obtained. And from the Gene Cards database, Online Mendelian Inheritance in Man (OMIM), Pharmacogenetics and Pharmacogenomics Knowledge Base (PharmGkb), Database of gene-disease associations (DisGeNET), and Treatment Target Database (TTD) were used to obtain CRC disease genes. The training gene set was obtained from TCGA and validation gene sets were obtained from the GEO database. Immunohistochemical images of gene expression in para-cancerous tissues and tumors were obtained from the Human Protein Atlas Database (HPA).

### Construction and screening of drug-target interaction network

The targets of quercetin and kaempferol were merged as drug targets, and the acquired genes in five databases were merged as pathogenic gene sets to obtain the two crossover gene sets. Protein-protein interaction (PPI) networks were constructed through the STRING database, and outlier genes were removed. The PPI network was imported into Cytoscape 3.9.1, and the network core genes were screened using the CytoNCA 31 plugin. The screening criteria are betweenness centrality (BC), degree centrality (DC), closeness centrality (CC), eigenvector centrality (EC), local average connectivity (LAC), and network centrality (NC). The filter conditions are all greater than the median value.

### Functional and functional enrichment analysis of the drug-target network

To explore the significant functions and cellular pathways of the core genes. The Kyoto Encyclopedia of Genes and Genomes (KEGG) and Gene Ontology (GO) enrichment analysis were performed using the R package “cluster Profiler” 32. The pathways were significantly enriched when a *P* value of < 0.05 and an FDR of < 0.25 were considered.

### Core gene analysis

The RNA-seq data in the TCGA database were grouped into tumor groups and para-cancerous groups, heat maps and boxplots of core gene expression levels were plotted, and correlation maps of gene expression were plotted. Undifferentiable genes were removed to obtain the differentially expressed genes (DEGs).

### Construction of the prediction model of Lasso-cox regression

Univariate and multivariate Cox regression analyses were used to determine the prognostic value of DEGs (*P* < 0.05). In this research, we used the R package glmnet 33, using the lasso-cox method to identify CRC prognostic genes. We calculated the optimal cutoff for RiskScore using the R package maxstat 34, based on which we differentiated patients into high and low groups and generated Kaplan-Meier survival curves to evaluate the predictive performance of the associated risk genes.

### Validation of the Lasso-Cox regression prediction model

The CRC transcriptome and clinical data of two gene chips in GEO were used to verify the model. We use R-packet pROC 35 for ROC analysis to verify the performance of the model prediction. The risk curve, risk and survival scatter plots, and heat maps of the validation model gene expression are plotted. Forest plots were drawn based on univariate Cox regression analysis. We used the R software package “rms” 36 and the Cox method to establish a nomogram to evaluate the prognostic significance of some features in the sample and predict the survival of patients.

### Correlation between prognostic model and tumor immunity

The data on immune cell level were downloaded from TIMER2.0 37 online immune database, and the bar chart and correlation chart of immune cell content were drawn. To explore the pertinence between the gene expression and immune cells in Lasso regression prognostic model, we used the online analysis tool SangerBox 38 to draw the pertinence map between 3 prognostic genes expression and immune cells, and the pertinence map between 3 prognostic genes expression and immune checkpoint. We selected ESTIMATE 39 in R package IOBR 40 to score the immune infiltrating cells of CRC samples in TCGA. And draw the Kaplan-Meier survival curve of the relationship between the content of stromal cells and immune cells and the survival of patients.

## Results

### Sample collection

According to inclusion criteria 41: (a) complete gene expression information (b) complete survival information, the exclusion standard was as follows: (a) incomplete gene expression (b) no survival time and survival status in the clinical information. Transcriptome data and 627 clinical data from 625 CRC organizations and 51 para-cancerous tissues were acquired from the TCGA database, including overall survival time, survival status, age, gender, and other data (Table S1). A sum of 728 transcriptome and clinical information (Table S2) were obtained from GEO's two gene chips, GSE103479 42 and GSE39582 43.

### Construction of the drug-target interaction network

**Figure 1:**
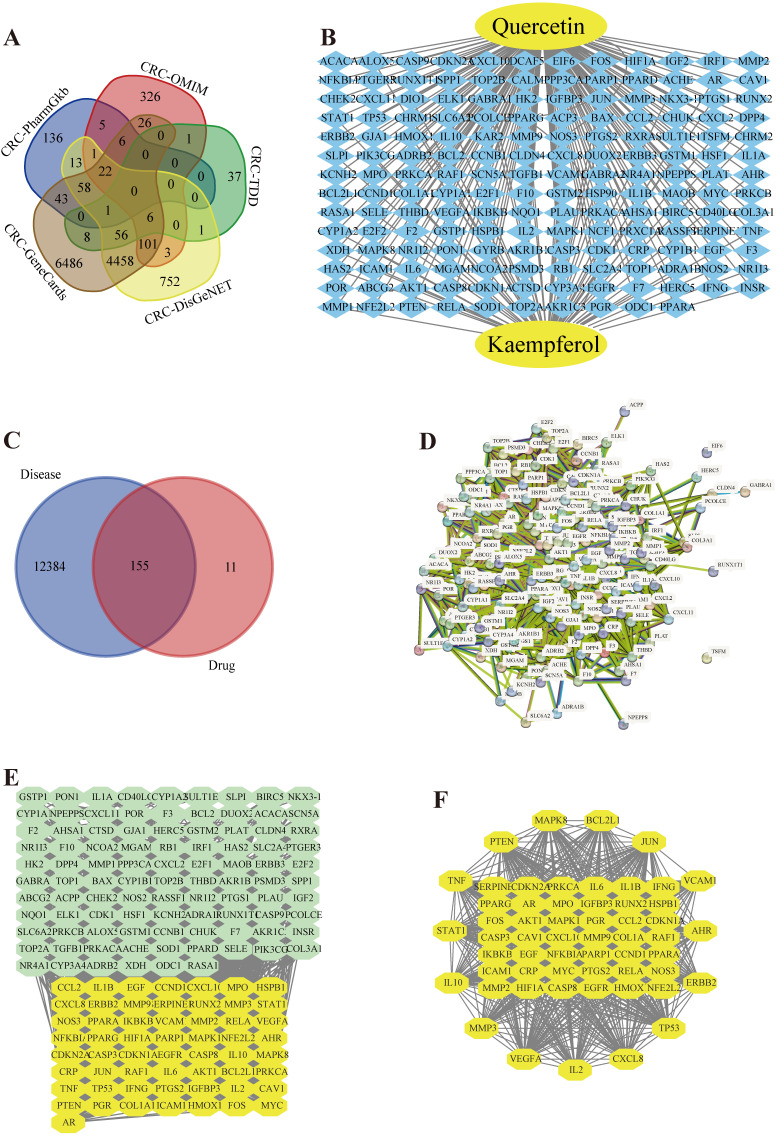
** (A)** A Venn diagram of the five disease-gene databases. **(B)** Drug target interaction network. **(C)** Venn chart of the overlapping of drug targets and CRC genes. **(D)** Protein-protein interplay. **(E, F)** Screening of the key genes in the network.

In this research, we used the study notion of network pharmacology, given the possible loss of genes in individual disease databases, 12549 CRC causal genes were obtained from 5 databases (Figure 1 (A)). 166 quercetin targets and 64 kaempferol targets were obtained from the PubChem database and TCMSP database, and 166 drug targets were removed (Figure 1 (B)). The target genes of quercetin and kaempferol have 155 overlapping genes of CRC (Figure 1 (C)), which are the targets of quercetin and kaempferol for the treatment of CRC. A PPI network containing 154 target proteins (Figure 1 (D)) was obtained through the STRING online database, which was imported into Cytoscape 3.9.1 to obtain the interaction network map between the drug and target genes (Figure 1 (E)). Using plug-in CytoNCA to filter overlapping memes, 57 core memes were obtained (Table S3) (Figure 1 (F)): AKT1, TP53, TNF, IL6, VEGFA, JUN, IL1B, CASP3, PTGS2, HIF1A, MYC, EGFR, EGF, MMP9, CXCL8, CCND1, PTEN, PPARG, CCL2, FOS, IL10, MMP2, ICAM1, NFKBIA, ERBB2, HMOX1, VCAM1, CASP8, BCL2L1, RELA, IFNG, SERPINE1, CDKN2A, STAT1, MAPK8, IL2, NOS3, MAPK1, CDKN1A, IKBKB, PPARA, MMP3, AR, CRP, CXCL10, CAV1, NFE2L2, MPO, PGR, PARP1, IGFBP3, HSPB1, PRKCA, RUNX2, COL1A1, AHR, and RAF1.

### GO and KEGG pathway enrichment analysis

**Figure 2:**
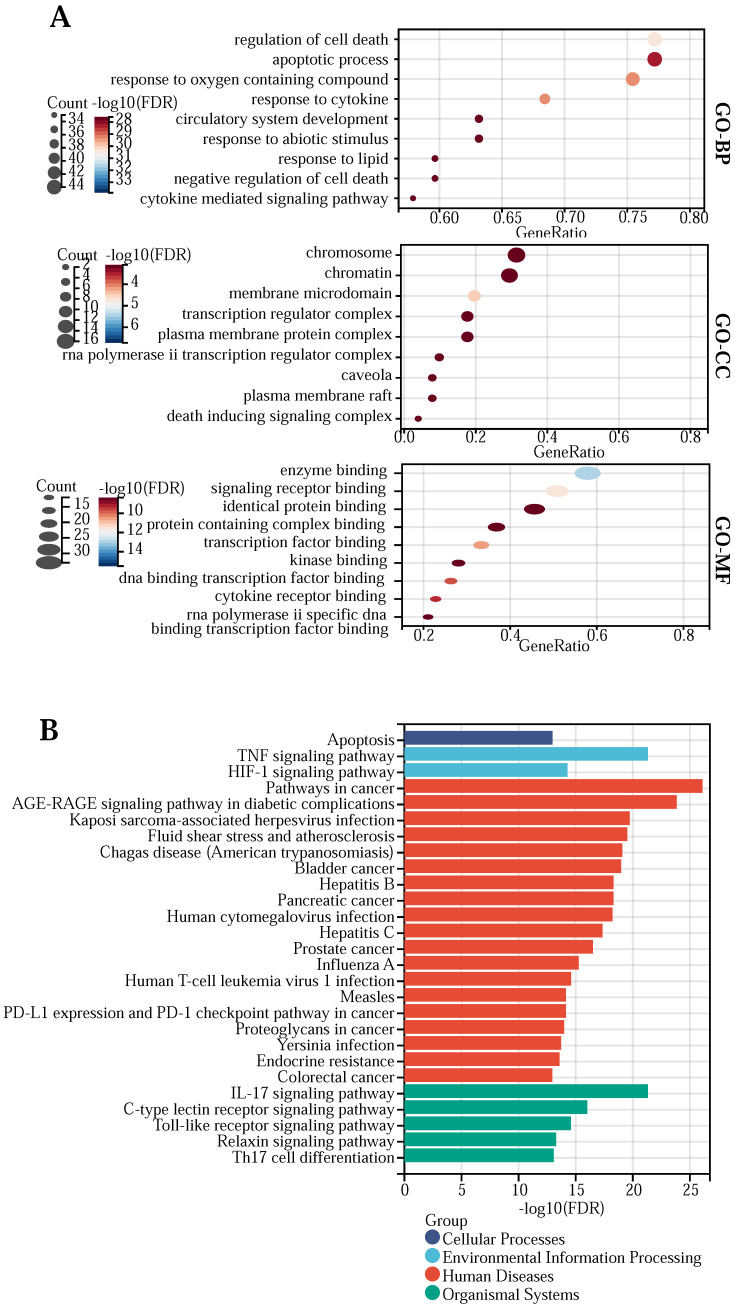
**(A)** GO bubble plot of the network key genes. **(B)** Bar graph of the network core gene KEGG pathway.

To elucidate the functions and potential actions of drugs versus core genes, we performed GO and KEGG enrichment analyses (Table S4). The GO enrichment results showed that quercetin and kaempferol were enriched in the functions of “regulation of cell death”, “response to oxygen-containing compound”, “response to cytokine”, “apoptotic process”, “response to lipid”, “cytokine-mediated signaling pathway”, “circulatory system development”, “negative regulation of cell death”, “response to abiotic stimulus”, “regulation of cell differentiation”, “membrane microdomain”, “transcription regulator complex”, “chromatin”, “caveola”, “chromosome”, “rna polymerase ii transcription regulator complex”, “death-inducing signaling complex”, “plasma membrane raft”, “plasma membrane protein complex”, “enzyme binding”, “signaling receptor binding”, “transcription factor binding”, “dna binding transcription factor binding”, “cytokine receptor binding”, “rna polymerase ii specific dna binding transcription factor binding”, “identical protein binding”, “protein-containing complex binding”, “kinase binding” and so on. (Figure 2 (A)). KEGG enrichment results show that the main signal pathways of 57 core genes are located at “Pathways in cancer”, “AGE-RAGE signaling pathway in diabetic complications”, “TNF signaling pathway”, “IL-17 signaling pathway”, “Kaposi sarcoma-associated herpesvirus infection”, “Fluid shear stress and atherosclerosis”, “Chagas disease (American trypanosomiasis)”, “Bladder cancer”, “Hepatitis B”, “Pancreatic cancer”, “Human cytomegalovirus infection”, “Hepatitis C”, “Prostate cancer”, “C-type lectin receptor signaling pathway”, “Influenza A”, “Human T-cell leukemia virus 1 infection”, “Toll-like receptor signaling pathway”, “HIF-1 signaling pathway”, “Measles”, “PD-L1 expression and PD-1 checkpoint pathway in cancer”, “Proteoglycans in cancer”, “Yersinia infection”, “Endocrine resistance”, “Relaxin signaling pathway”, “Th17 cell differentiation”, “Apoptosis” and “Colorectal cancer” et al.(Figure 2(B)).

### Construction and validation of the Lasso regression prediction model

**Figure 3:**
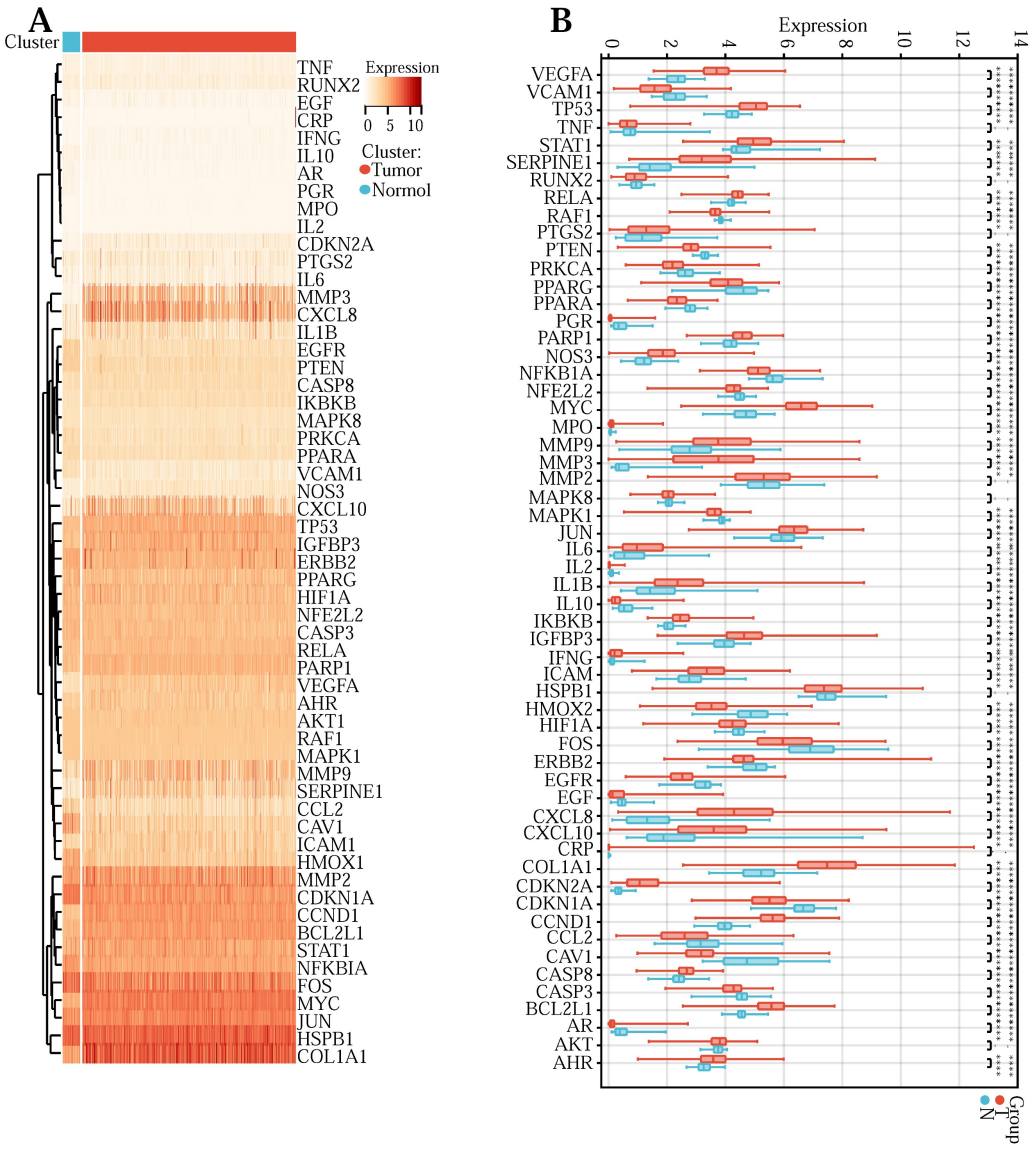

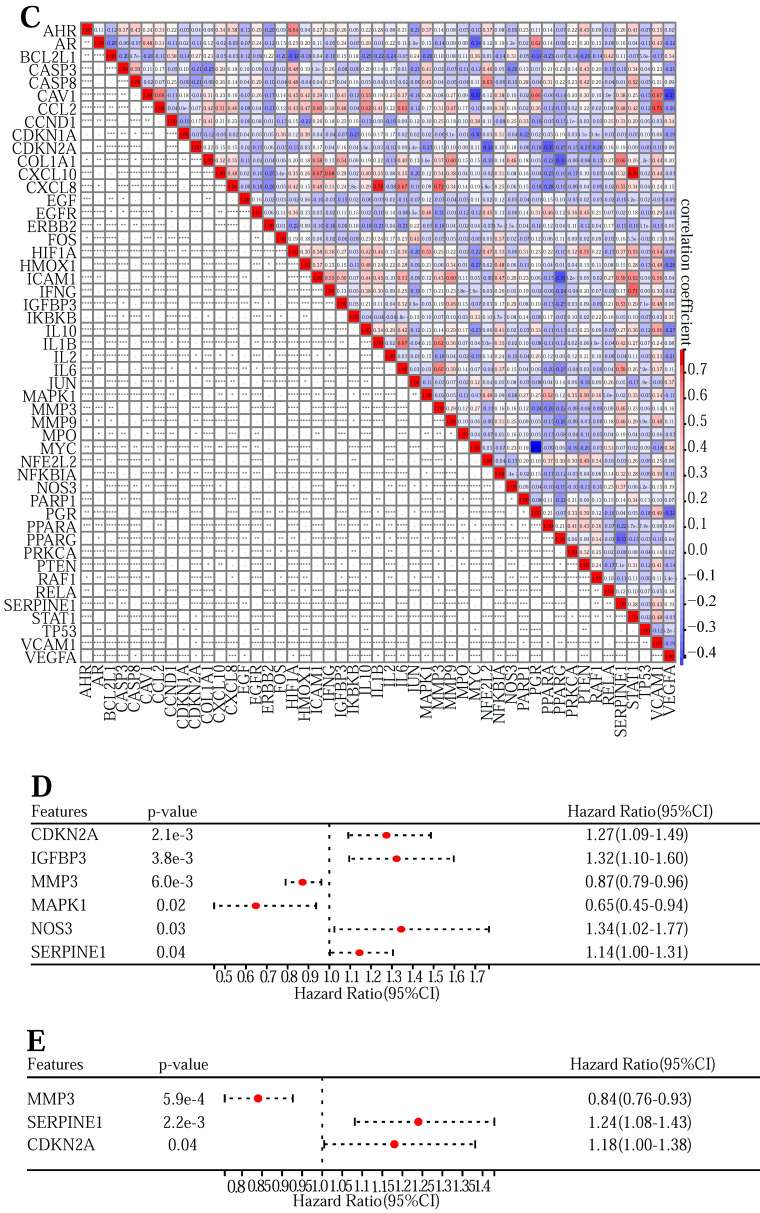

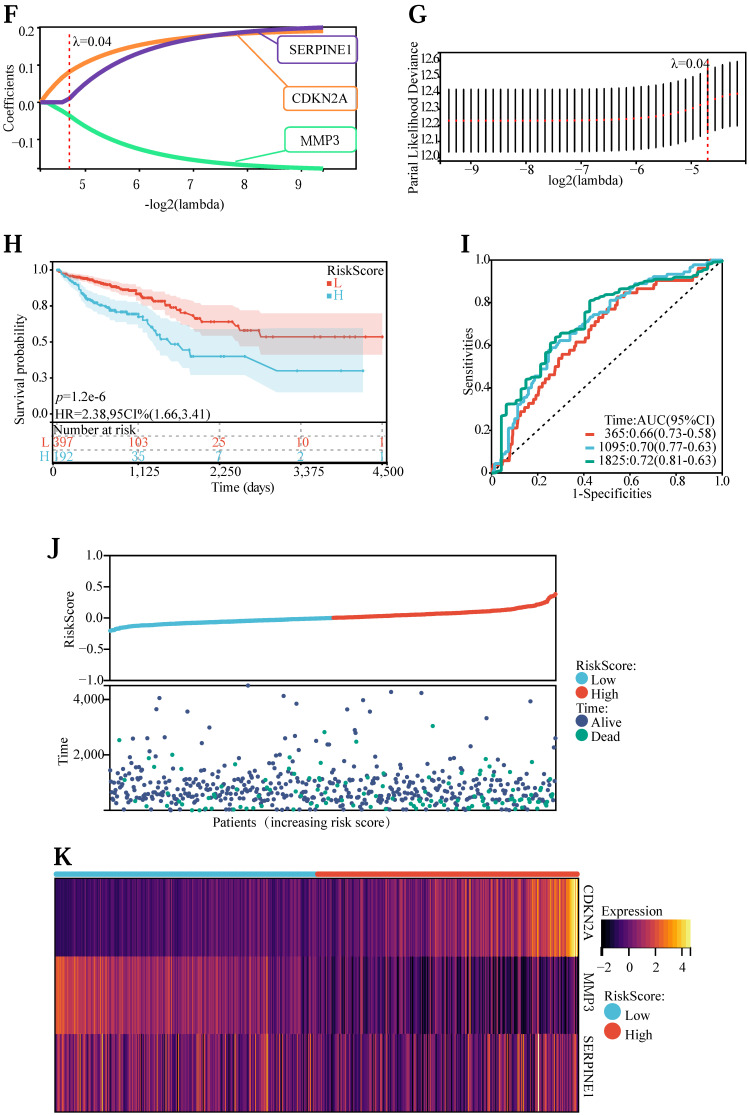
**(A)** Heatmap of network core gene expression in the TCGA database. **(B)** Boxplot of differential expression of network core genes.** (C)** Correlation plot of core differential gene expression. **(D)** Unigenic Cox prognostic regression analysis of core differential genes. **(E)** Multigenic Cox prognostic regression analysis of core differential genes. **(F, G)** Lasso prognostic regression model. **(H)** Survival curves in the TCGA database.** (I)** ROC curve to verify the accuracy of risk. **(J)** Risk curve, risk, and survival scatter plot. **(K)** Heatmap of model gene expression in the high- and low-risk groups.

First of all, to explore the expression levels of the above 57 core genes in normal tissues and tumor samples, we drew a heat map (Figure 3 (A)) and a violin map (Figure 3 (B)). Among them, TNF, RUNX2, PTGS2, MMP2, MAPK8, HSPB1, AKT1, and CRP showed no difference in expression between normal and tumor tissues. Genes with no difference were removed, and 49 DEGs were obtained. Next, to explore the expression correlation of these DEGs, we draw a co-expression association map of genes (Figure 3 (C)). There were 5 groups with a co-expression correlation coefficient greater than 0.7: STAT1 and CXCL10 (correlation coefficient: 0.79), CXCL8 and IL1B (correlation coefficient: 0.78), CCL2 and VCAM1 (correlation coefficient: 0.75), CXCL8 and MMP3 (correlation coefficient: 0.72), IFNG and STAT1 (correlation coefficient: 0.71). Then, we executed univariate Cox regression analysis (Figure 3 (D)) and multivariate Cox prognosis (D) (Figure 3 (E)), and we adopted the R package glmnet, integrating survival time, survival status, and gene expression data for prognostic regression analysis using the lasso-cox method (Figure 3 (F) and Figure 3 (G)). When λ = 0.0383989011310987, 3 prognostic model genes: CDKN2A, MMP3, and SERPINE1 were obtained. The regression coefficients were Coef (CDKN2A) = 0.082101134622246, Coef (MMP3) = -0.0355844623404235, Coef (SERPINE1) = 0.0100737687120257 (Table S5).

The risk score was counted according to the risk score formulary: RiskScore=0.082101134622246 * CDKN2A-0.0355844623404235 * MMP 3 +, 0.0100737687120257 * SERPINE1. The topgallant cutoff value was counted using the R package maxstat as 0.0487909863090454. Based on this, dividing patients into high and low groups (Figure 3 (H)), the risk score can differentiate the survival time and survival status of the high-risk and low-risk populations. The discrepancy in prognosis in both the high and low-risk groups was evaluated by log-rank test, and the prognosis showed a marked difference between the two groups (*P* <0.001), and patients in the high-risk group may have shorter survival and worse survival status than those in the low-risk group. We drew ROC curves (Figure 3 (I)) to validate model prediction exactitude and dependability. The AUC value is >0.6, which proves that the Lasso-Cox prognostic regression curve is credible. Based on this, we drew the risk curve, hazard, survival scatter diagram (Figure 3 (J)), and the expression heat map of prognostic genes (Figure 3 (K)) and analyzed the nexus between diverse risk scores and patient follow up time, survival status and gene expression. With the rise of risk score, the survival ratio of patients reduced significantly (3J), which indicated that the MMP 3 gene was a protective factor, downregulated with the rise of risk score, CDKN2A, and ERPINE1 genes are risk factors and were upregulated with the rise of risk score. In conclusion, we found that the death probability and gene expression level of high-risk patients were higher than those in the low-risk group.

**Figure 4:**
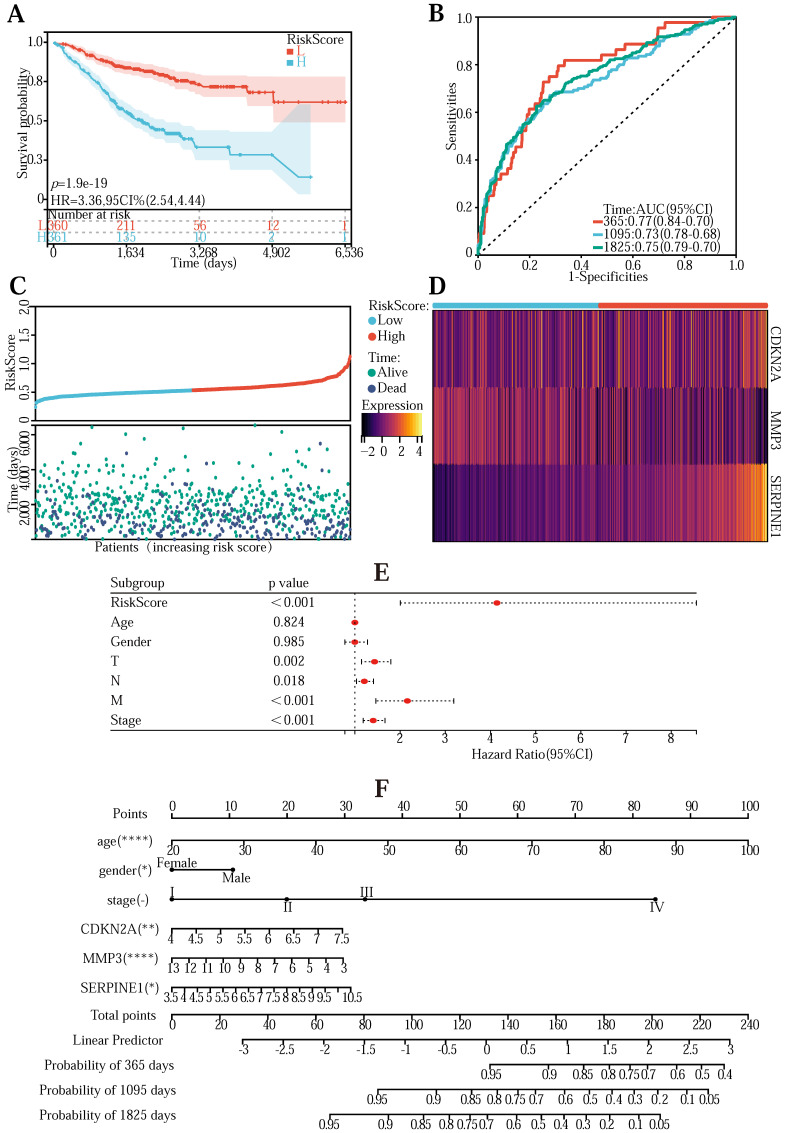

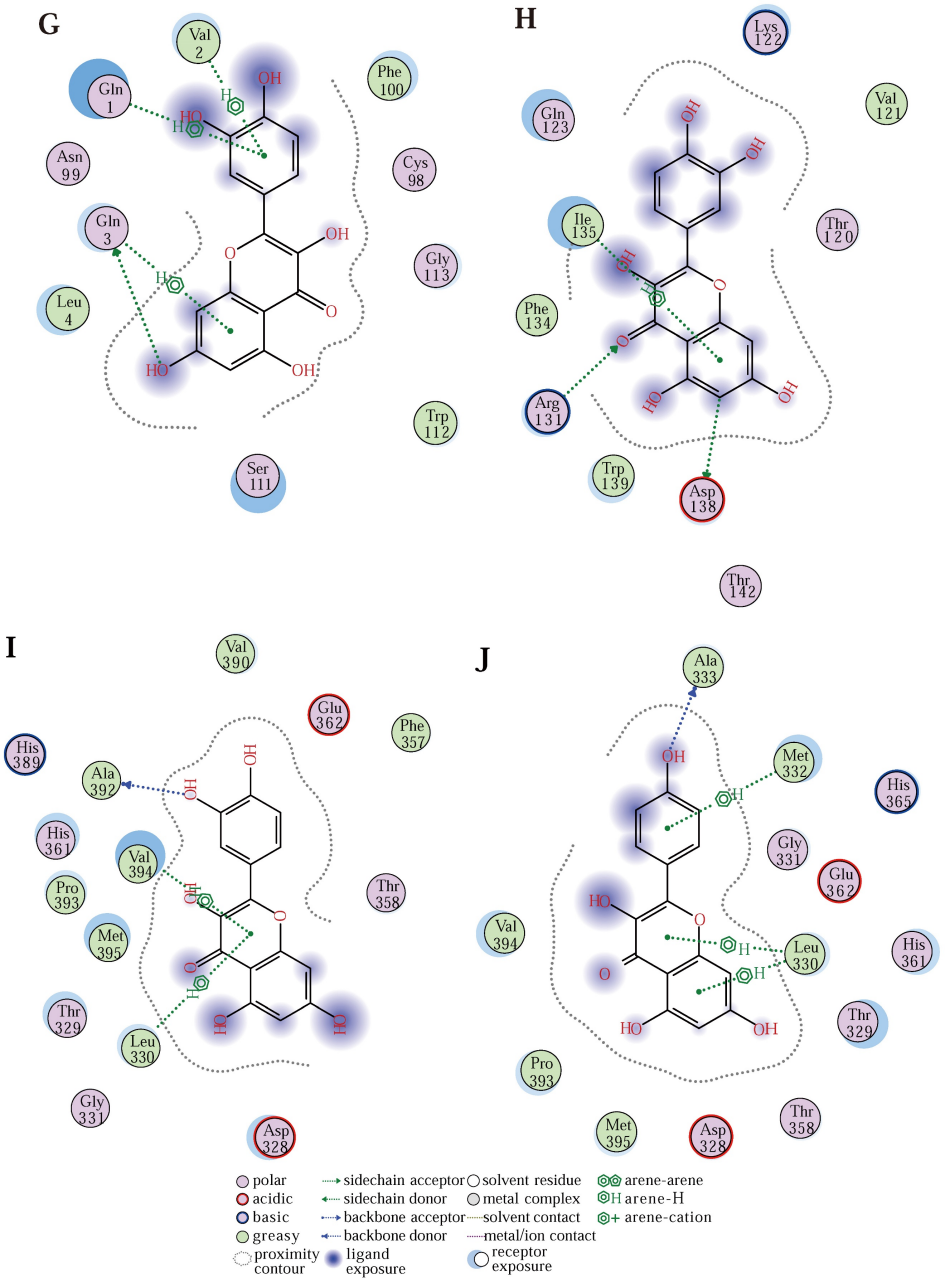
**(A)** Survivorship curves in the GEO database. **(B)** ROC curve to validate the accuracy of risk. **(C)** Risk curve, risk, and survival scatter plot. **(D)** Heatmap of model gene expression in the high- and low-risk groups. **(E)** Univariate prognostic regression test. **(F)** Used to forecast survival in patients with the nomogram. **(G)**The predicted binding model of quercetin and CDKN2A **(G)**, SERPINE1 **(H),** and MMP3 **(I)** proteins, and the predicted binding model of kaempferol to MMP3 **(J)** proteins.

To validate the universality of the Lasso-Cox prediction regression model, we verified the model using the GeneChip from the GEO database (Table S6). The transcriptomic information in the chip was first normalized. Combine gene expression and clinical information to draw survival curves into high-risk and low-risk groups and explore the survival of high/low-risk groups in the validation data set (Figure 4 (A)) (*P* <0.001). The ROC curve is between (0.73-0.0.77) AUC, indicating that the pattern is relatively accurate (Figure 4 (B)). The risk curve, hazard, and survival scatter diagram (Figure 4 (C)), and the model gene expression heatmap (Figure 4 (D)) were significantly different in both the high-risk and low-risk groups. These results demonstrate the universality and accuracy of the Lasso-Cox predictive regression model.

We next performed a one-factor outcome analysis (Figure 4 (E)) and found that risk values were highly interrelated with specimen survival status in extensive clinical data. Combining the risk score formulary and survival period to draw the survival prognosis nomogram (Figure 4 (F)) can predict the survival of patients at 1-5 years.

Finally, to explore whether quercetin and kaempferol interact and function with the three key genes, we used Molecular Operating Environment (MOE) 2019 software to draw the binding model of CDKN2A^44^ (Figure 4 (G)), SERPINE1^45^ (Figure 4 (H)) and MMP3^46^ (Figure 4 (I)) to quercetin, and the binding profile of MMP3 (Figure 4 (J)) to kaempferol, demonstrated that quercetin and kaempferol binding to target proteins may produce pharmacological effects.

### The GSEA cellular pathway enrichment analysis of the model genes

**Figure 5:**
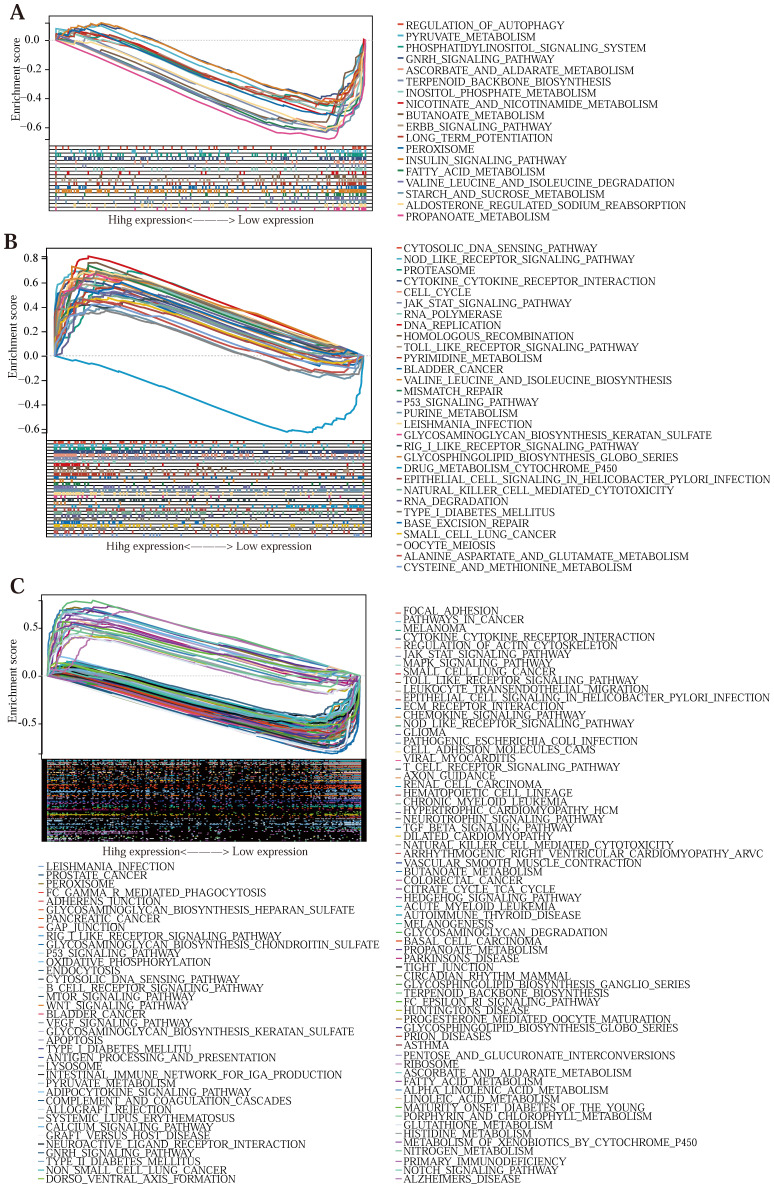

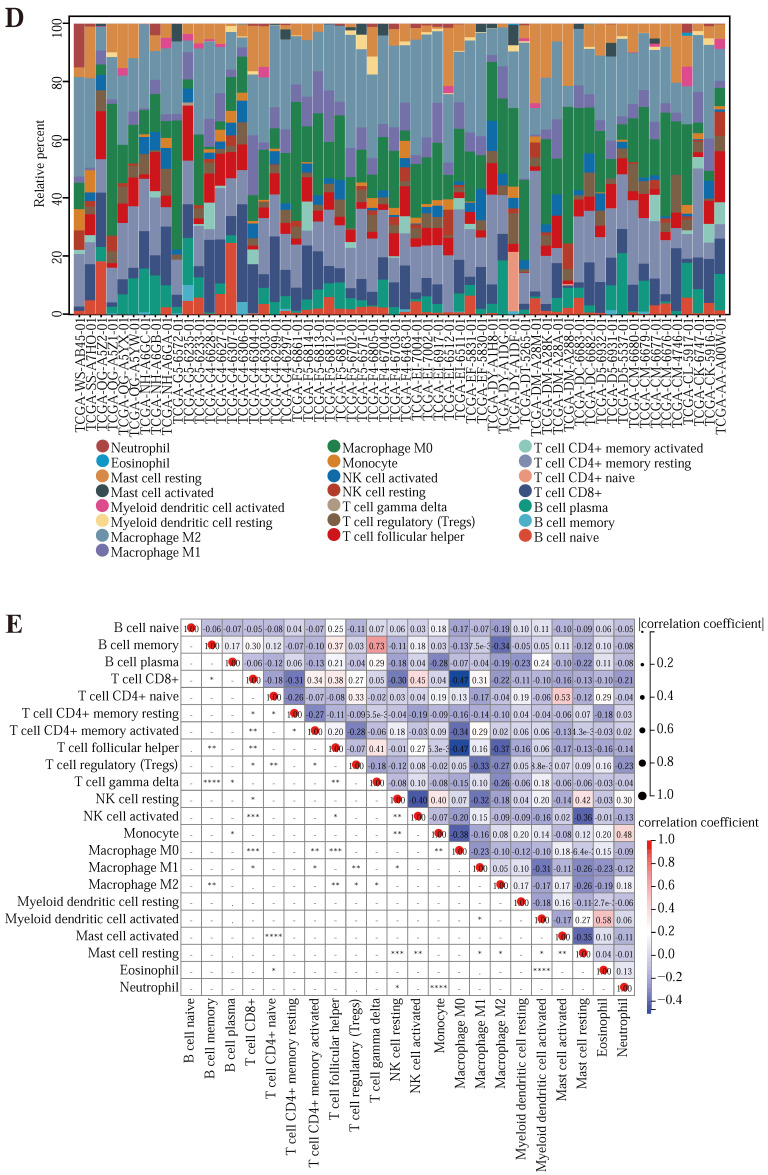

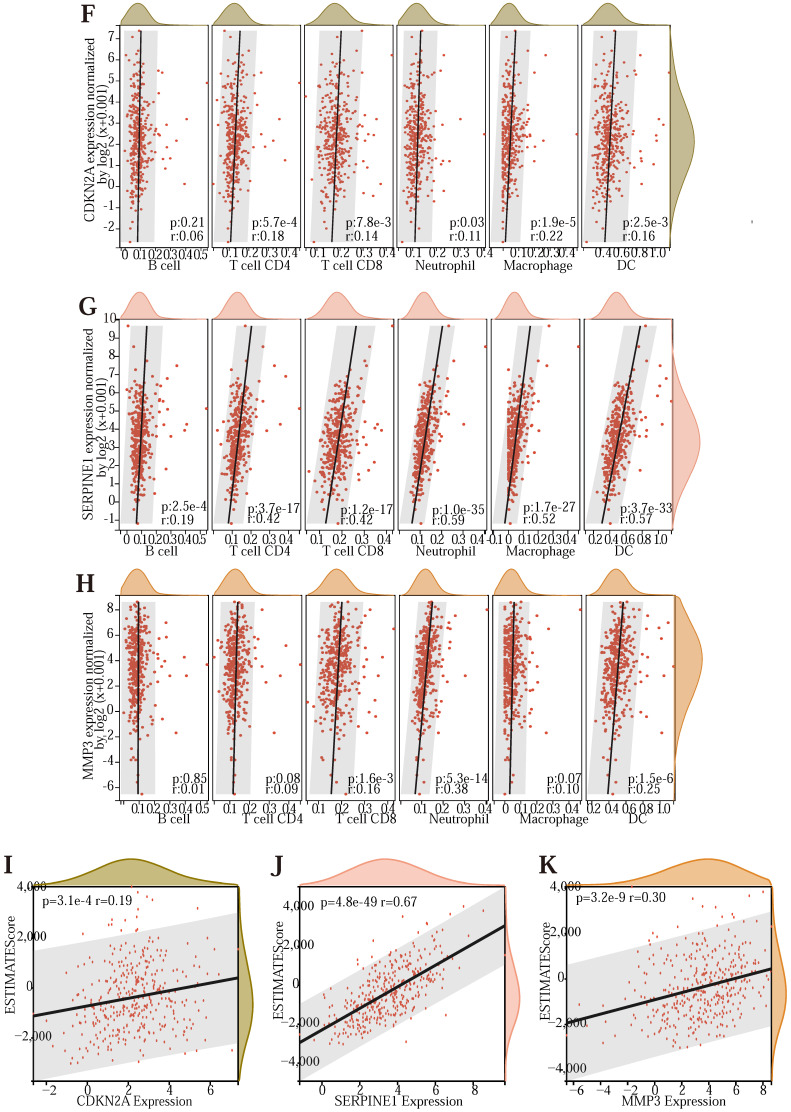

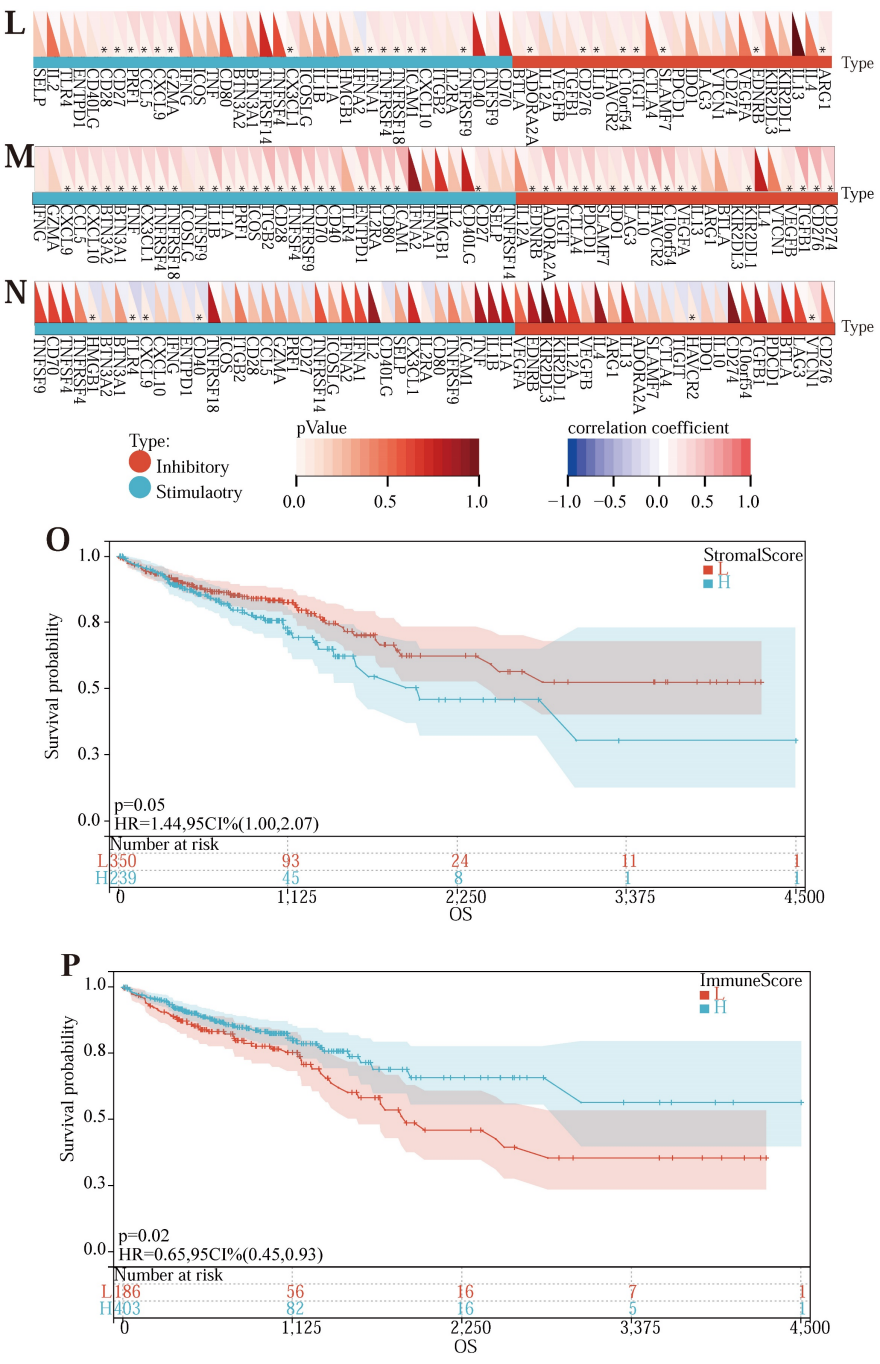
** (A, B, C)** GSEA enrichment analysis of CDKN2A, MMP3, and SERPINE1, respectively. **(D)** Histogram of the multifarious immune cell contents in the TCGA database. **(E)** Graph of correlations between the levels of multifarious immune cells. **(F, G, H)** A linear regression graph of various immune cell levels and prognostic gene expression.** (I, J, K)** Linear regression plots of immune infiltration scores and prognostic gene expression relationships in different samples.** (L, M, N)** The map of the linear regression between expression levels of multifarious immune checkpoints and prognostic gene expression. **(O, P)** Survival plots of stromal cells versus immune cells in the samples.

To investigate the association of tumor-related and immune-related pathway genes in the Lasso-Cox prognostic regression model, we performed a GSEA 47 pathway-related enrichment analysis for prognostic genes (Table S7) (The pathways were regarded to be significantly enriched when a *P* value of < 0.05 and an FDR of < 0.25). The GSEA pathway enrichment results of SERPINE1 (Figure 5 (C)) genes were the most, with 103 significantly related pathways, among which 30 signal pathways were related to tumor development and 18 immune-related signal pathways; MMP3 (Figure 5 (B)) has 33 related pathways, and 7 with both tumor-related and immune-related; CDKN2A (Figure 5 (A)) showed 24 enrichment results, 2 related to tumor, and 1 related to immune. This shows that the three prognostic genes screened above are closely related to tumorigenesis and development and immunotherapy.

### Relationship of model genes to tumor immunity

To further explore whether the genes in the model affected the immune response. We downloaded the immune prediction data (Table S8) of CRC samples in TCGA from the TIMER2.0 database and drew the histogram of various immune cell content in some samples (Figure 5 (D)) and the immune cell correlation map (Figure 5 (E)). The relevancy plot shows that T cell CD8+ and Macrophage M0, T cell follicular helper, and Macrophage M0 had a minimal correlation of -0.47; The maximum correlation between B cell memory and T cell gamma delta was 0.73. Next, to explore the relevance between the three prognostic genes and immune cell expression, immune infiltration, and immune checkpoints (Table S9), we drew several scatter plots (Figure 5 (F) -5 (K)). As shown in the image, CDKN2A was significantly associated with T cell CD4, T cell CD8, Neutrophil, Macrophage, and Dendritic cell (*P* <0.05); SERPINE1 showed significant associations with B cell, T cell CD4, T cell CD8, Neutrophil, Macrophage and Dendritic cell* (P* <0.05); MMP3 were significantly associated with T cell CD8, Neutrophil and Dendritic cell (*P* <0.05). The expression of the prognostic genes CDKN2A, SERPINE1, and MMP3 was significantly and positively correlated with the immune infiltration score of the samples (*P* <0.001). These results indicate that three prognostic-related genes have a significant association with immune cells.

In addition, we calculated the Pearson correlation of CDKN2A, SERPINE1, and MMP3, and five categories of immune pathway markers, and prognostic gene expression was closely related to immune checkpoints (Figure 5 (L) -5 (N)). Thus, the prognostic genes of the Lasso-Cox prognostic regression model were associated with immune cells and significantly positively associated with the immune checkpoint. In addition, we also performed the survival analysis of the tumor microenvironment of the samples (Table S10) and drew the survival curve of the stromal cells and the immune cells in the samples (Figure 5 (O) -5 (P)).

The results implied that there was no relativity between stromal cell content score and the survival status of the patient, but there was a correlation between immune cell content score and patient survival status, indicating that the more the immune cell content in the tumor tissue, the longer the survival time, that is, the deeper the degree of immune infiltration, the longer the patient's survival time may be.

These results suggest that there is a high correlation between the above three prognostic genes and immune cells, immune infiltration, and immune checkpoints, which can be used as targets for drug development in the immune microenvironment.

### Immunohistochemical expression levels in the HPA database

**Figure 6:**
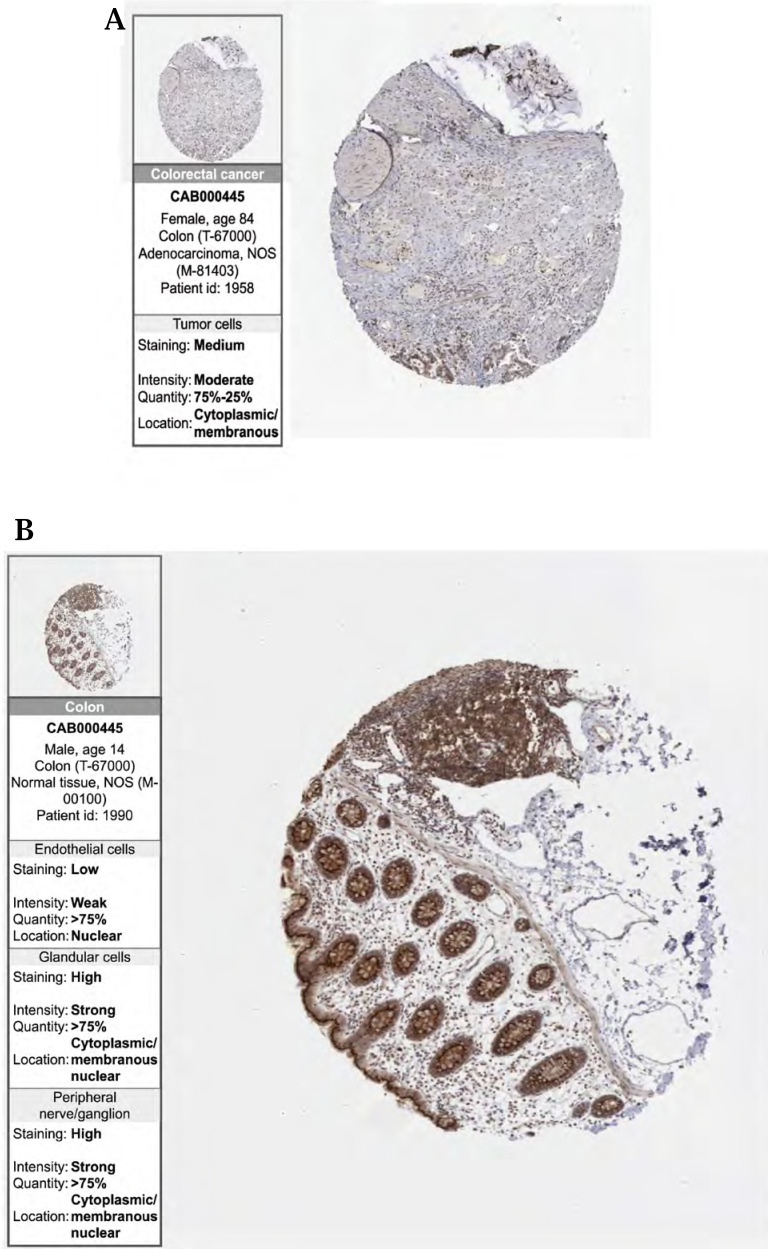

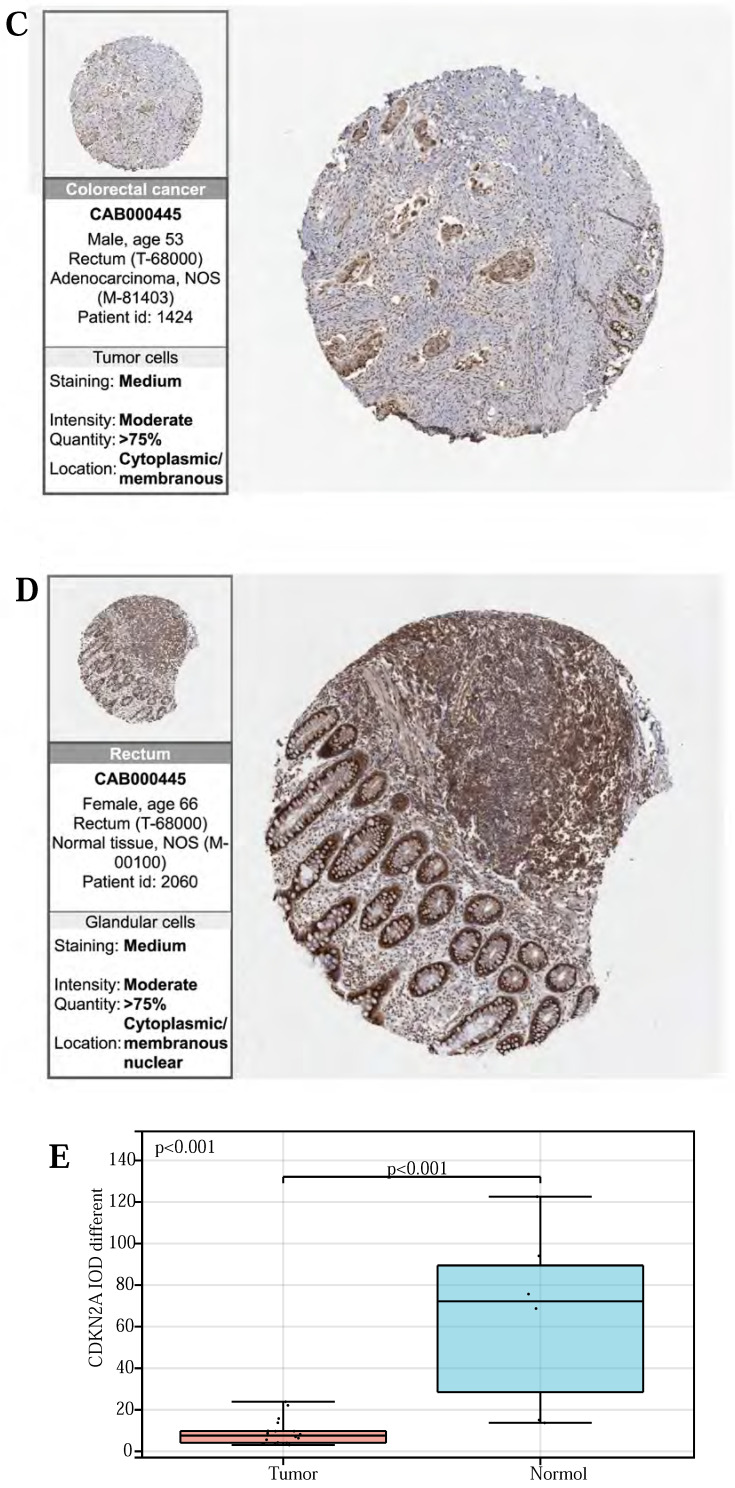

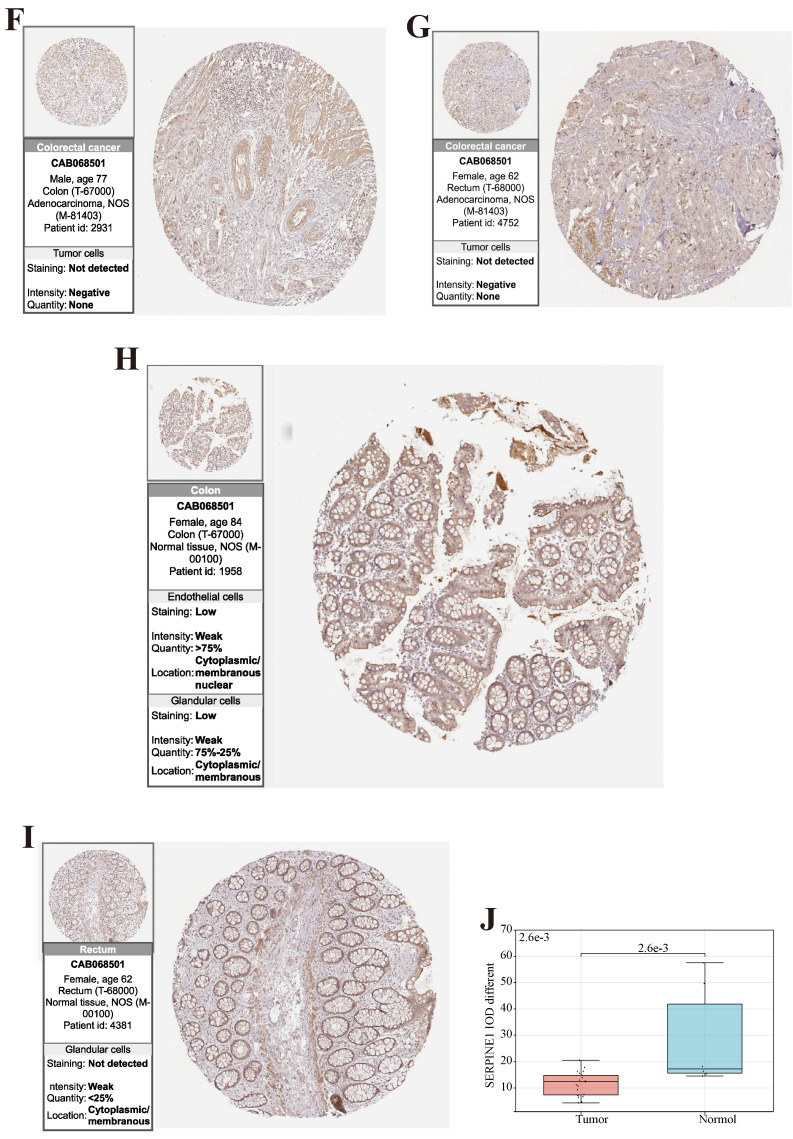

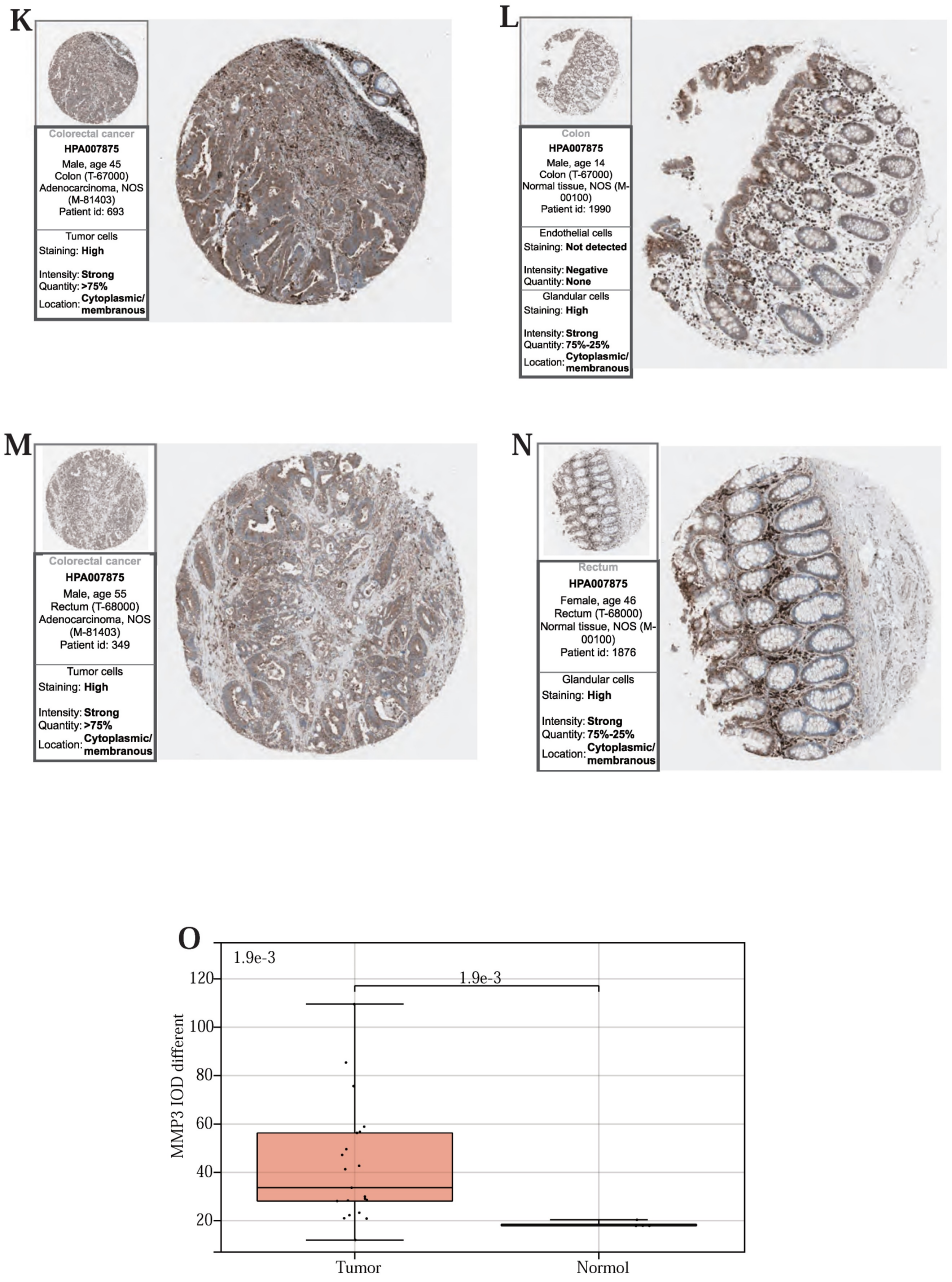
**(A, B, C, D)** Immunohistochemical figures of CDKN2A in normal and CRC tissues from the HPA database. **(F, G, H, I)** Immunohistochemical figures of SERPINE1 in normal and CRC tissues from the HPA database. **(K, L, M, N,)** Immunohistochemical figures of MMP3 in normal and CRC tissues from the HPA database. **(E, J, O)** Boxplots of IOD differences in CDKN2A, SERPINE1, and MMP 3 in normal and cancer tissues, respectively.

Finally, to validate the expression of the above three prognostic-related genes at the tissue level, we selected the same immunofluorescence antibodies in both normal and tumor samples based on the HPA database (Table S11, Figure S1, S2, S3). And observe Immunohistochemistry images of CDKN2A (Figure 6 (A)-(D)), SERPINE1 (Figure 6 (F)-(I)), MMP3 (Figure 6 (K)-(N)), IOD values in five genes were differentially expressed in normal and cancer tissues (Figure 6 (E), 6 (J), 6 (O)). This suggests that these three genes could serve as therapeutic targets for CRC.

## Discussion

At present, tumor treatment methods containing surgical intervention, radiotherapy, chemical therapy, targeted therapy, biological therapy, immunization therapy, and traditional Chinese medicine as complementary and alternative medicine (CAM) therapy also play a significant role 48. Traditional Chinese medicine has the characteristics of diverse sources, high efficacy, and few toxic and side effects, and is widely welcomed in the world 49-53. Chinese herbal medicine plants contain a variety of active substances, which can effectively prevent and treat common clinical diseases. Due to the complex mechanism and progression of many diseases, traditional single-target drugs cannot cure some intricate diseases, such as cancer, angiocardiography, and neurological diseases. CHM contains a variety of active compounds, indicating that multi-components of herbal medicine have a synergistic action 54-55. Therefore, for the therapy of complex diseases for instance cancer, the combination of CHM compounds or combined chemotherapy drugs may be more suitable. It has been shown that a combination of curcumin, resveratrol, and quercetin can work together to treat cancer. For example, resveratrol combined with curcumin significantly bated the proliferation and induced apoptosis of hepatocellular carcinoma cells 56, and quercetin combined with resveratrol significantly increased the reversal of drug resistance of leukemic cells 57. Curcumin combined with quercetin significantly promoted tumor cell apoptosis in vivo 58.

Compared with monotherapy, Chinese herbal medicine is more suitable for multi-drug combinations. For example, HangAmDan-B1 (HAD-B1), consisting of four herbs—Panax notoginseng, Cordyceps militaris, ginseng, and frankincense—significantly inhibits HCC827-GR cell cycle arrest and accelerates apoptosis compared with monotherapy 59. Cepharanthine is an alkaloid extracted from Stephania cepharantha Hayata that has anti-inflammatory, and antioxidant effects, and can also induce autophagy, apoptosis, and cell cycle arrest in breast cancer cells 60. The main active compound of green tea, epigallocatechin-3-gallate (EGCG), has shown preventive effects in different cancer models 61-62. In addition, Ganoderma lucidum extract has also been used in traditional Chinese medicine to prevent or treat a variety of diseases 63-68.

Studies have claimed that green tea extract (GTE) and Ganodorma lucidum extract (GLE) can inhibit breast cancer cell proliferation, migration, and the combination of the two also has a synergistic effect, and the combination of low concentrations of GLE and GTE significantly inhibits the growth and invasion of MDA-MB-231 metastatic breast cancer 69. Studies have also shown that the appropriate addition of CHM or CHM compounds to the diet of chemotherapy-resistant patients can enhance immunity, reverse chemotherapy resistance, enhance the toxicity of drugs to cells, reduce side effects, and prolong the life of patients 70. Astragalus polysaccharides significantly reduced the side effects of vinorelbine and cisplatin in patients with advanced NSCLC and significantly improved their quality of life 71. In the combination therapy of Chinese herbal medicine, attention needs to be paid to the combination, because not all combinations are safe 72, and there are enormous differences between experimental data and clinical application. Therefore, it is necessary to use some methods to optimize the combination of traditional Chinese medicines, such as the research and analysis method of network pharmacology combined with bioinformatics to study, evaluate and analyze the synergistic effects of the combination of traditional Chinese medicines 73.

In this research, network pharmacology and combined bioinformatics were used to analyze the targets of traditional Chinese medicine extracts quercetin and kaempferol, construct and screen the interaction network between drugs and CRC pathogenic genes, and finally screen three prognostic related genes: CDKN2A, SERPINE1, and MMP3 74-79.

In recent years, the anticancer activity of quercetin has attracted widespread attention. Pharmacological studies have found that quercetin inhibits tumor growth and induces tumor autophagy and apoptosis by regulating various signaling pathways, for instance, PI3K-Akt signaling pathway, NF-κB signaling pathway, mTOR signaling pathway, MAPK signaling pathway 80, TGF-β1/Smad3/c-MYC pathway 81, EGFR signaling pathway 82 and Jak-STAT signaling pathway 83. Quercetin can inhibit tumor cell proliferation by inducing apoptosis in glioma 84, melanoma 85, and bladder cancer 86. It can also inhibit tumor growth by inhibiting angiogenesis 87 or inhibit cell proliferation by inducing autophagy of tumor cells, such as human glioma cells, primary lymphoma cells, ovarian cancer cells, human leukemia cells, etc., such as 88-90. Cancer cell metastasis is associated with cell migration and invasion and is a major cause of death and poor prognosis in patients 91. Studies have shown that quercetin can bate the migration and invasion of rectal carcinoma and head and neck squamous cell carcinoma 92-93, lung cancer cells 94, pancreatic cancer cells 95, melanoma cells 96, thereby preventing the metastasis of cancer cells. Not only that, but quercetin can also improve sensitivity and reduce the side effects of radiation and chemotherapy 97.

Quercetin not only exerts an antitumor effect alone, but also exerts an antitumor effect in combination with imatinib, inhibits the activity of cancer cells 98, improves the sensibility of cancer cells to radiotherapy 99, and affects p-glycoprotein (P-gp) to reverse the drug tolerance of cancer cells 100. The combination of quercetin and gemcitabine also improves outcomes in patients with pancreatic cancer 101. These results show that quercetin has a good anticancer effect and is used in clinical treatment in combination with other drugs as anticancer sensitizers.

Kaempferol is widely used in medicine for its antioxidant, anti-inflammatory, antibacterial, anticancer, heart, and neuroprotective effects 102. Kaempferol protects normal cells, prevents normal cells from transforming into tumor cells 103, and inhibits the proliferation of cancer cells by increasing the apoptosis of tumor cells 104. Not only that, but kaempferol also induces cell death by inducing autophagy 105. Kaempferol alleviates oxidized LDL ox-LDL-induced apoptosis by participating in various signaling pathways, such as inhibition of AKT signaling-induced cell death in ovarian cancer 106, inhibition of the PI3K/Akt/mTOR pathway, thereby alleviating oxidized LDL ox-LDL-induced apoptosis 107, and mitigating palmitate acid-induced lipid storage, endoplasmic reticulum stress, and pancreatic β cell dysfunction through mTOR-mediated autophagy pathway 108. It exerts antitumor effects by regulating TNF-α, NF κB pathway 109. In addition, kaempferol inhibits the conversion of aromatic hydrocarbon receptor AHR 110 and also inhibits cell proliferation in breast and prostate cancer 111. Kaempferol can reverse tumor resistance by inhibiting P-gp 112-113 and exert adjunctive antitumor effects by increasing the sensitivity of cisplatin and 5-fluorouracil 114-115. Furthermore, epidemiological studies suggest a correlation between kaempferol intake and the danger of multi-tumors 116-117. Kaempferol prevents tumor invasion and metastasis. High levels of MMP3 are associated with stronger tumor aggressiveness and poor prognosis. Most flavonoids do not affect MMP3 secretion, but kaempferol significantly inhibits the migration of human breast cancer cells and is dose-dependent 118. These results reveal that kaempferol plays a crucial role in tumor apoptosis, autophagy, proliferation, drug resistance, invasion, and migration.

Quercetin and kaempferol have many of the same targets, such as AKT1, AHR, BCL2, NR4A1, etc., and AHR can inhibit the development of colorectal cancer 119-121. In rhabdomyosarcoma, they can inhibit NR4A1-dependent reverse transcription activation and downstream target genes and inhibit the growth of xenograft rhabdomyosarcoma in nude mice 122. This suggests that in colorectal cancer, the combination of the two may promote the expression of AHR, inhibit the expression of NR4A1, and achieve the synergistic effect of inhibiting CRC. However, due to the interaction between target proteins, after screening by the Cytoscape plugin, most of the target proteins, such as NR4A1, BCL2, CDK1, NOS2, and MMP1, etc., are excluded, but the role of these proteins cannot be ignored, and in the screening process, there are multiple mechanisms of action between protein targets and genes, which cannot be “consistent”.

Although quercetin and kaempferol alone can enhance anti-cancer, and non-specific immunity, and reduce the immunosuppressive effects, side effects, and precancerous lesions, the combination of quercetin and kaempferol can also increase CRC cells 26 of cell cycle arrest, but whether their combined use reduces the therapeutic burden is unclear. We study this through a bioinformatics database to understand the molecular and genetic pathways of these two drugs in vivo. This study preliminarily explored the molecular mechanisms of these two components in the treatment of CRC, laying a foundation for further studies of their biomolecular mechanisms. From the results of the gene function analysis, it can be seen that the combination of quercetin and kaempferol is effective in the treatment of colorectal cancer because they have similar roles in multiple molecular pathways and gene functions, for instance inhibiting the expression of MMP3 123-128. However, in CRC, whether kaempferol can inhibit MMP3 activity, thereby affecting the development, progression, and migration of CRC? Although there is no immediate proof at present, we expect to verify it through pharmacological experiments later, and based on the molecular docking prediction map of kaempferol and MMP3, we also doubt that kaempferol might directly interact with MMP3, which could become a novel finding in this study.

Moreover, we applied Lasso-Cox regression analysis to build a prognostic analysis model of Lasso-Cox regression with risk scores for evaluating patients in CRC two-component therapy or planned two-component therapy. The risk score and prognostic survival rate of patients are obtained through this model. We can predict risk scores and prognostic survival rates through the nomogram of this prognostic model to conduct clinical therapeutic strategies. Through bioinformatics analysis, it was found that three prognostic genes of the combination of quercetin and kaempferol in the treatment of CRC were positively correlated with immune infiltration. The expression differences of these three prognostic genes were found by plotting the expression levels of immunohistochemical genes in the cancer group and the normal group in the HPA database. Accordingly, it is reasonable to believe that this predictive model is accurate. This study shows that the same regimen of quercetin with kaempferol may have better results in high-risk patients; that is, immune cell levels may be higher in high-risk patients than in low-risk patients, and the expression of immune checkpoints is more pronounced. Thus, the risk values obtained from this model can guide clinical immunotherapy. In the CRC tumor microenvironment, patients with deeper immune invasion have higher survival rates. In practice, the combination of quercetin and kaempferol may help improve the effectiveness of immunotherapy in patients with CRC.

## Conclusions

In conclusion, this study analyzed the molecular mechanism of quercetin and kaempferol in the therapy of CRC and confirmed the synergistic effect of quercetin and kaempferol in the therapy of CRC. Formerly studies showed that these two components have similar effects, there is also overlap between targets, and both can inhibit the role of P-gp to reverse tumor drug resistance and inhibit some signal pathways, such as P13K/AKT signaling pathway, EGFR signaling pathway, mTOR signaling pathway, MAPK signaling pathway, Jak-STAT signaling pathway, and NF-κB signaling pathway et al. Thus, there is reason to believe that quercetin and kaempferol can exert a synergistic effect at the level of molecular pathways.

In this study, we applied modern network pharmacology combined with bioinformatics to analyze the molecular mechanism of quercetin and kaempferol in the treatment of CRC, innovatively established a Lasso-Cox prognostic regression model, obtained three prognosis-related genes, and analyzed the immunotherapy effect of the two drugs. These studies can help to guide clinical application. We hope to next verify the conclusions of this study by using pharmacology, molecular biology, and cell function experiments in later studies to make the conclusions more accurate and dependable. Some deficiencies still exist in this study and are expected to be supplemented in subsequent studies.

## Supplementary Material

Supplementary figure 1.

Supplementary figure 2.

Supplementary figure 3.

Supplementary tables.

## Abbreviations

CRC: Colorectal cancer
TCM: Traditional Chinese medicine
TCMSP: Traditional Chinese Medicine Systems Pharmacology Database and Analysis Platform
TCGA: The Cancer Genome Atlas
GEO: Gene Expression Omnibus
RNA-seq: RNA sequencing
ICI: Immune checkpoint inhibitors
NAFLD: Nonalcoholic fatty liver disease
CHM: Chinese herbal medicine
GSEA: Gene Set Enrichment Analysis
OMIM: Online Mendelian Inheritance in Man
PharmGkb: Pharmacogenetics and Pharmacogenomics Knowledge Base
DisGeNET: a database of gene-disease associations
TTD: Therapeutic Target Database
HPA: The Human Protein Atlas
PPI: protein-protein interaction
STRING: search tool for retrieval
KEGG: Kyoto Encyclopedia of Genes and Genomes
GO: Gene ontology
ROC: receiver operating characteristic curve
IOBR: Multi-Omics Immuno-Oncology Biological Research
ESTIMATE: Estimation of STromal and Immune cells in MAlignant Tumor tissues using Expression data
coef: coefficient
TIMER2.0: Tumor Immune Estimation Resource
AUC: Area Under Curve
CAM: Complementary and Alternative Medicine
EGCG: epigallocatechin-3-gallate
GTE: Green tea extract
GLE: Ganodorma lucidum extract
NSCLC: Non-Small Cell Lung Carcinoma
P-gp: P-glycoprotein
