# Supplementary material for: Network pharmacology and bioinformatics were used to construct a prognostic model and immunoassay of core target genes in the combination of quercetin and kaempferol in the treatment of colorectal cancer
Source: J Cancer. 2023 Jul 3;14(11):1956–80. doi: 10.7150/jca.85517 (PMC10367918; doi:10.7150/jca.85517)

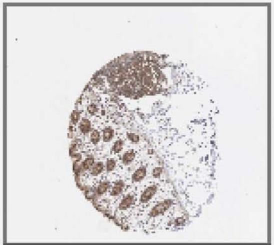

Colon

CAB000445

Male, age 14
Colon (T-67000)
Normal tissue, NOS (M-00100)
Patient id: 1990

Endothelial cells

Staining: **Low**

Intensity: **Weak**
Quantity: **>75%**
Location: **Nuclear**

Glandular cells

Staining: **High**

Intensity: **Strong**
Quantity: **>75%**
Location: **Cytoplasmic/ membranous nuclear**

Peripheral nerve/ganglion

Staining: **High**

Intensity: **Strong**
Quantity: **>75%**
Location: **Cytoplasmic/ membranous nuclear**

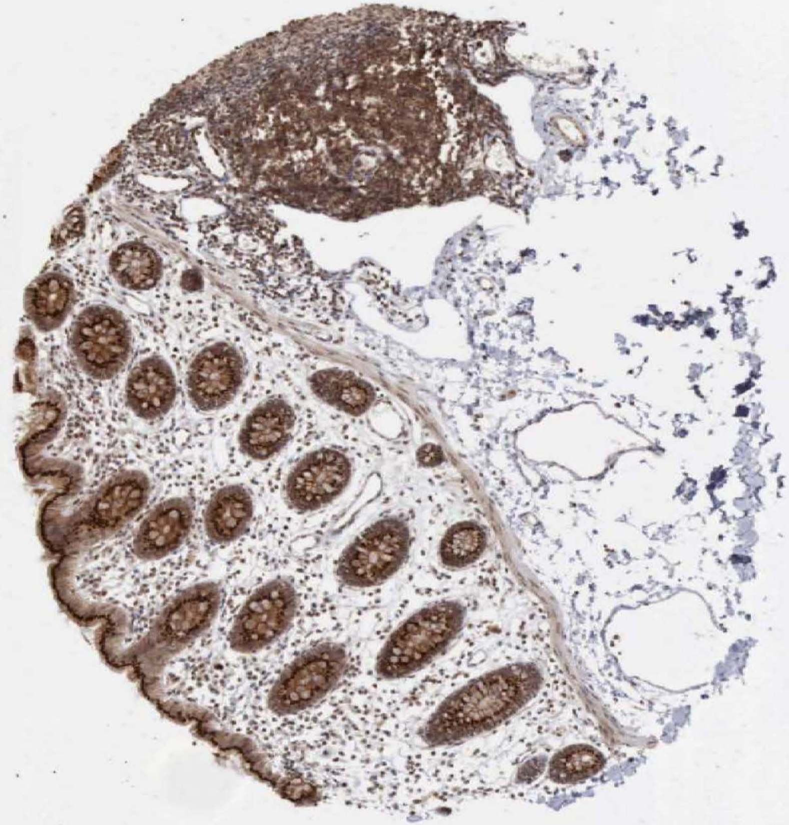

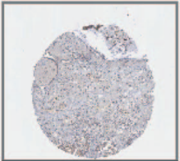

Colorectal cancer

CAB000445

Female, age 84
Colon (T-67000)
Adenocarcinoma, NOS
(M-81403)
Patient id: 1958

Tumor cells

Staining: **Medium**

Intensity: **Moderate**
Quantity: **75%-25%**
Location: **Cytoplasmic/ membranous**

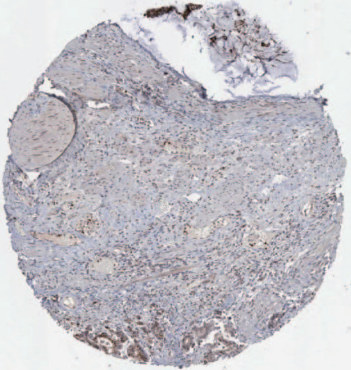

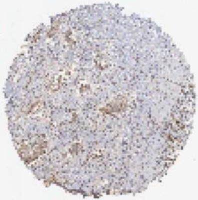

Colorectal cancer

CAB000445

Male, age 53
Rectum (T-68000)
Adenocarcinoma, NOS
(M-81403)
Patient id: 1424

Tumor cells

Staining: **Medium**

Intensity: **Moderate**
Quantity: **>75%**
Location: **Cytoplasmic/ membranous**

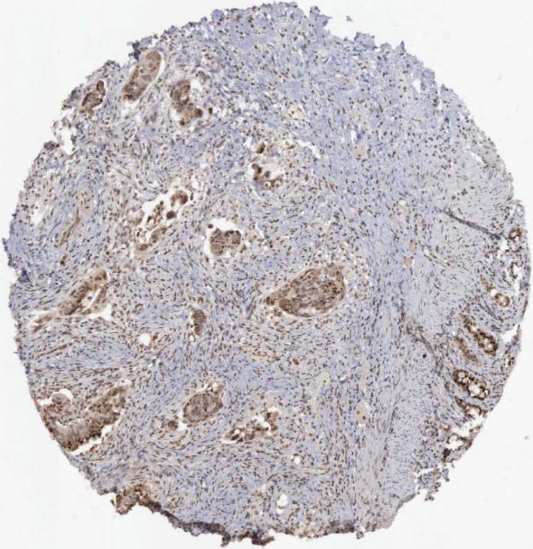

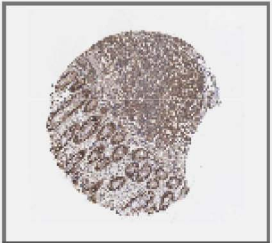

Rectum

CAB000445

Female, age 66
Rectum (T-68000)
Normal tissue, NOS (M-00100)
Patient id: 2060

Glandular cells

Staining: **Medium**

Intensity: **Moderate**
Quantity: **>75%**
Location: **Cytoplasmic/ membranous nuclear**

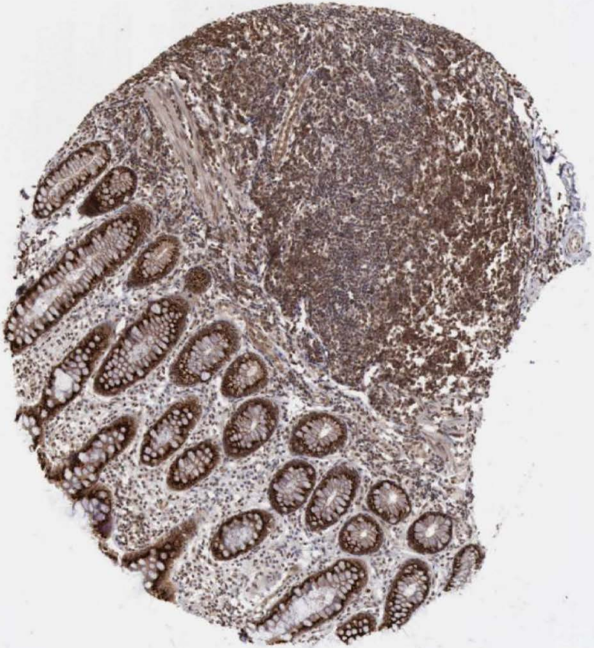

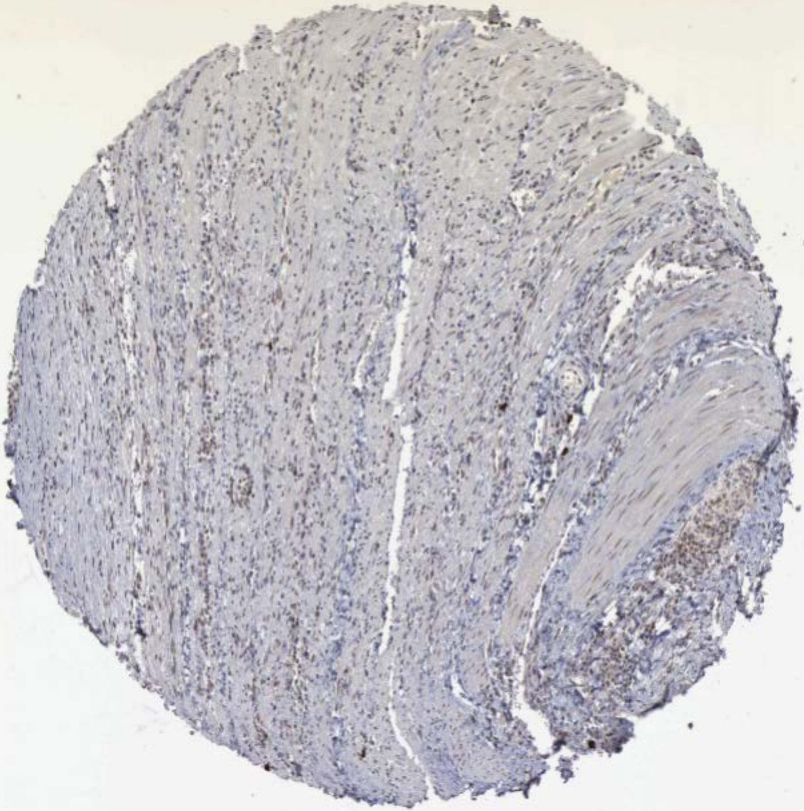

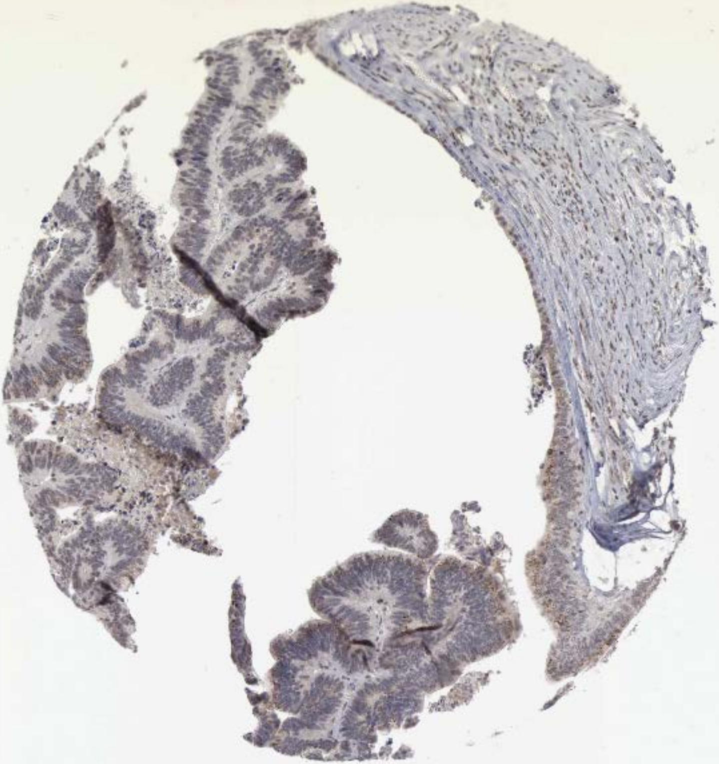

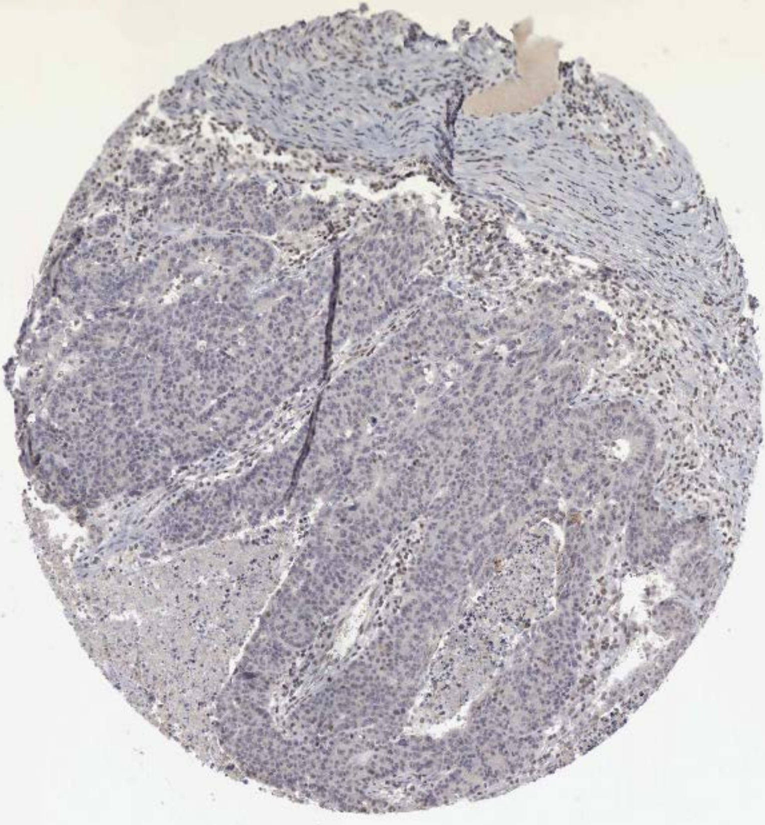

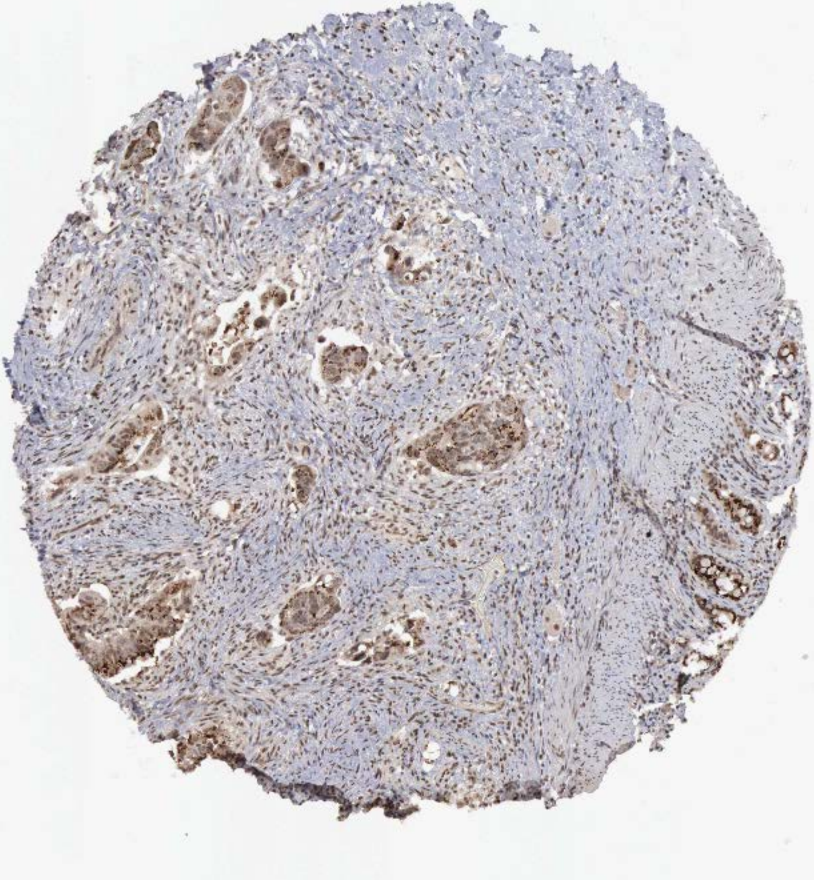

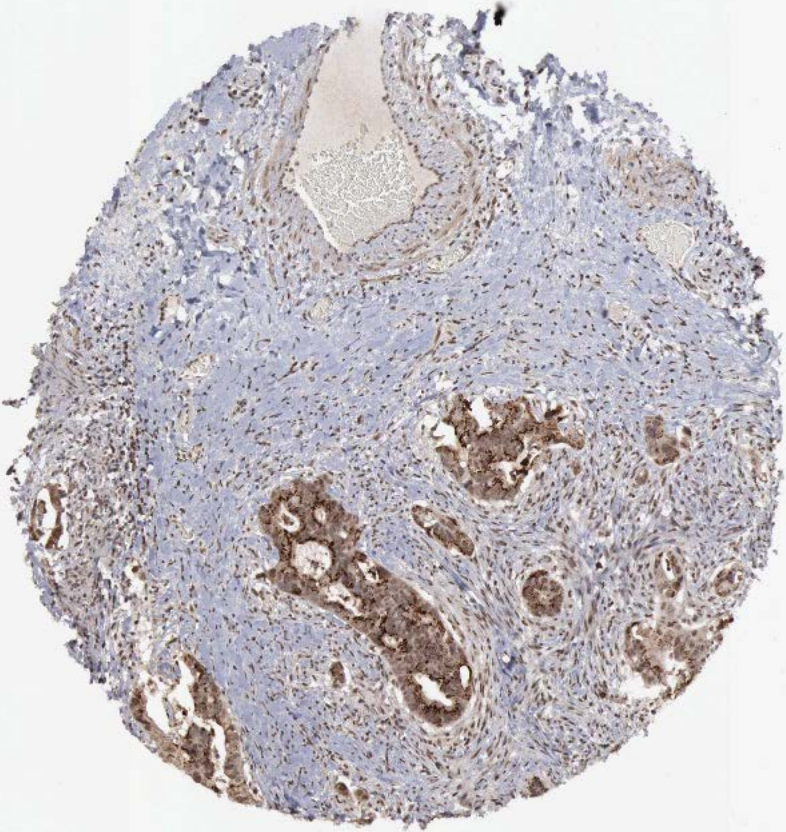

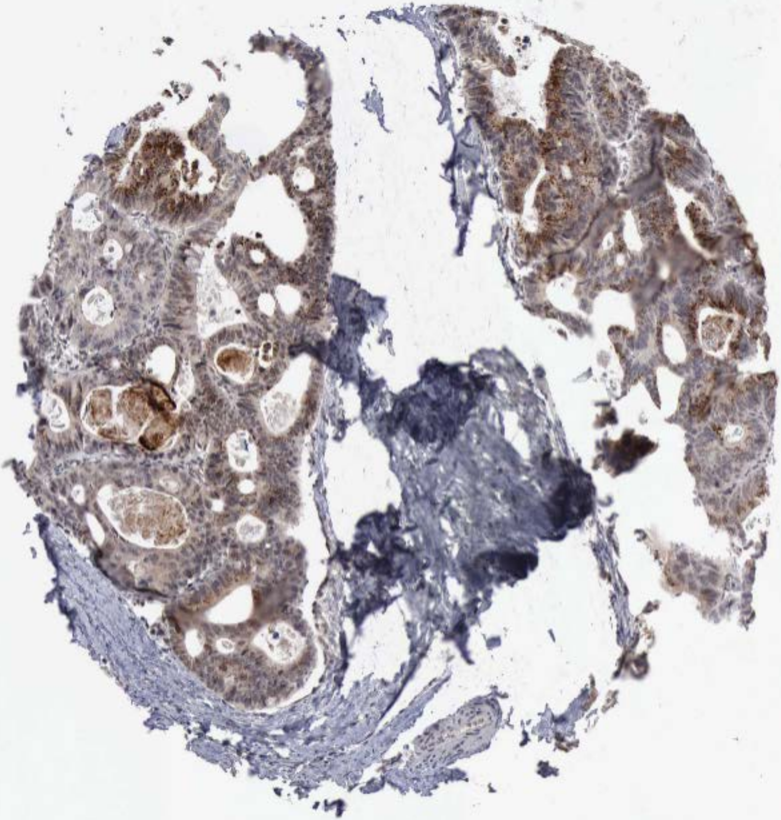

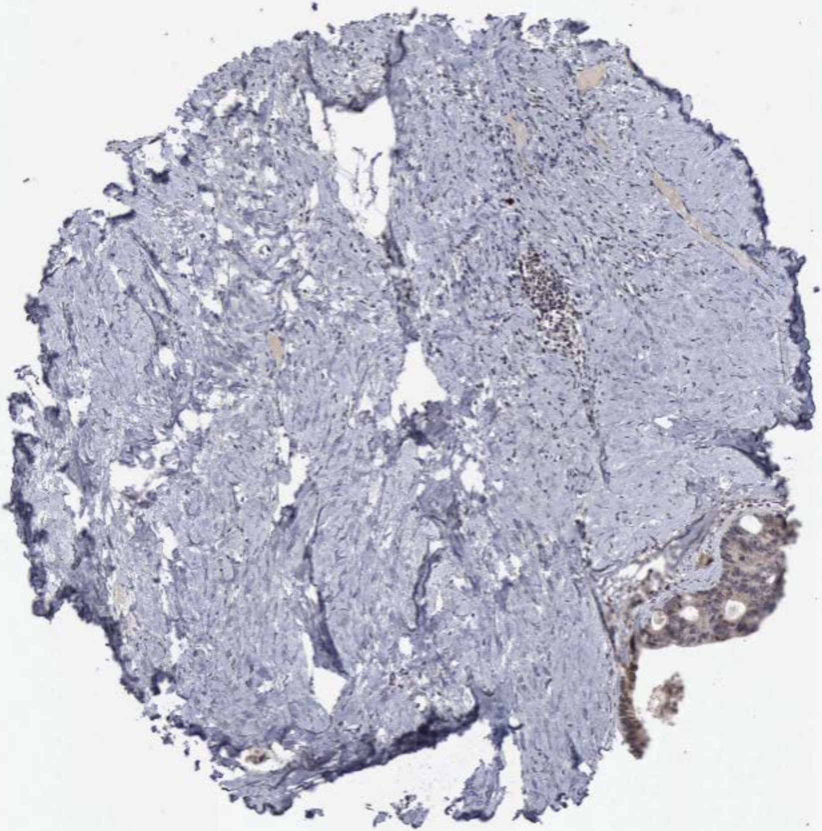

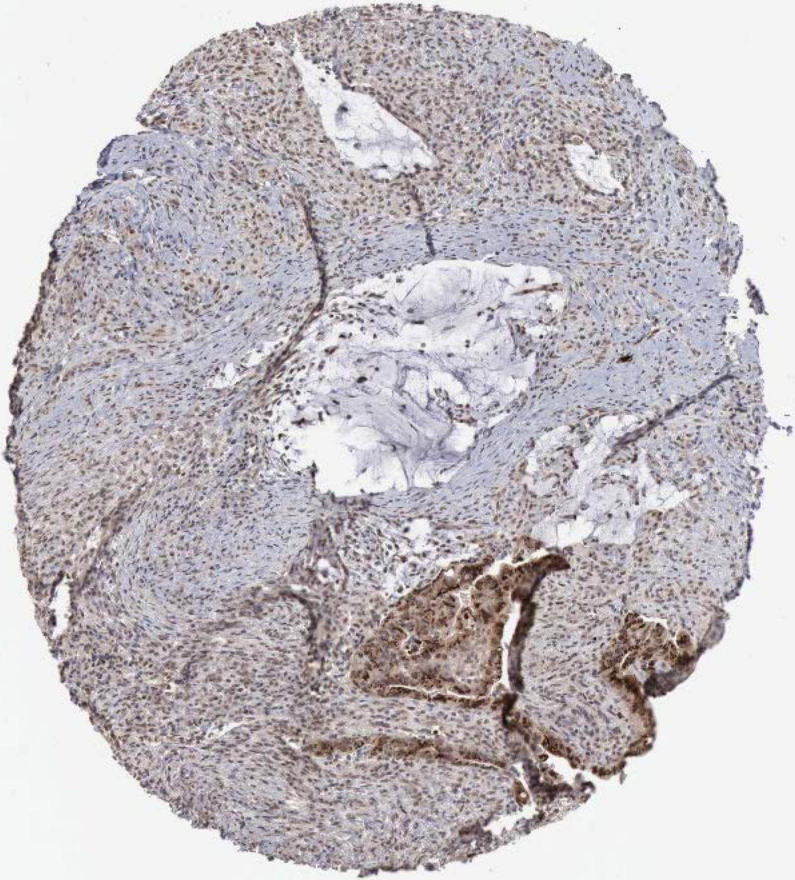

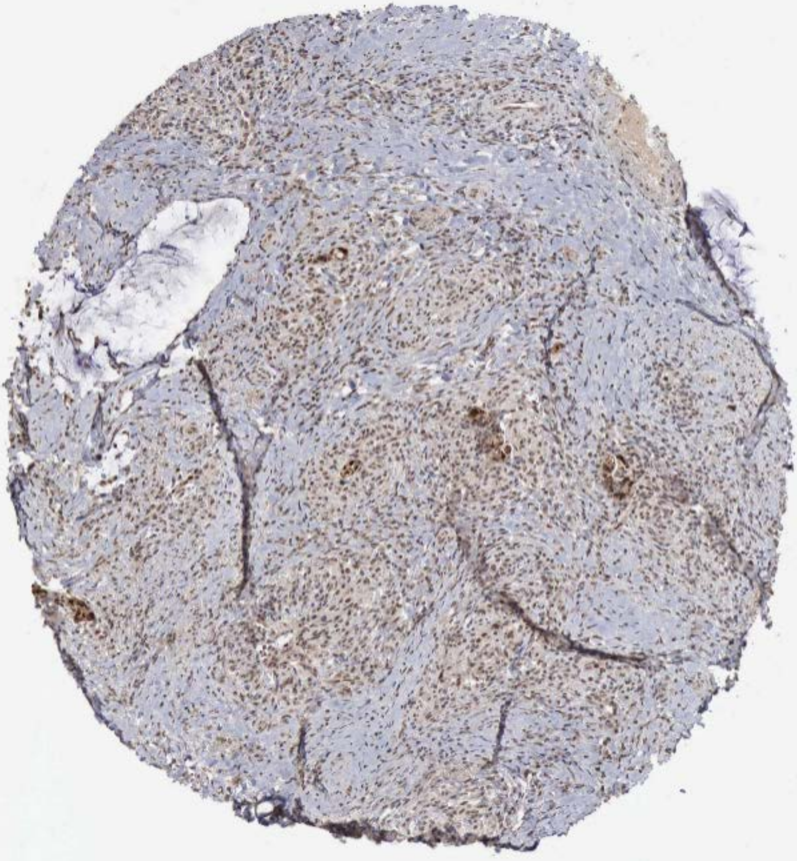

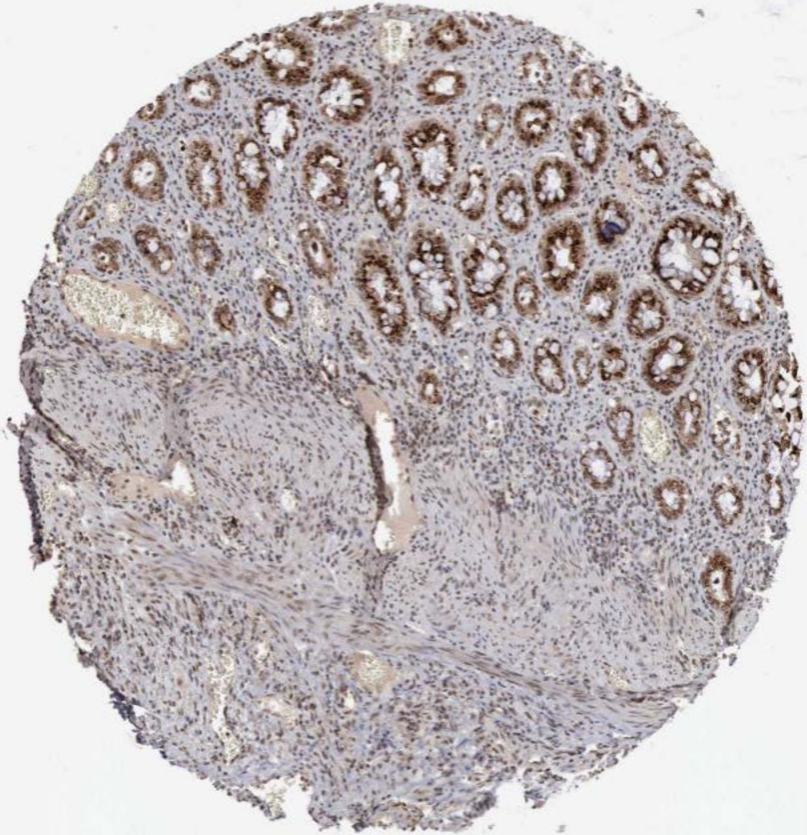

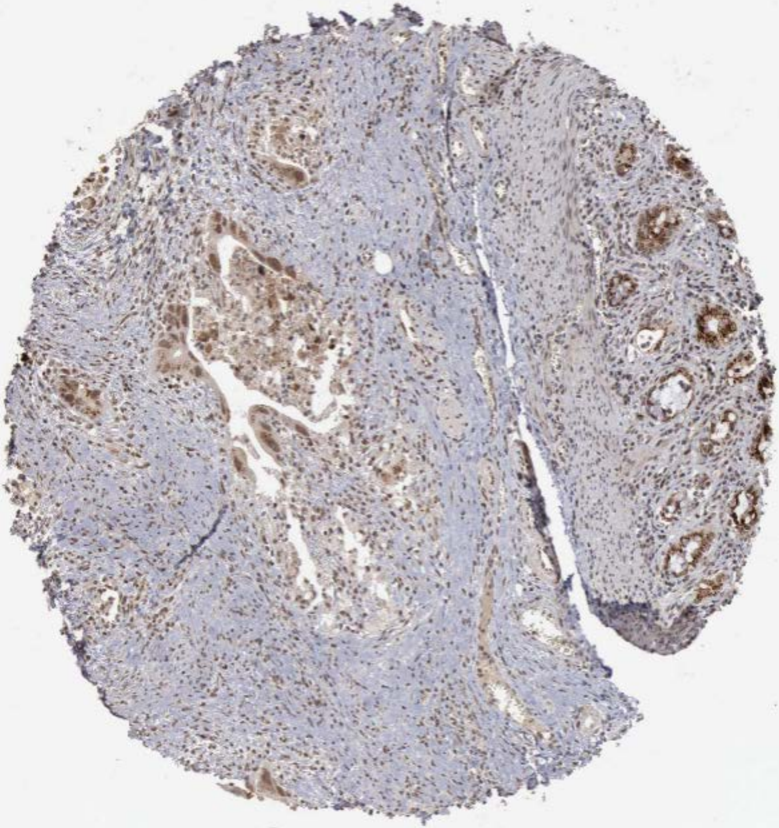

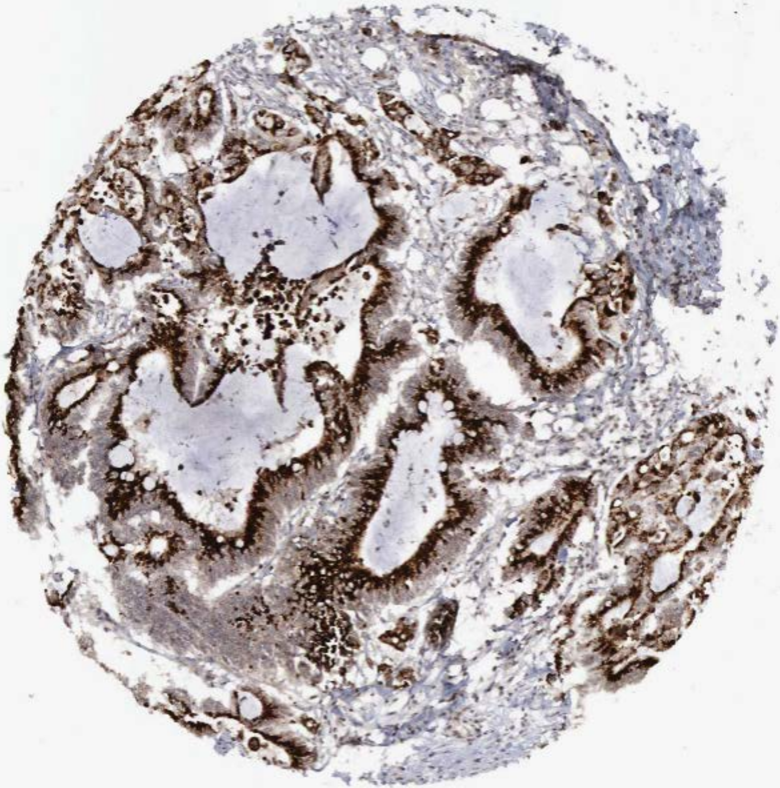

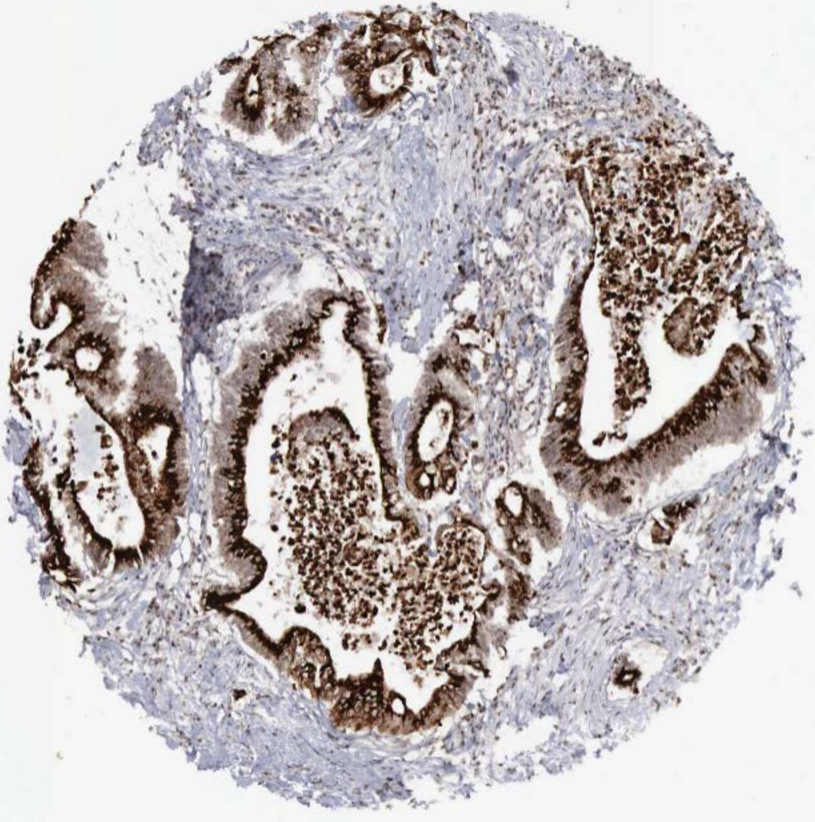

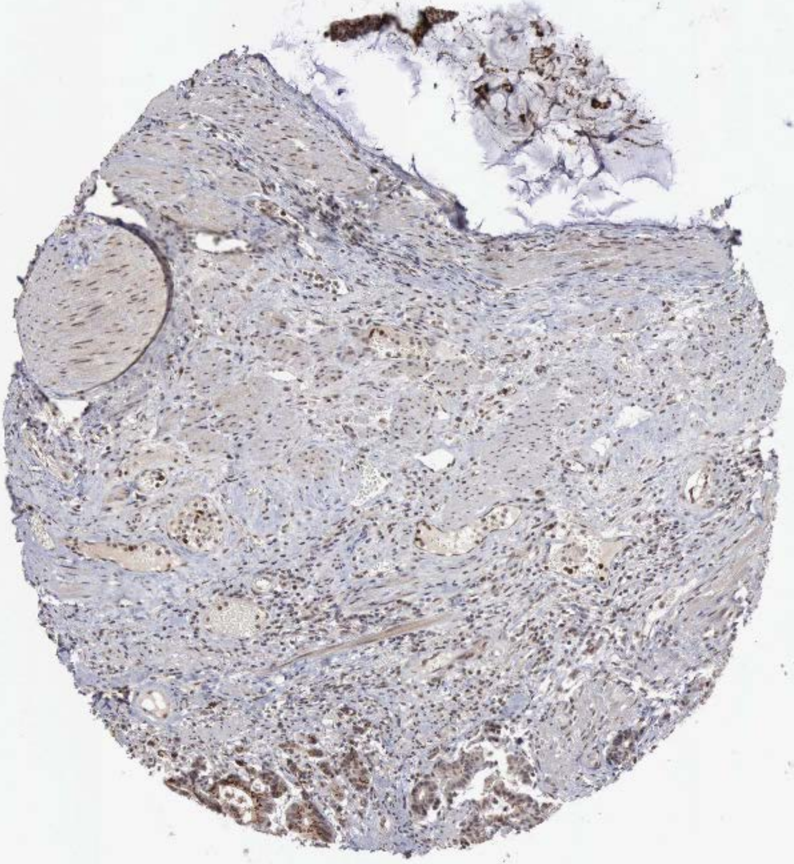

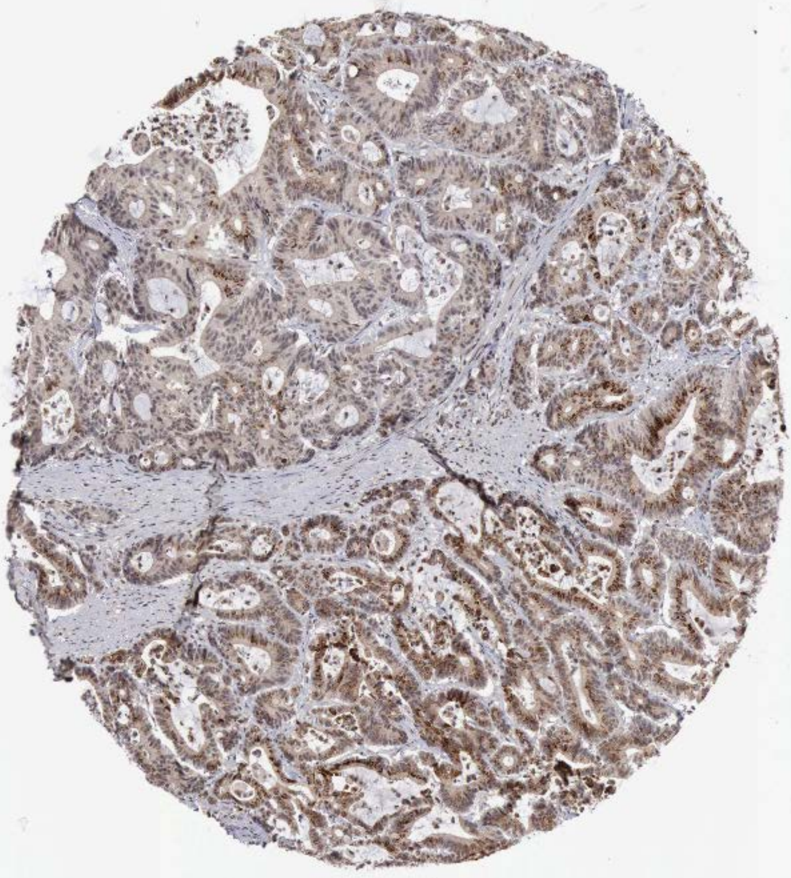

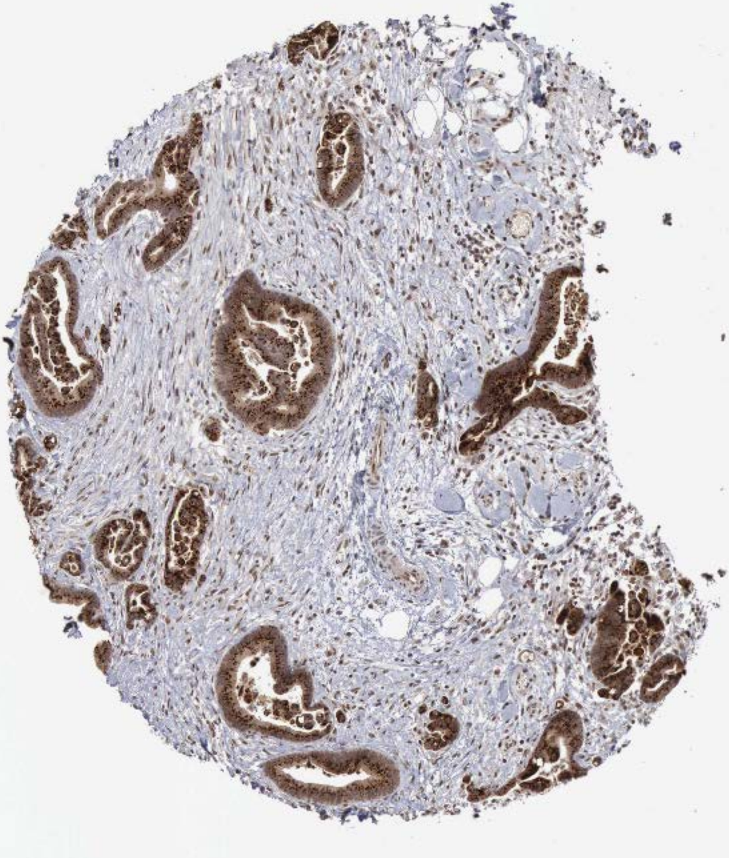

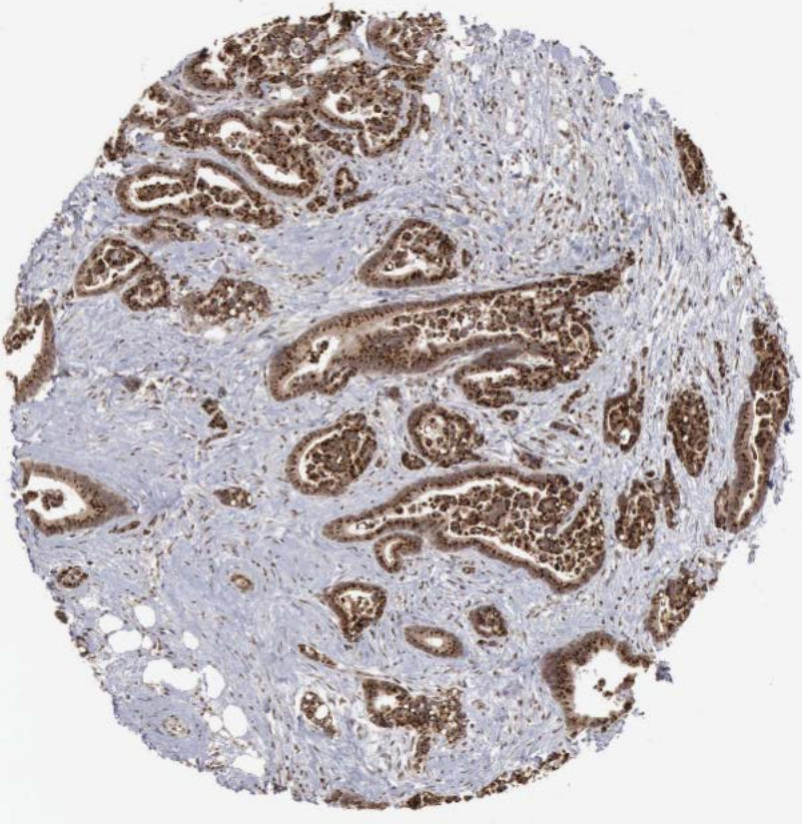

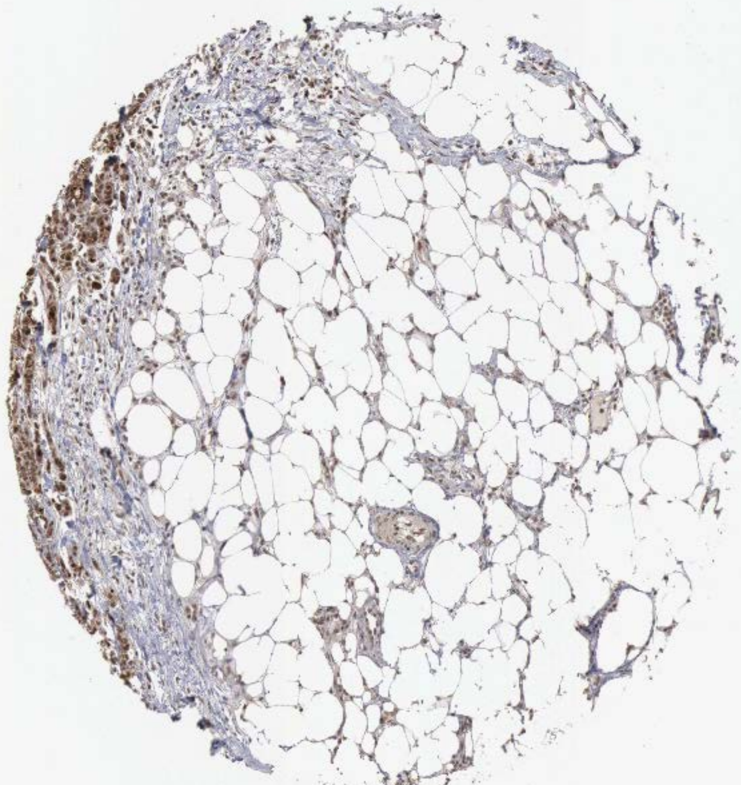

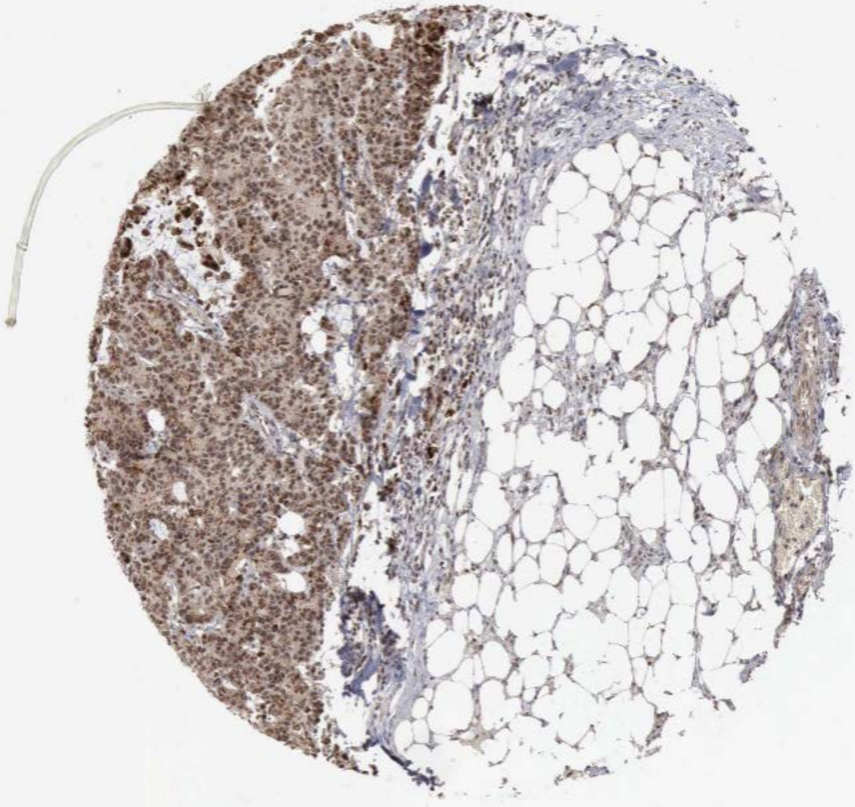

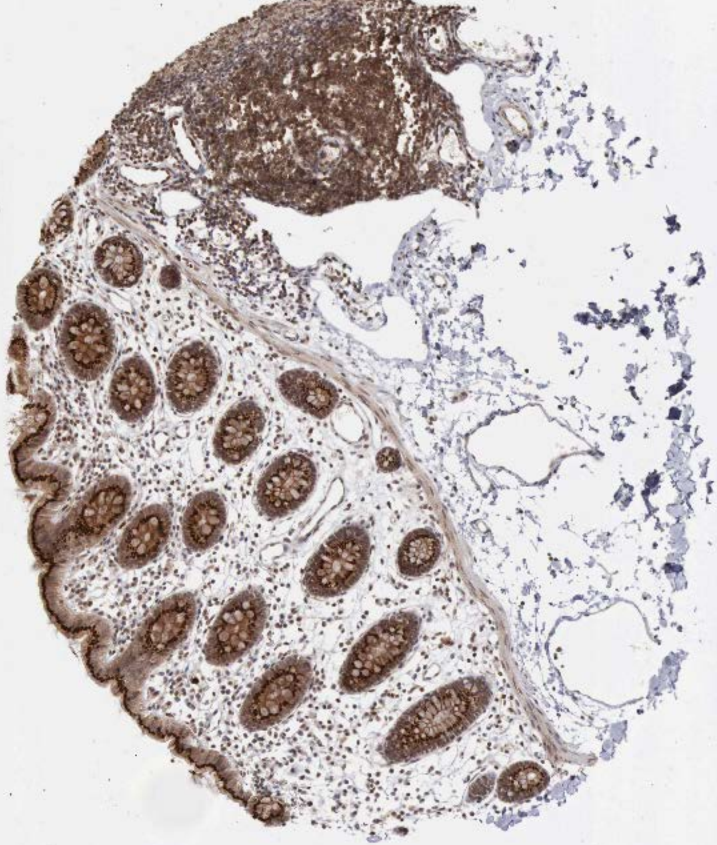

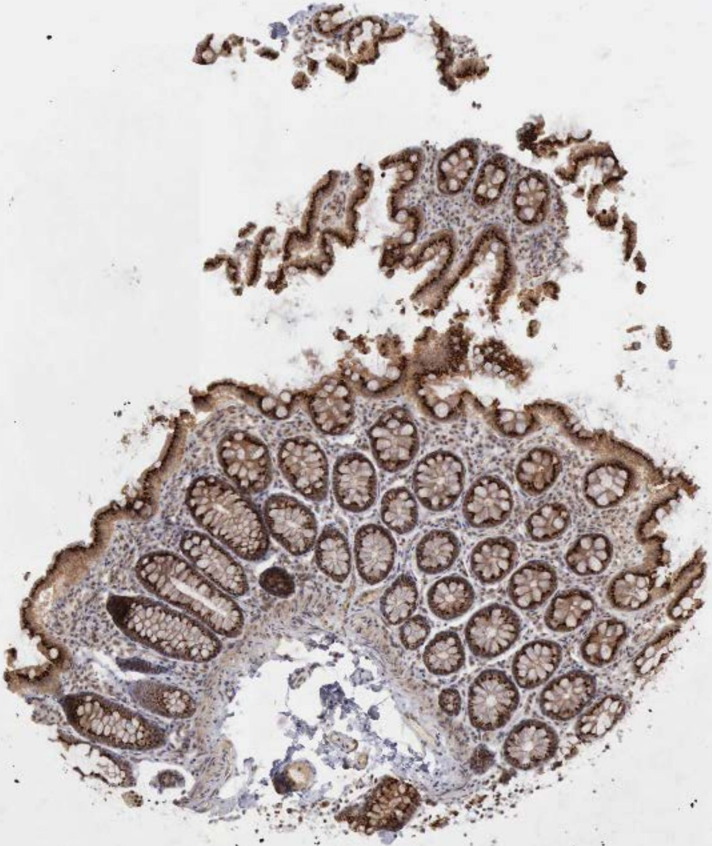

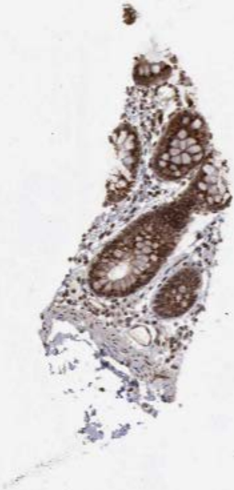

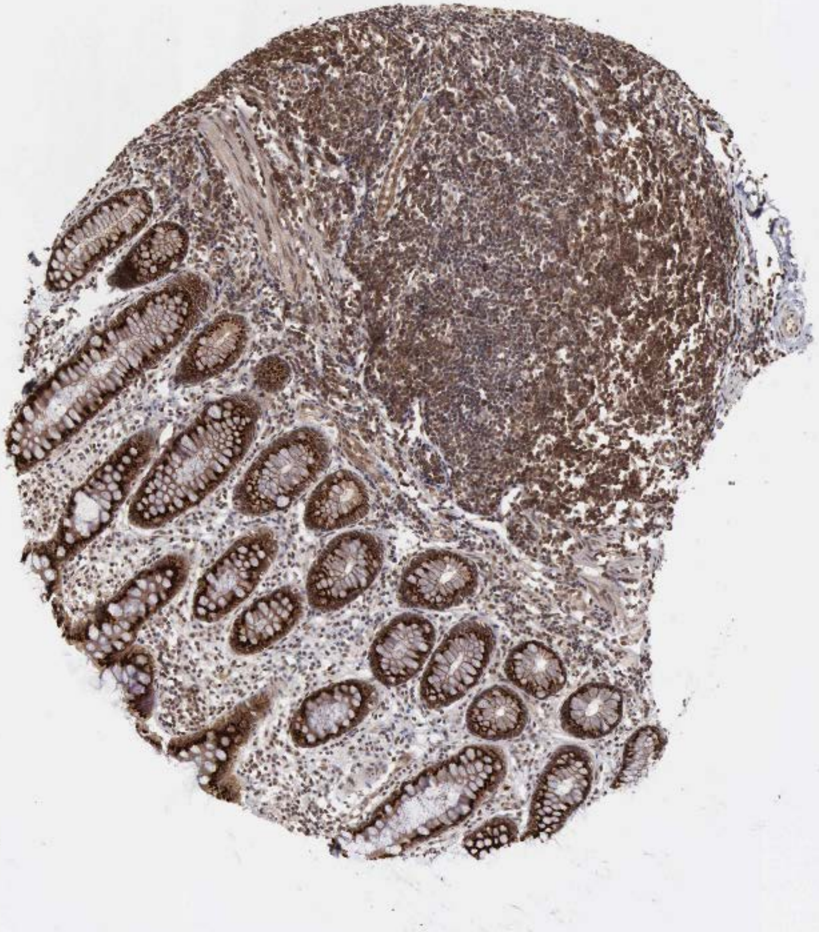

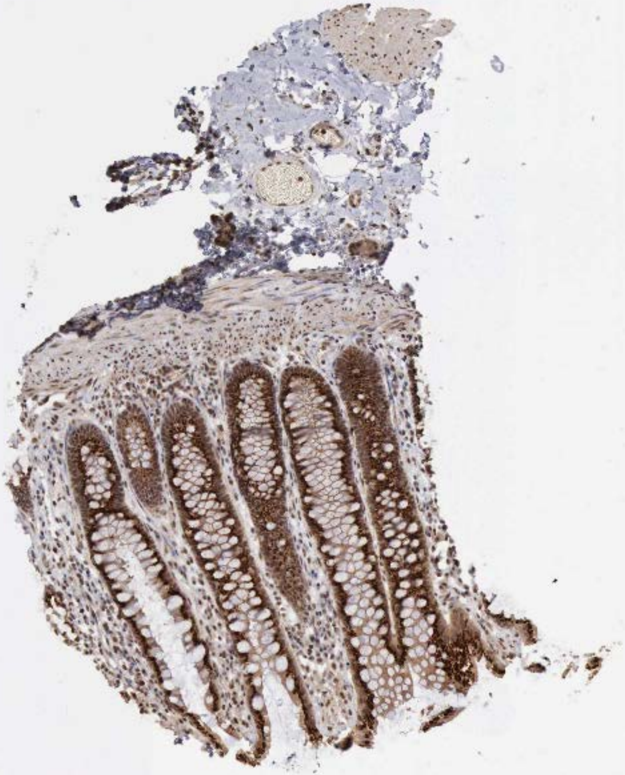

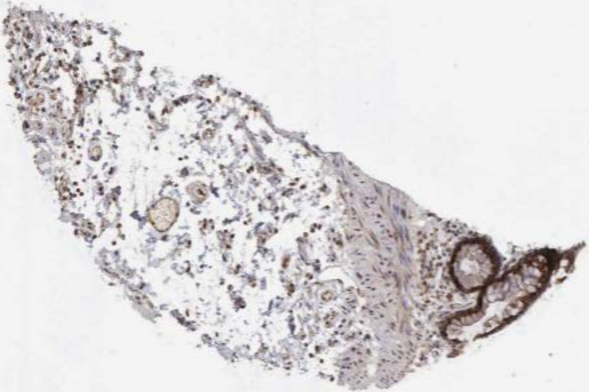

Supplement: Supplementary file 1 — Supplementary figure 1. [file jcav14p1956s1.pdf]
