# Supplementary material for: Network pharmacology and bioinformatics were used to construct a prognostic model and immunoassay of core target genes in the combination of quercetin and kaempferol in the treatment of colorectal cancer
Source: J Cancer. 2023 Jul 3;14(11):1956–80. doi: 10.7150/jca.85517 (PMC10367918; doi:10.7150/jca.85517)

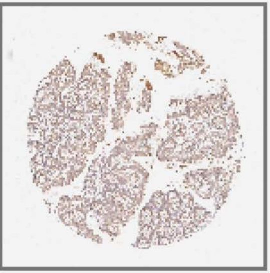

Colon

CAB068501

Female, age 84
Colon (T-67000)
Normal tissue, NOS (M-00100)
Patient id: 1958

Endothelial cells

Staining: **Low**

Intensity: **Weak**
Quantity: **>75%**
Location: **Cytoplasmic/ membranous nuclear**

G[illegible]lar cells

Staining: **Low**

Intensity: **Weak**
Quantity: **75%-25%**
Location: **Cytoplasmic/ membranous**

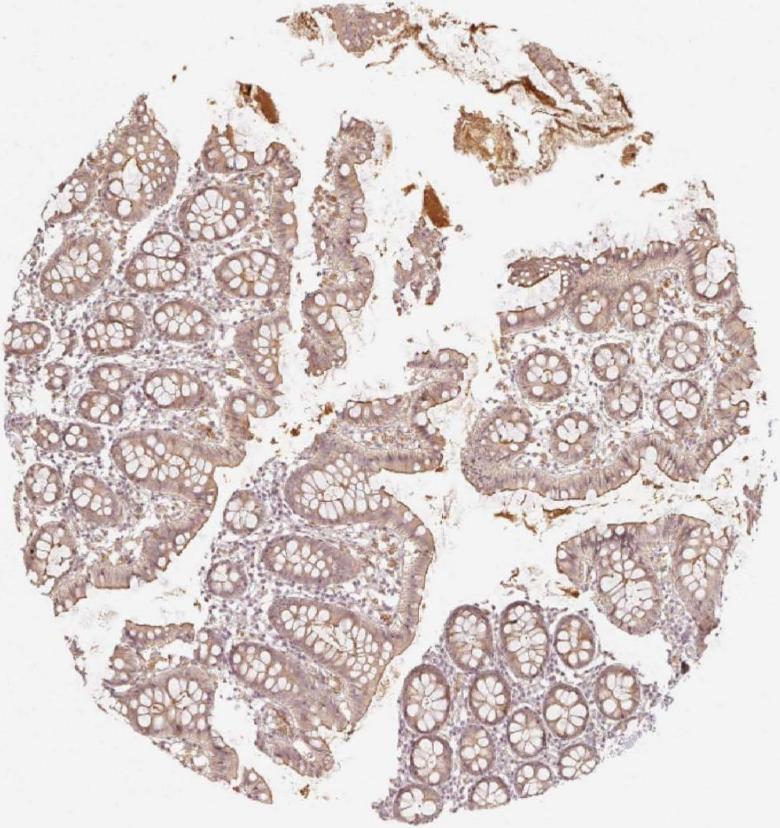

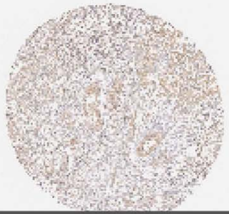

Colorectal cancer

CAB068501

Male, age 77
Colon (T-67000)
Adenocarcinoma, NOS
(M-81403)
Patient id: 2931

Tumor cells

Staining: **Not detected**

Intensity: **Negative**
Quantity: **None**

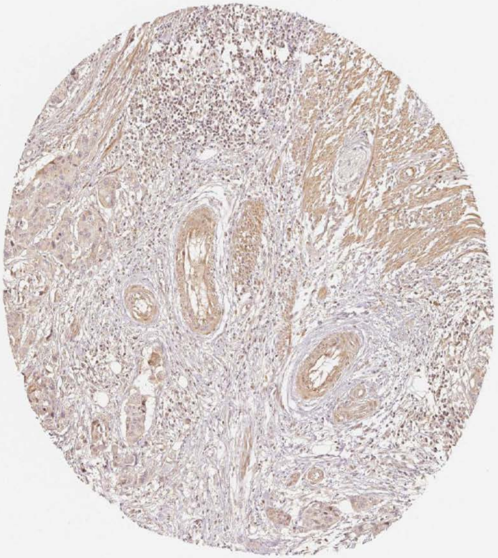

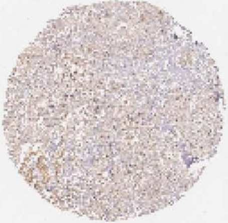

Colorectal cancer

CAB068501

Female, age 62
Rectum (T-68000)
Adenocarcinoma, NOS
(M-81403)
Patient id: 4752

Tumor cells

Staining: **Not detected**

Intensity: **Negative**
Quantity: **None**

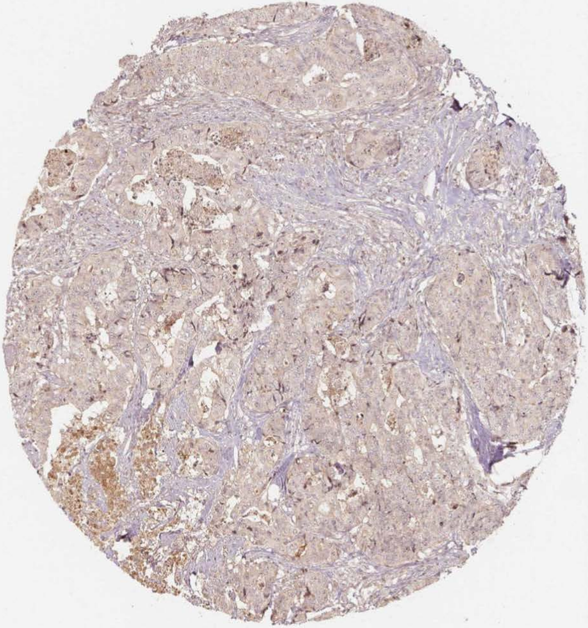

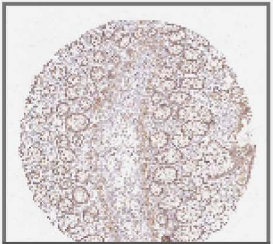

Rectum

CAB068501

Female, age 62
Rectum (T-68000)
Normal tissue, NOS (M-00100)
Patient id: 4381

Glandular cells

Staining: **Not detected**

ntensity: **Weak**
Quantity: **<25%**
Location: **Cytoplasmic/ membranous**

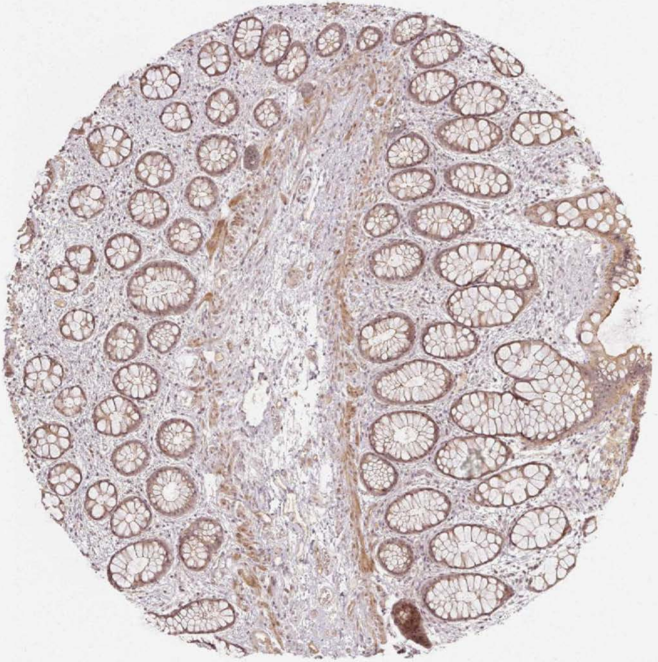

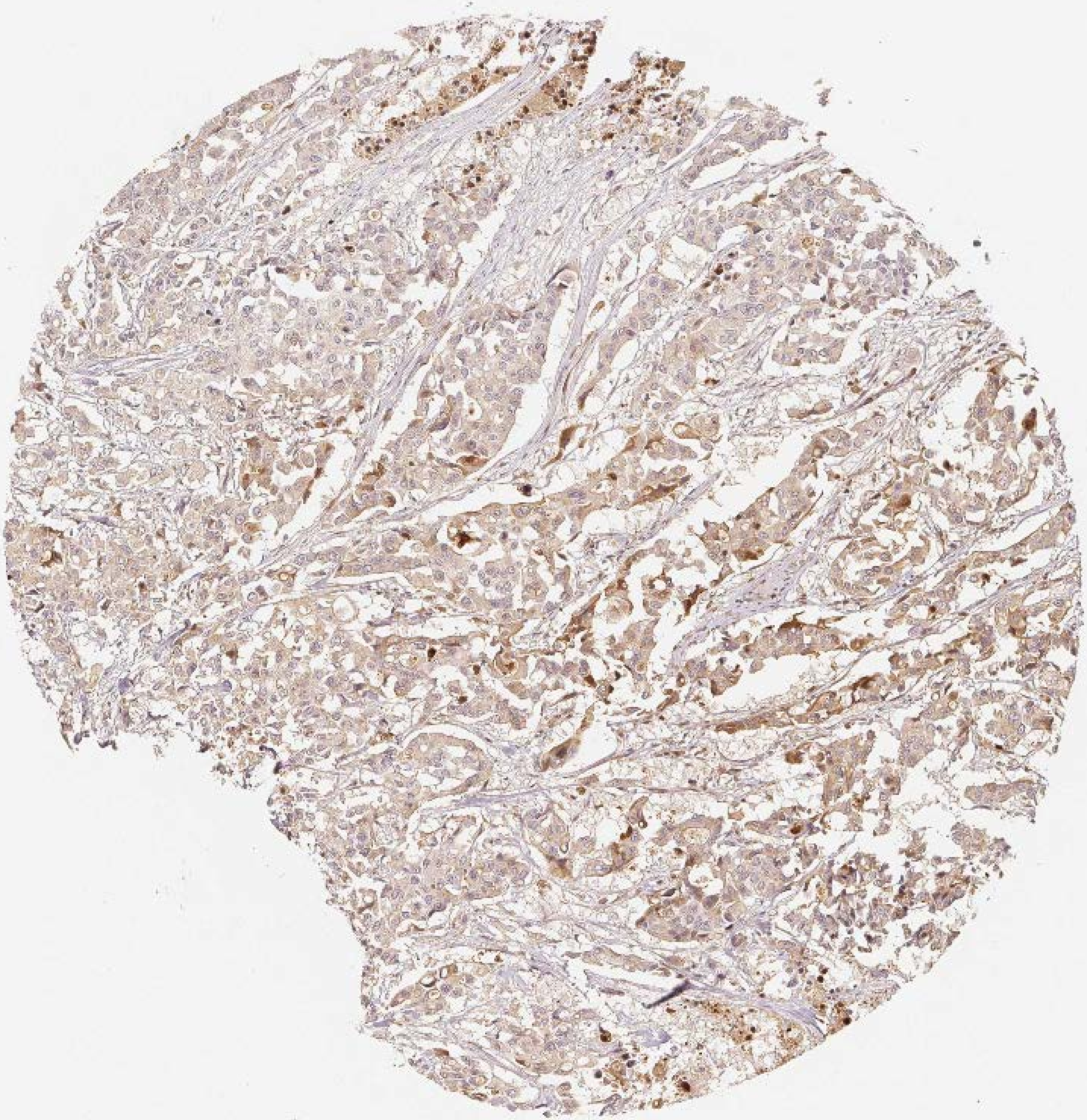

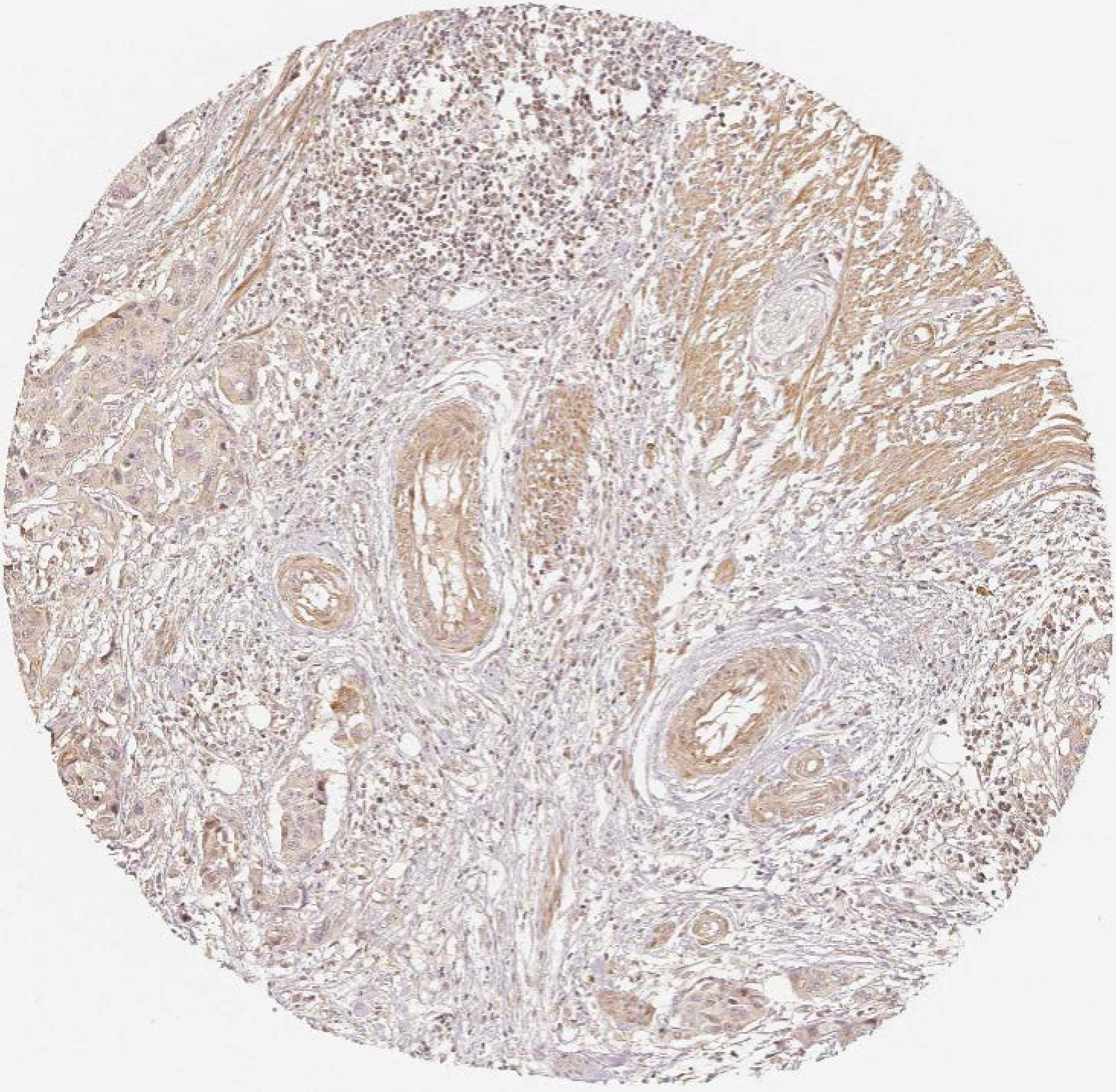

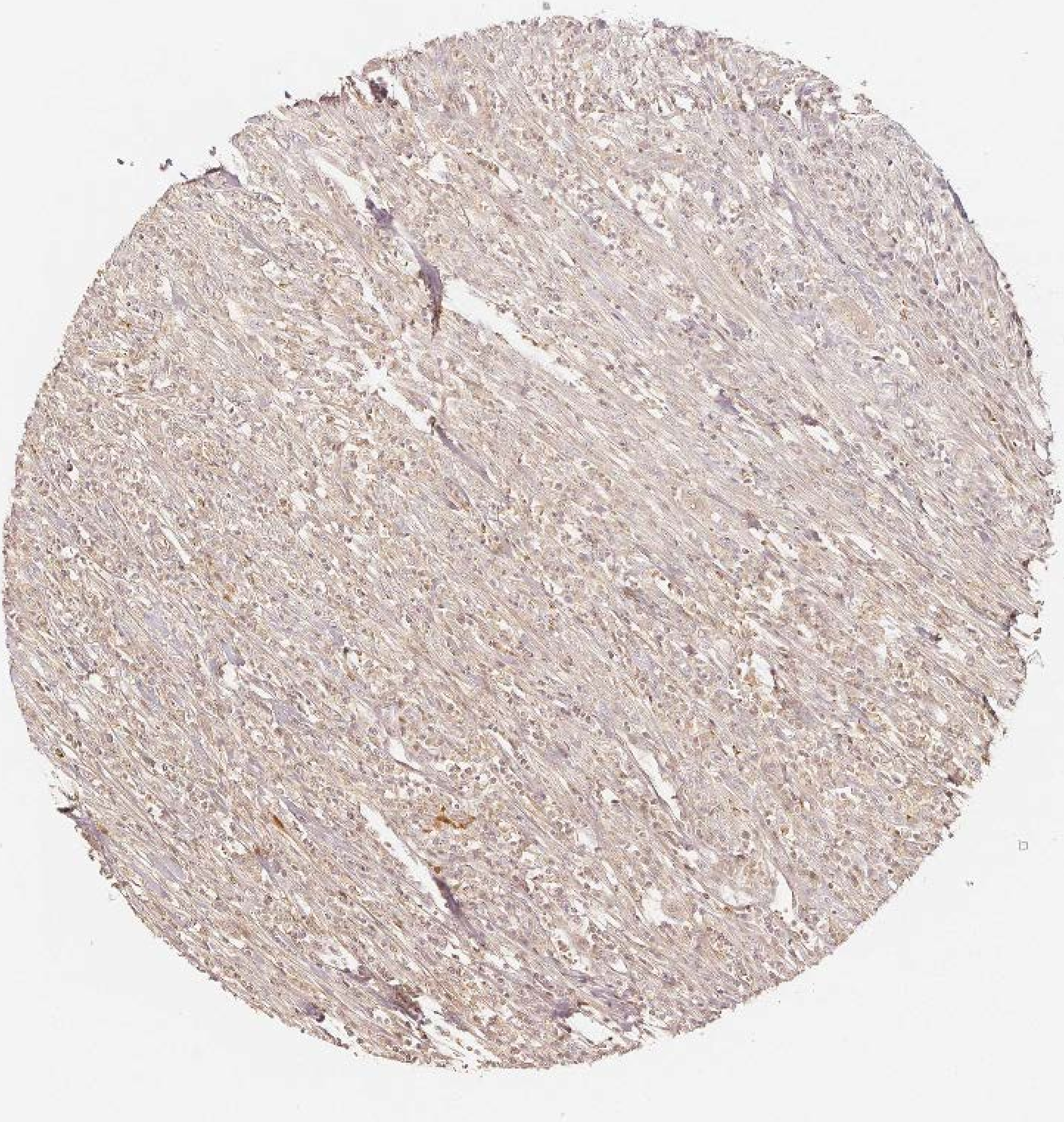

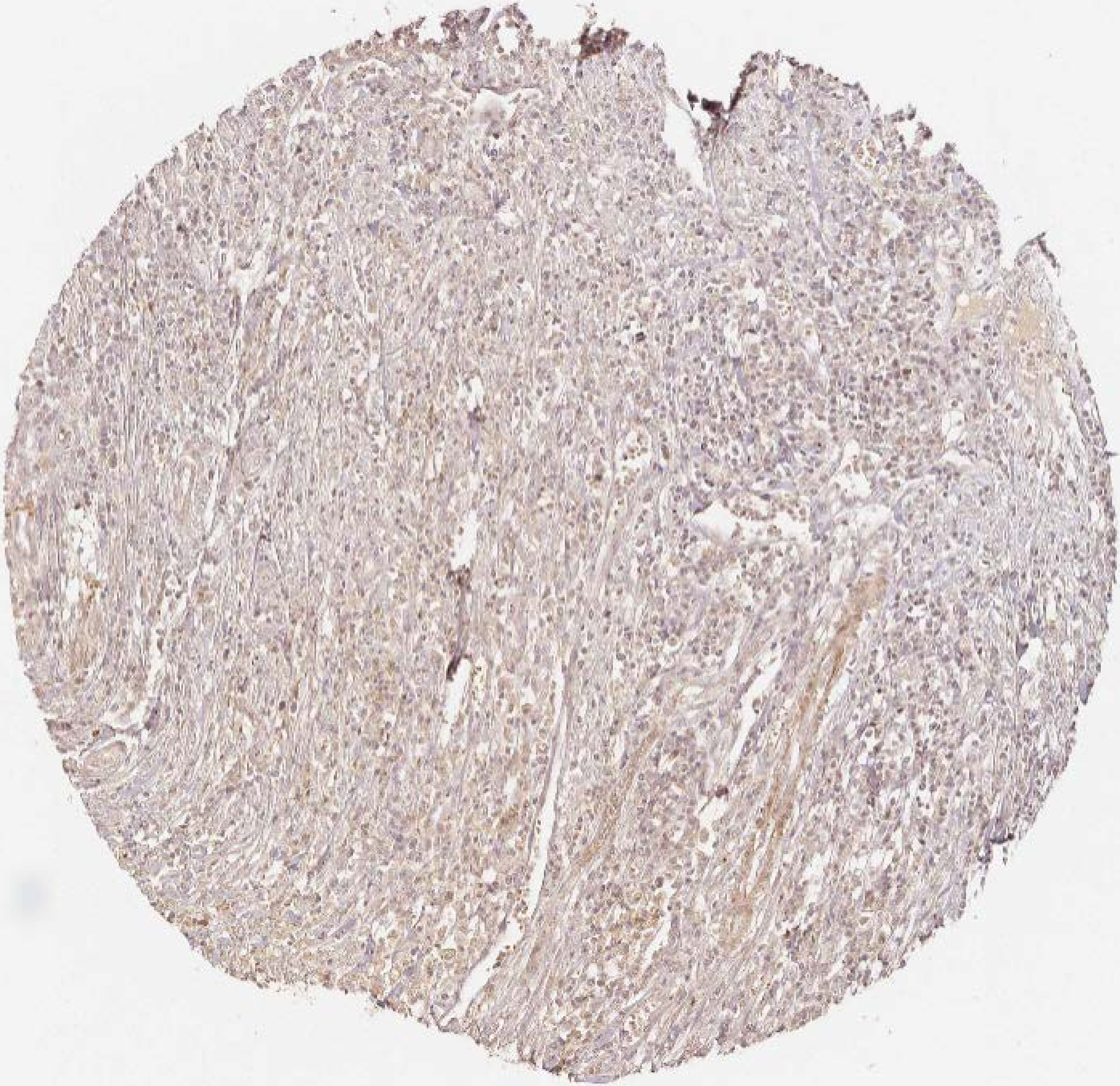

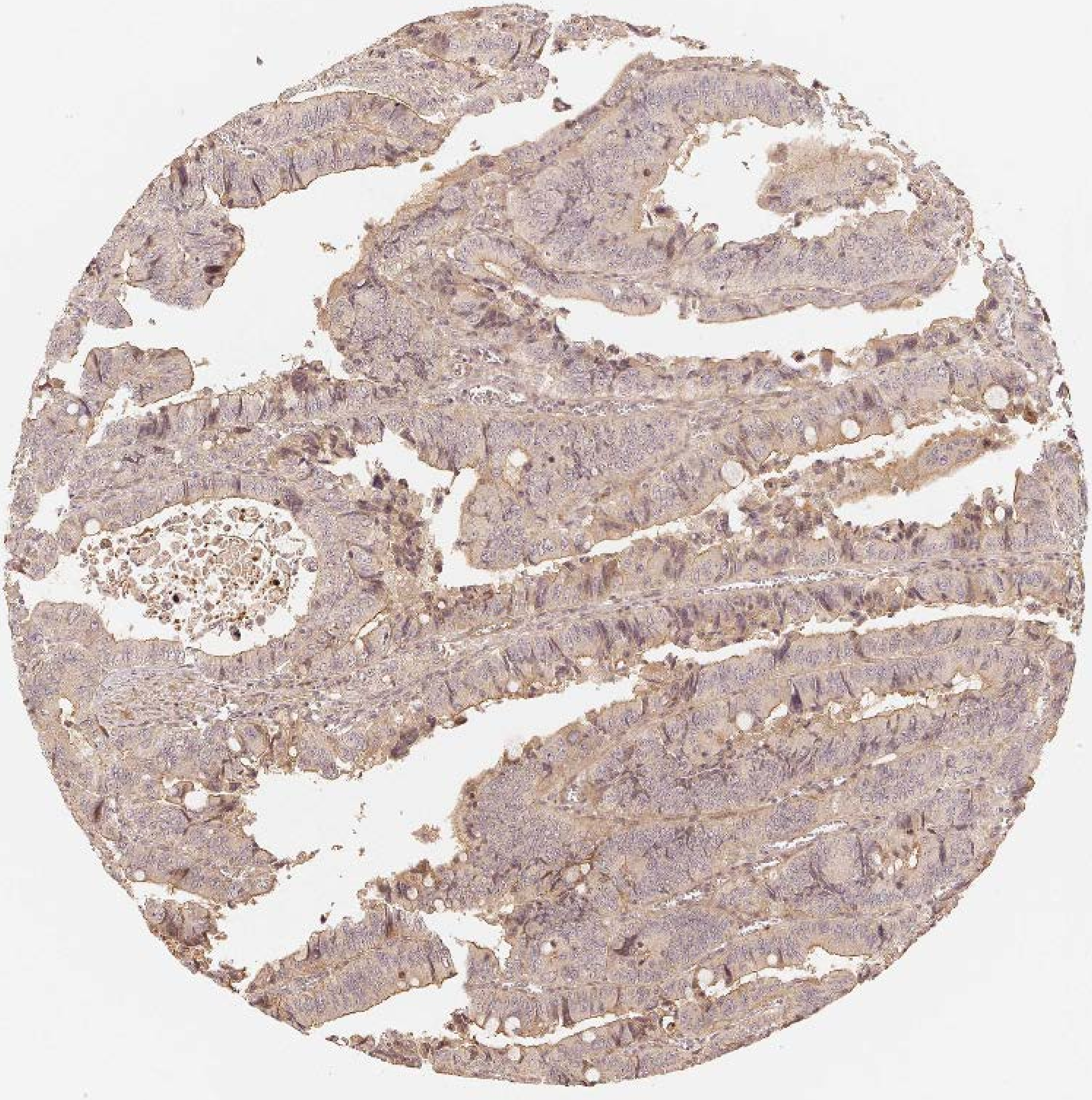

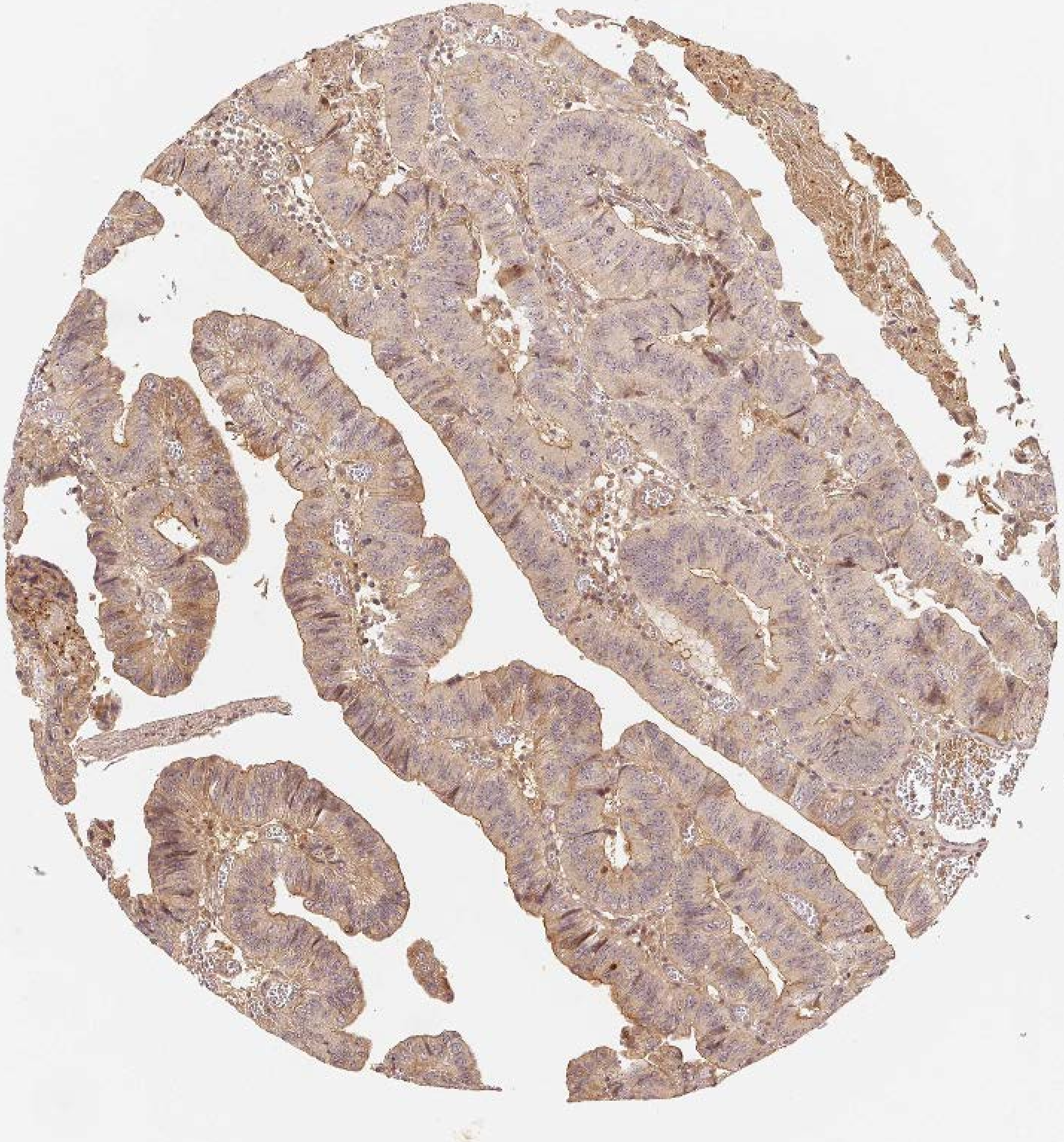

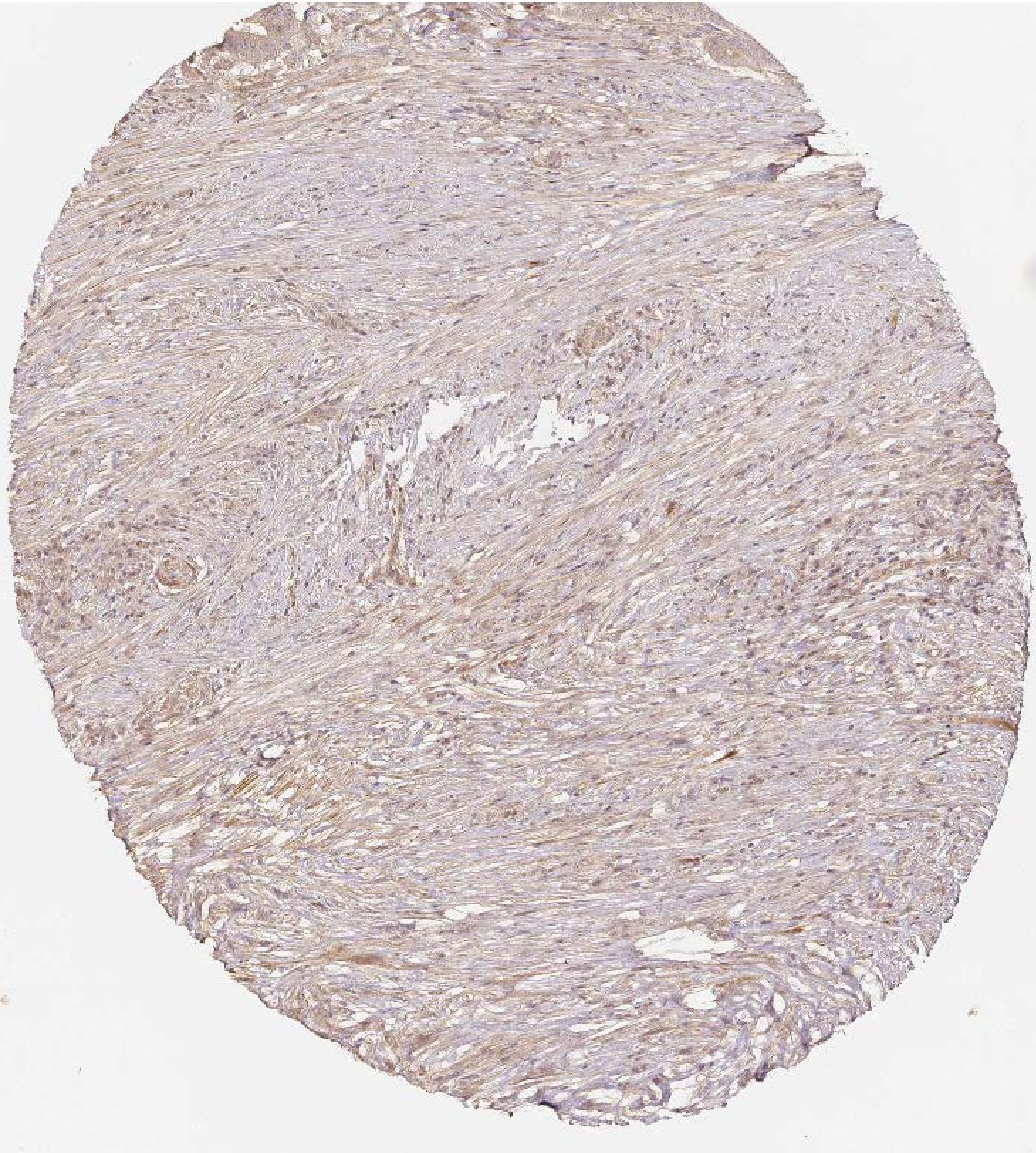

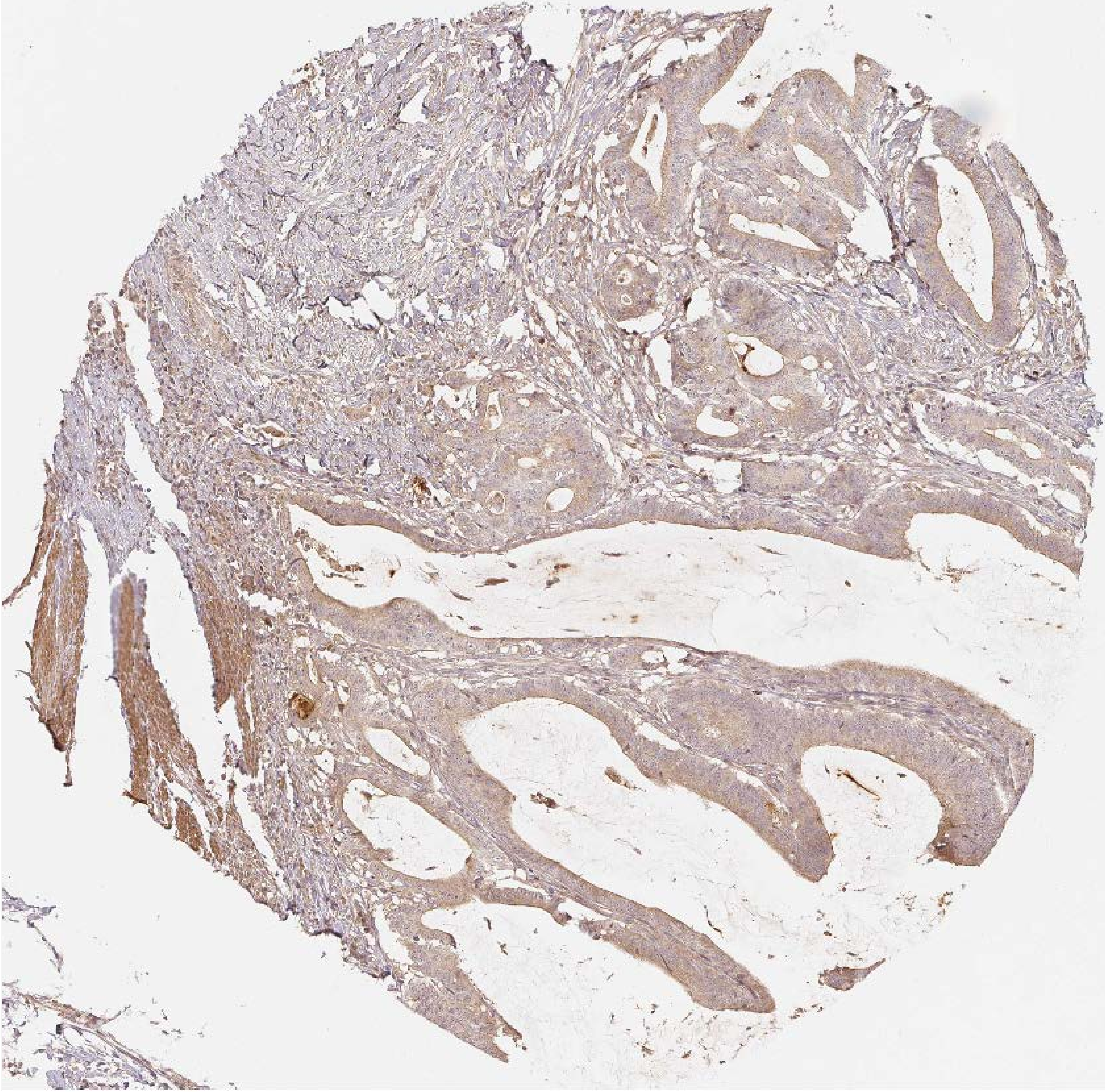

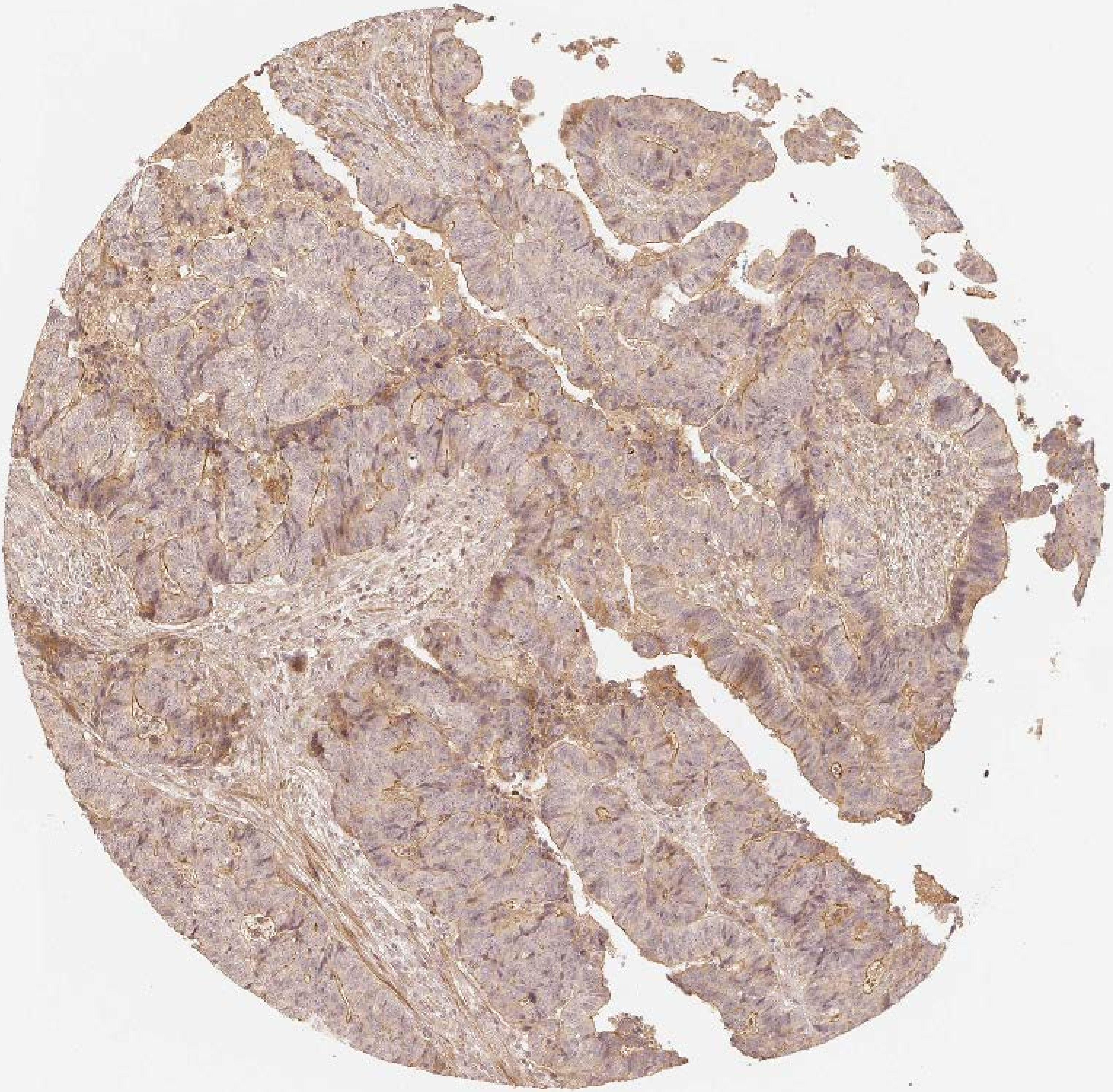

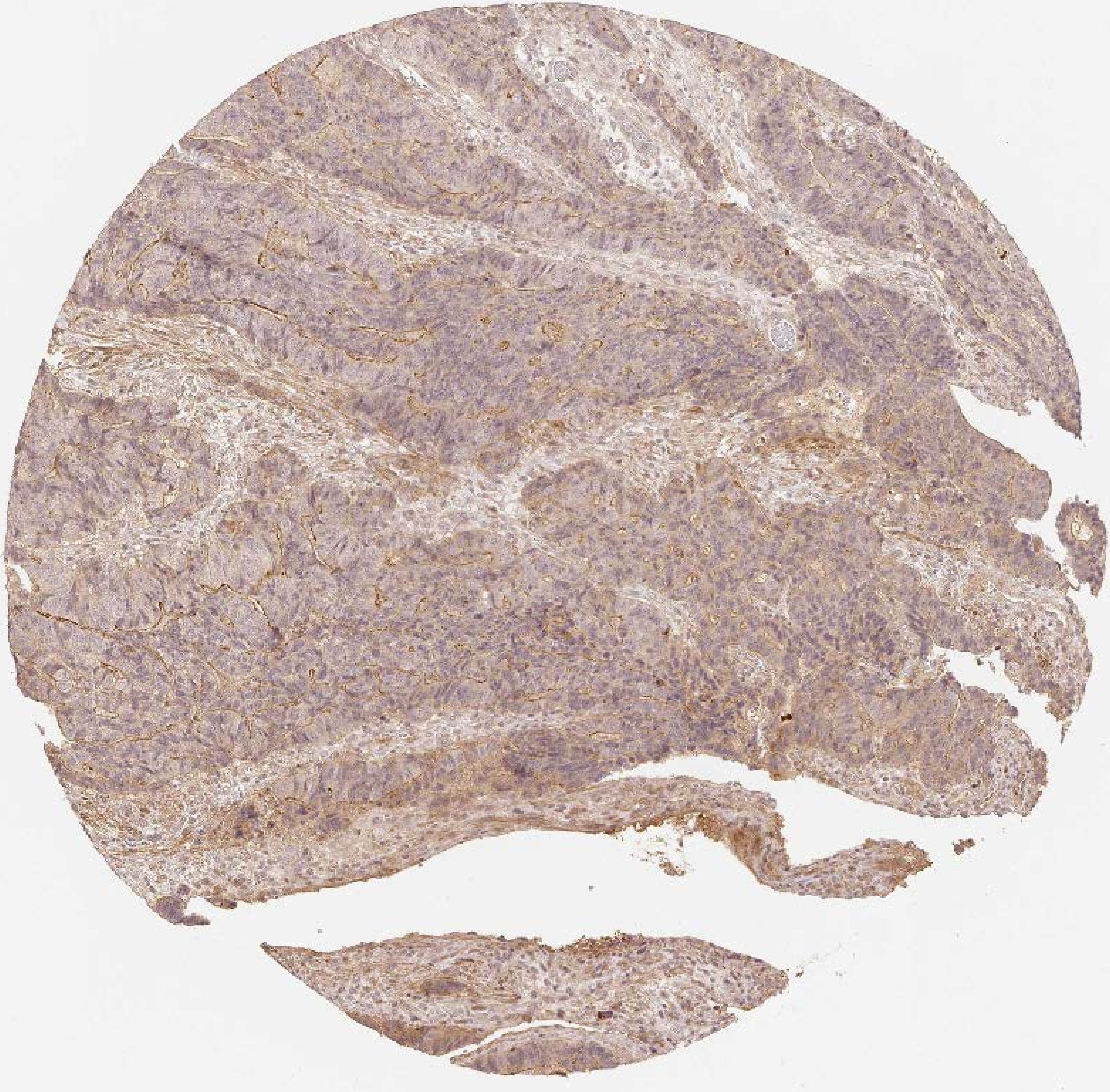

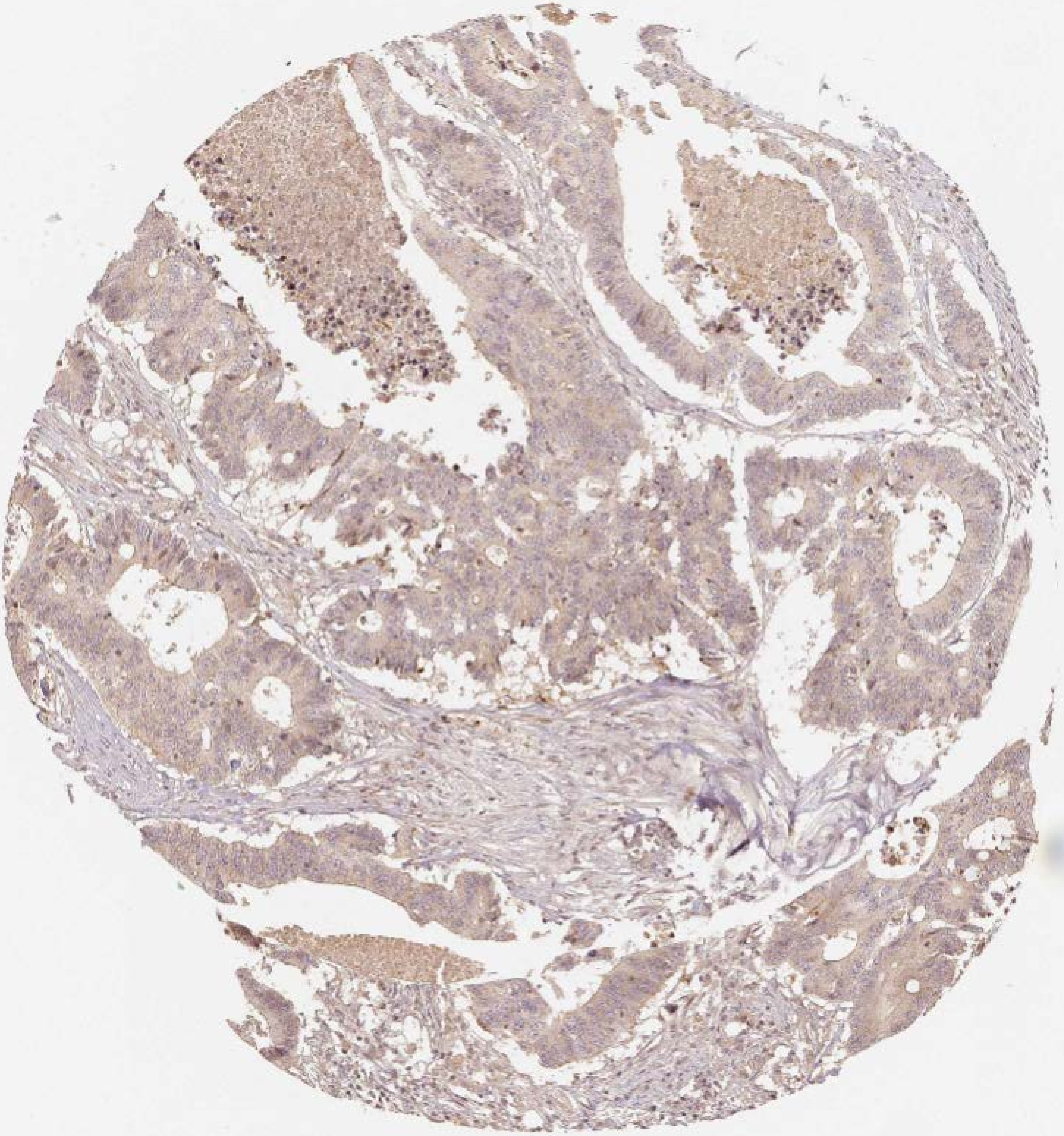

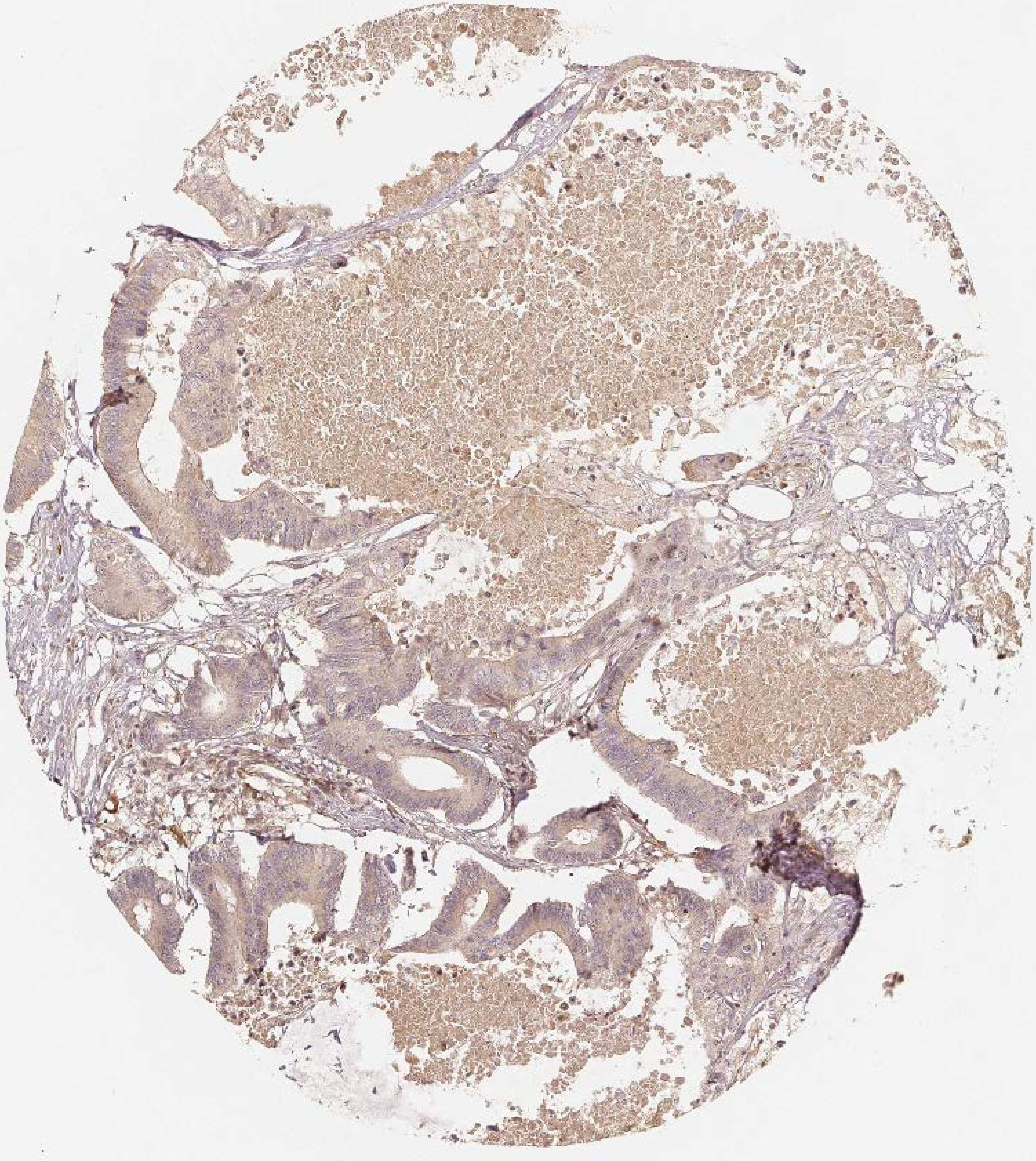

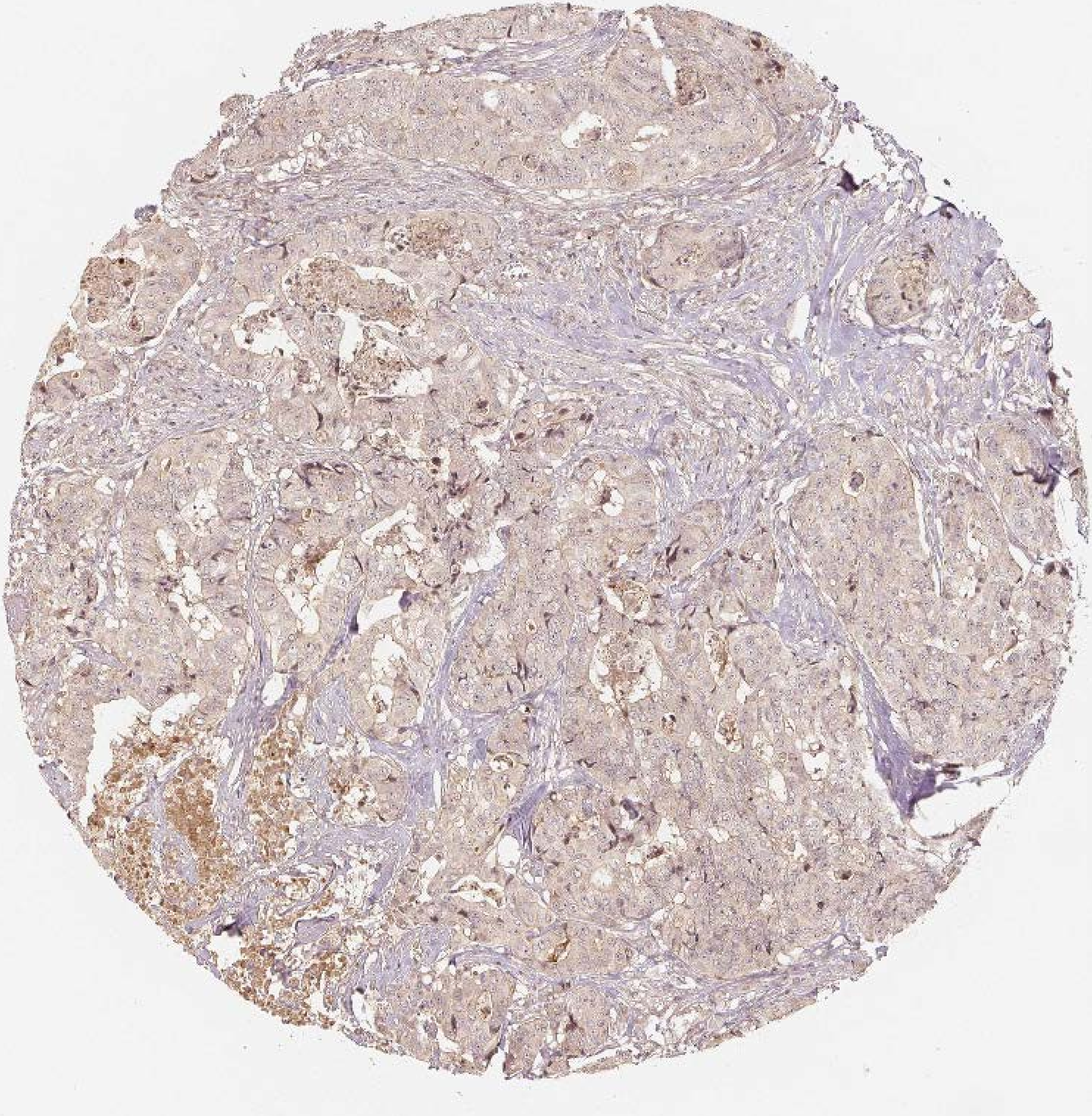

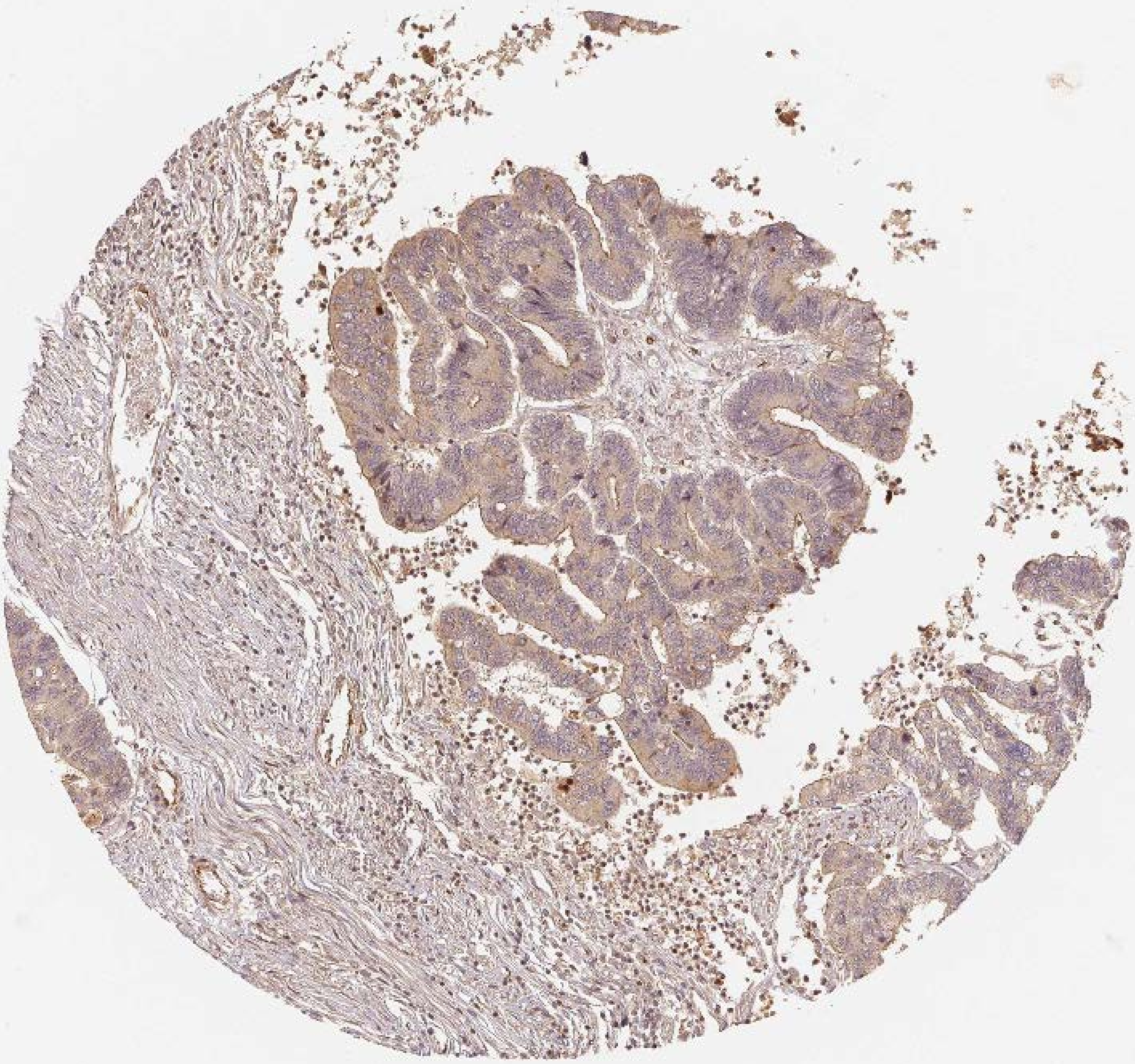

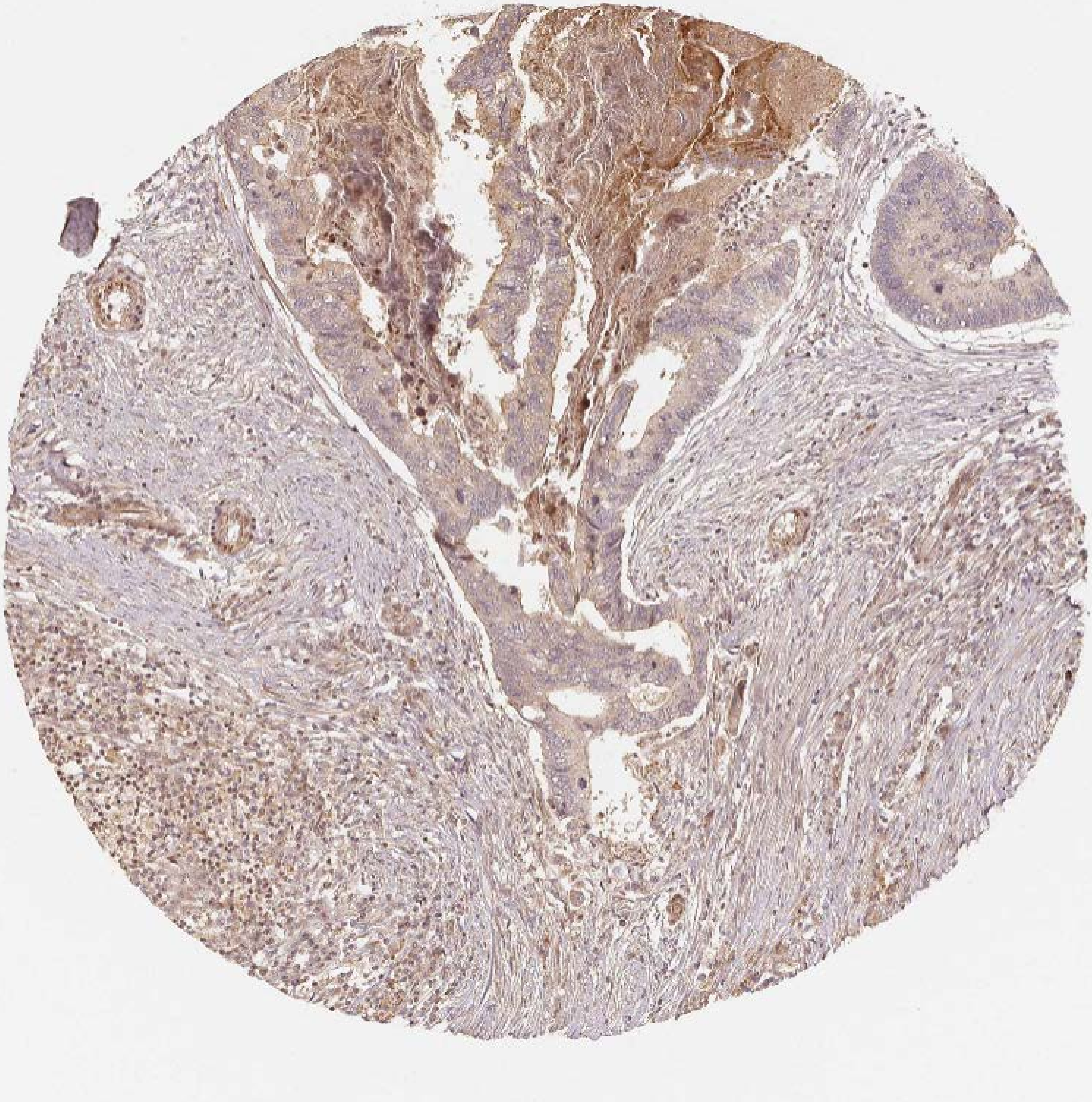

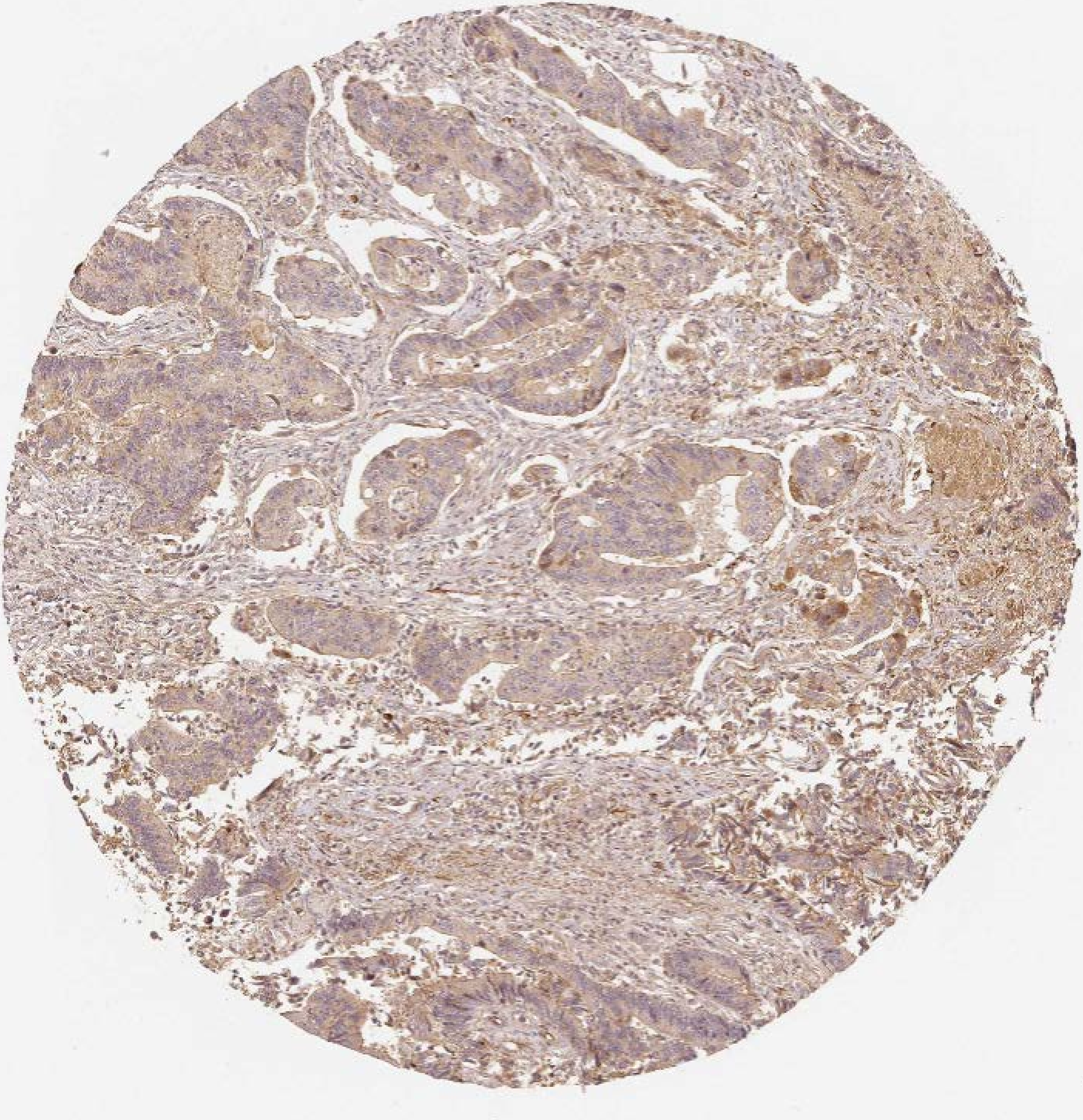

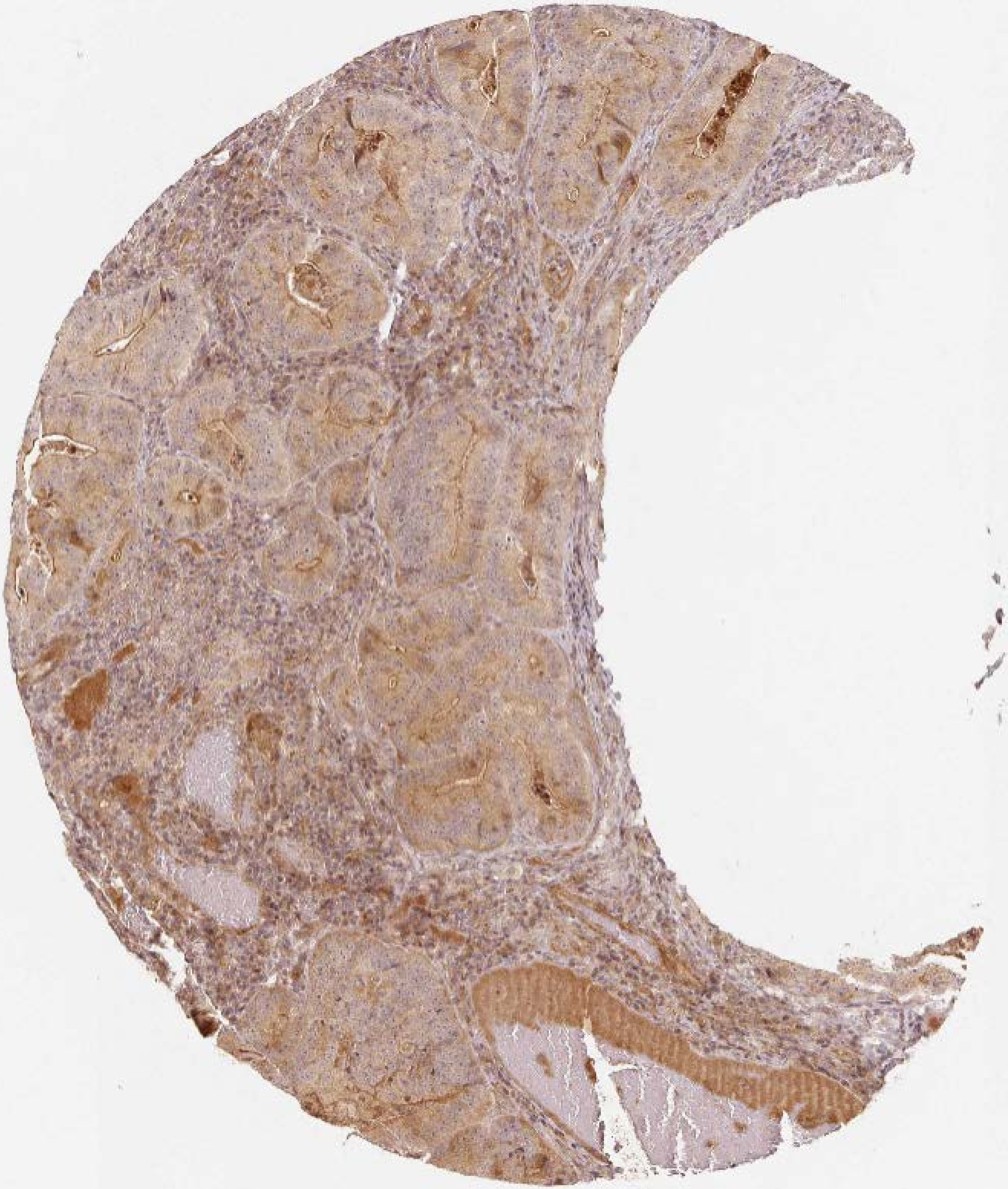

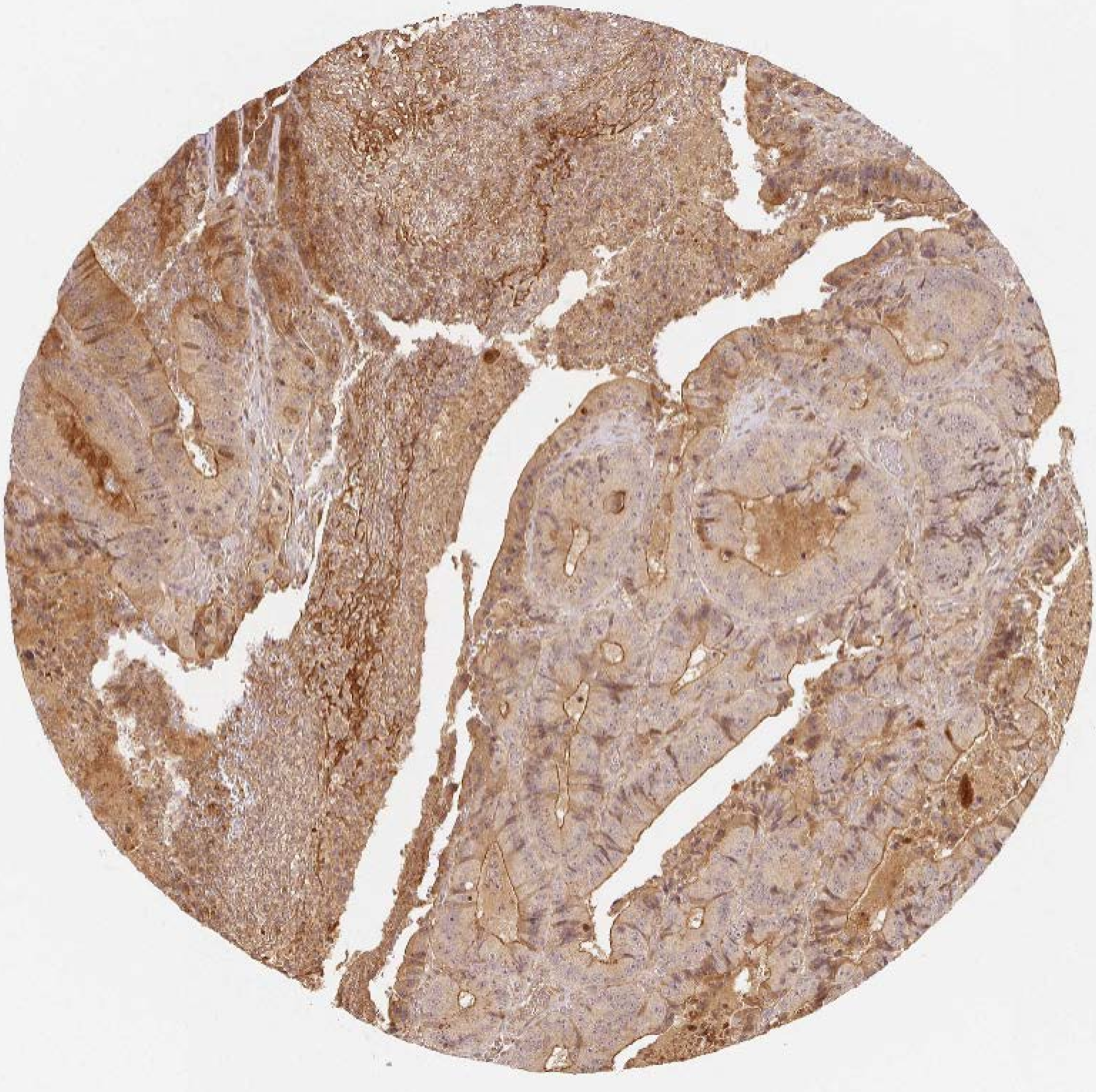

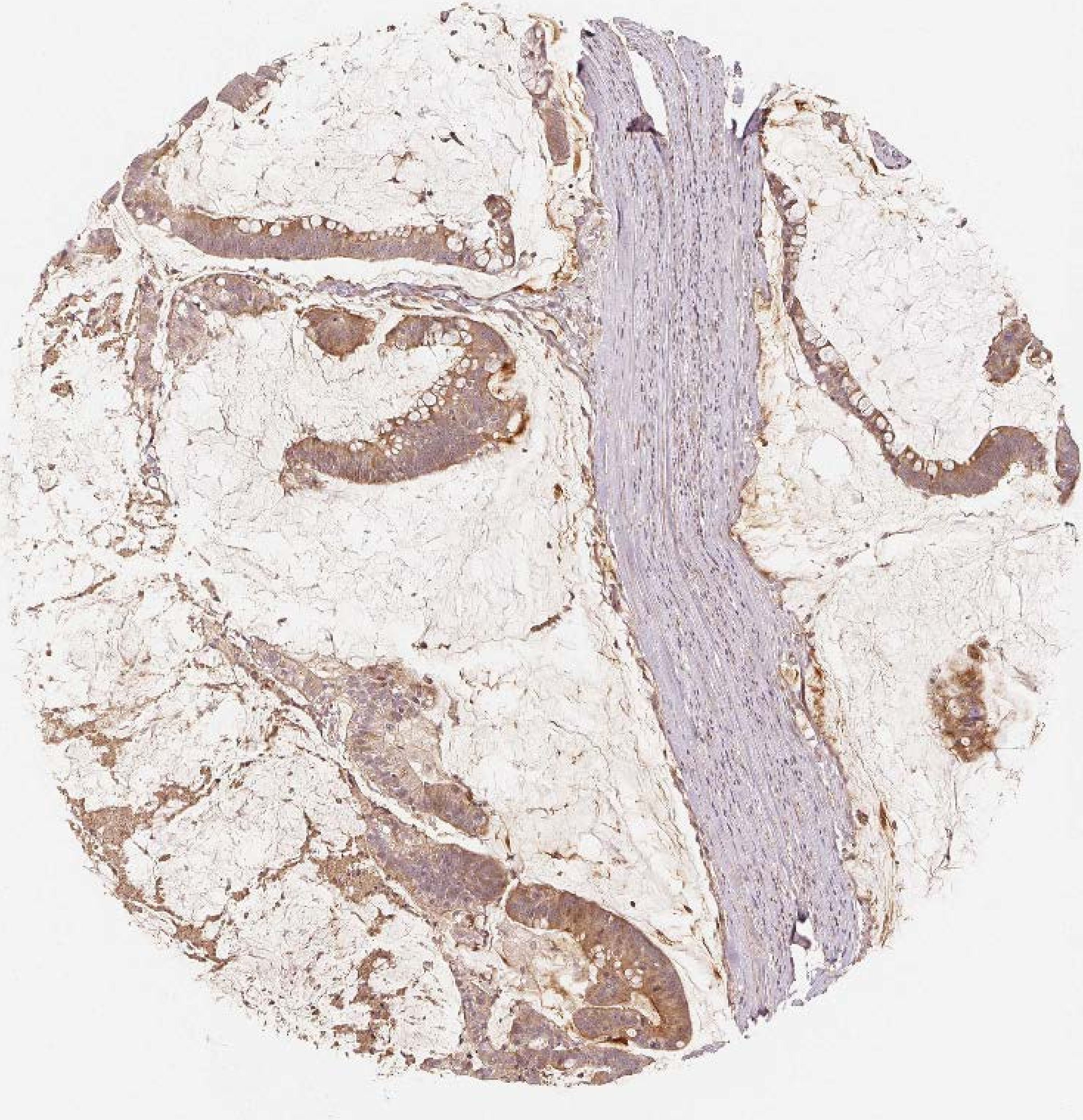

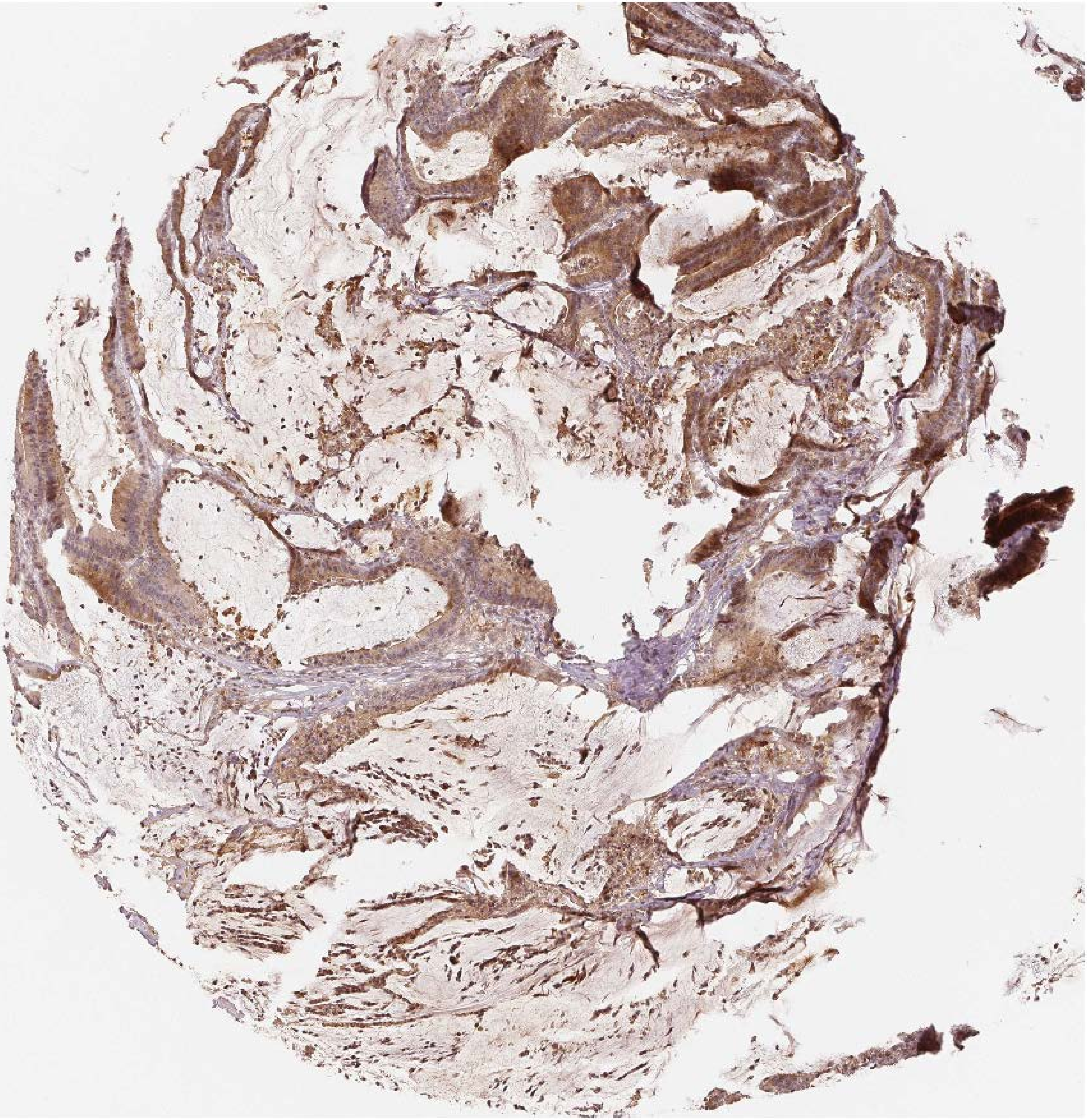

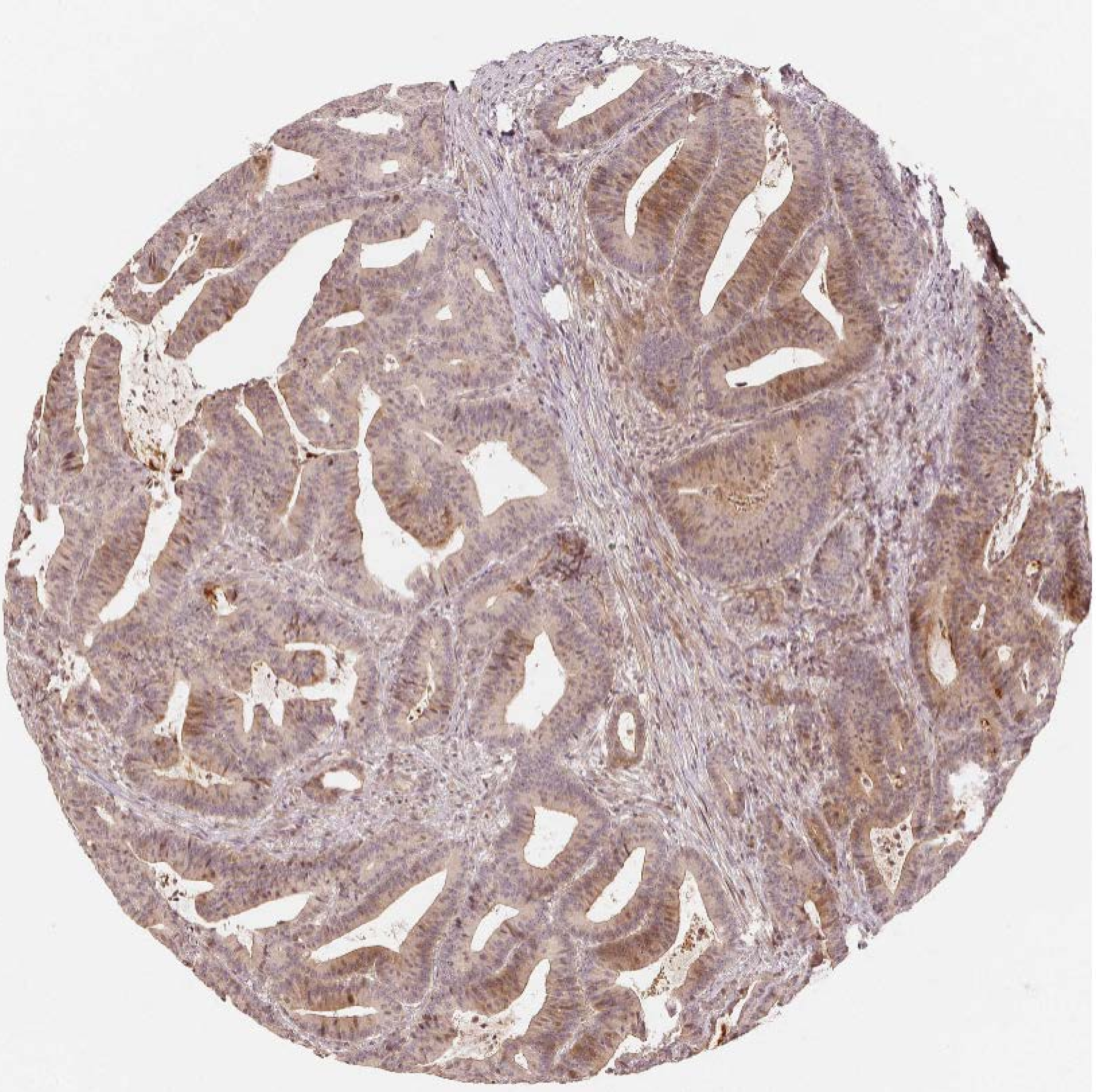

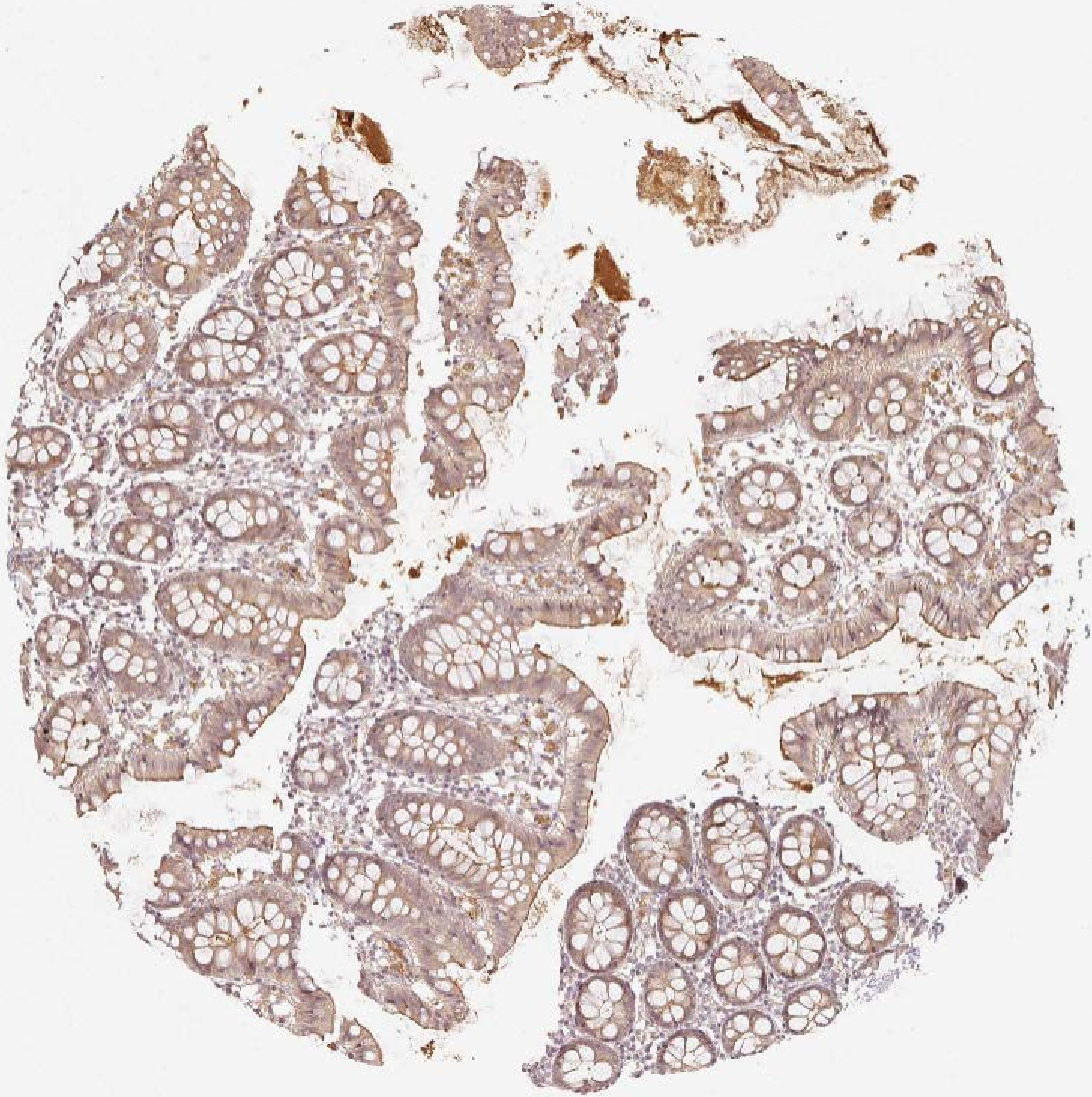

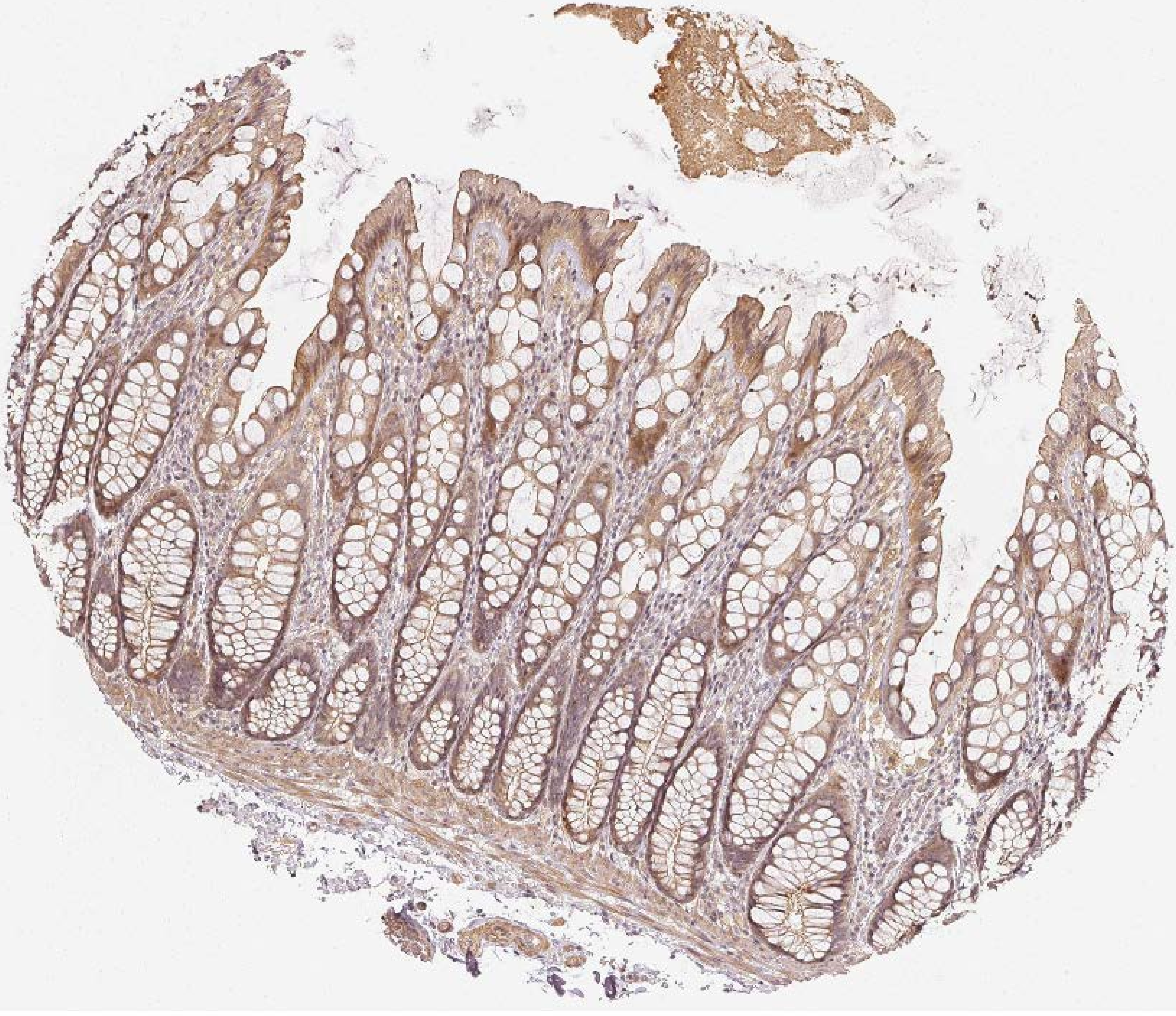

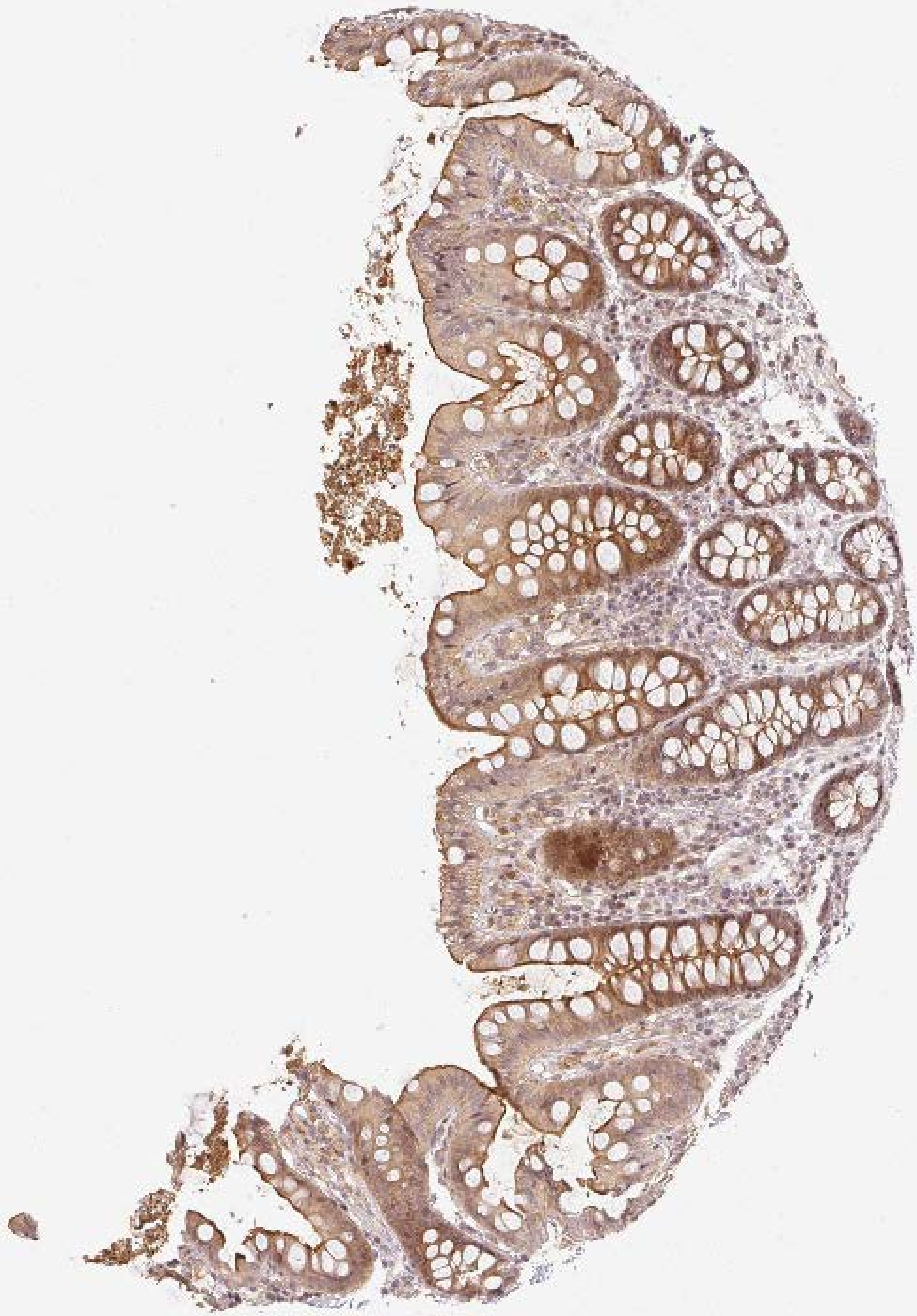

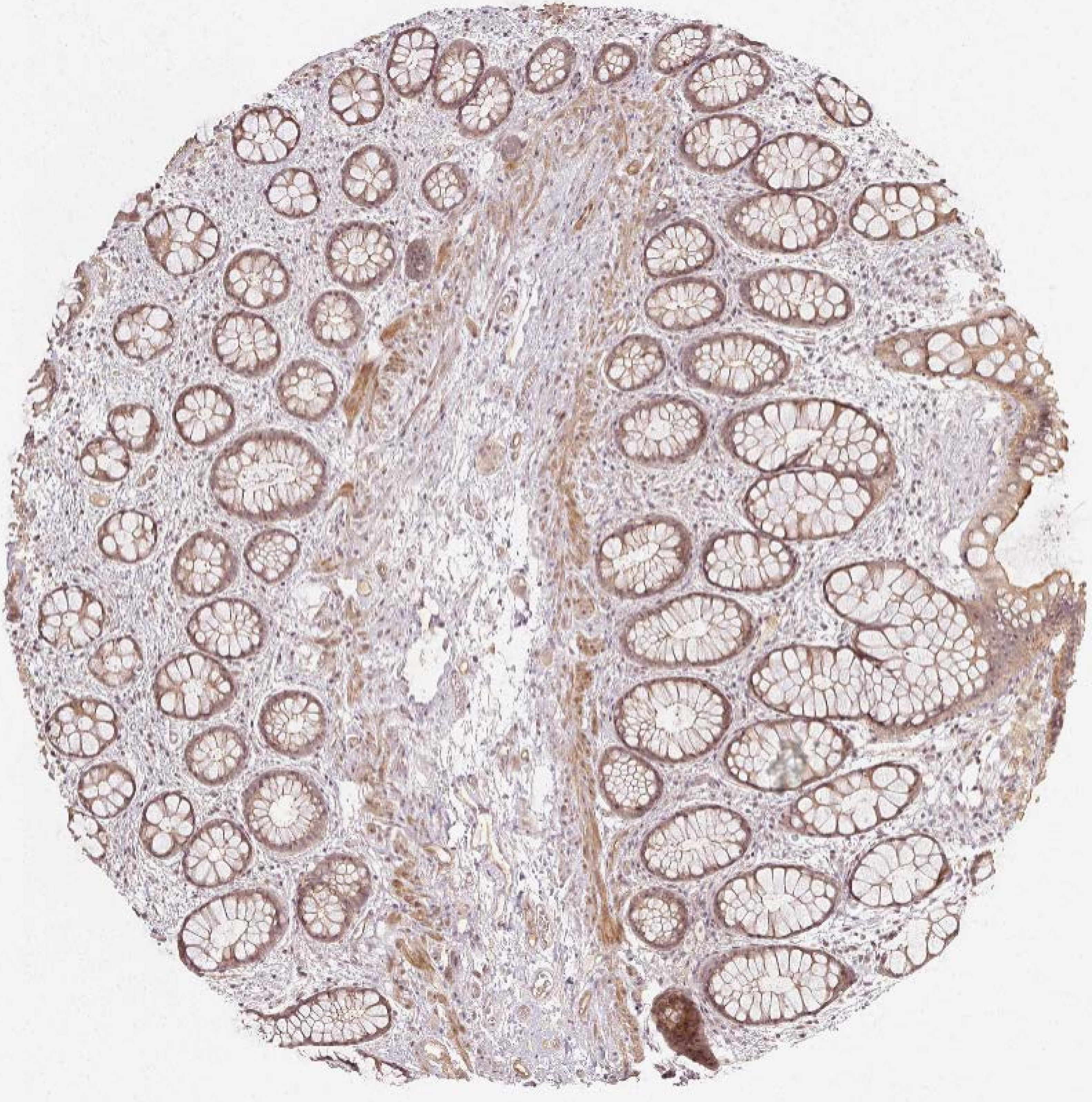

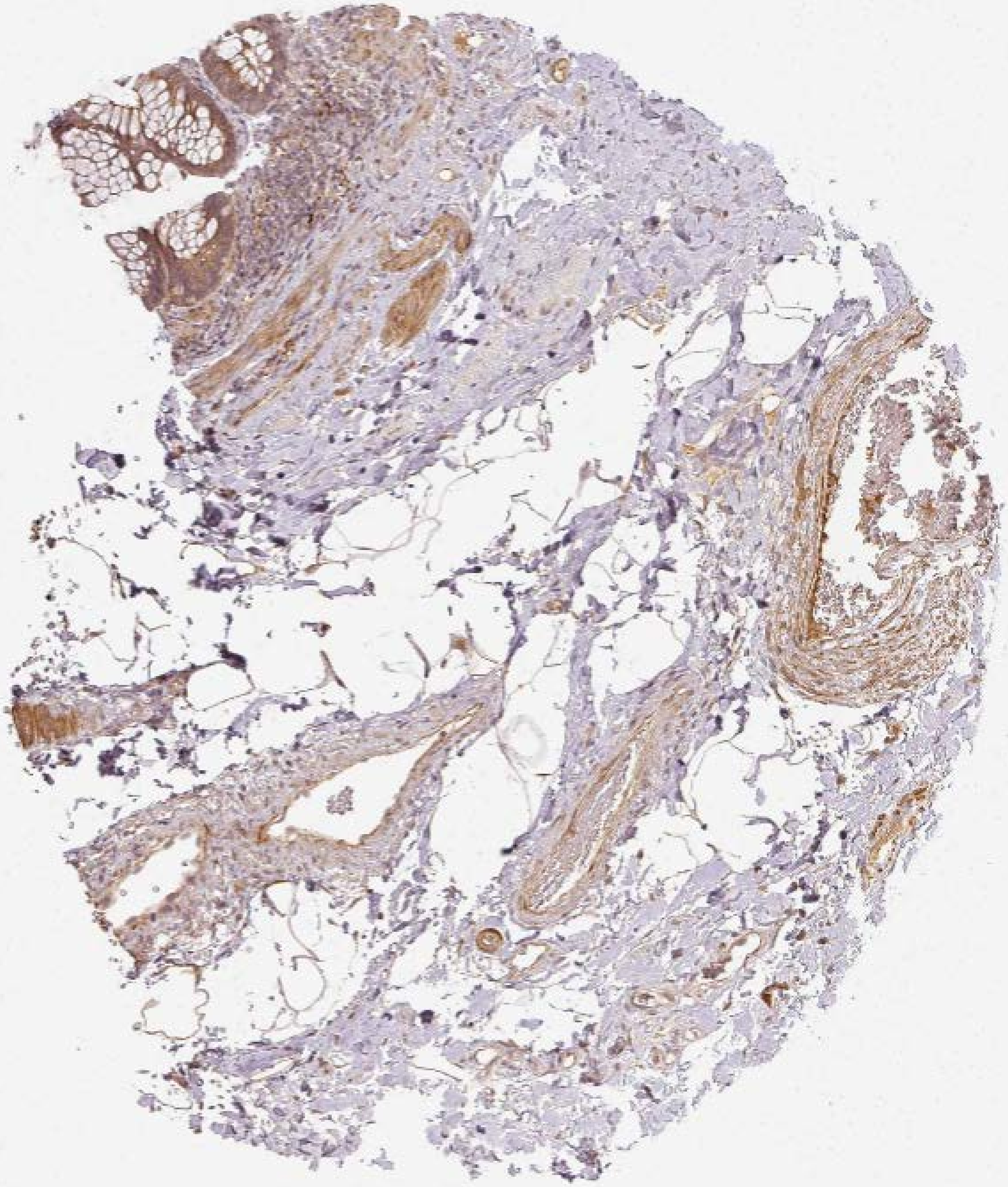

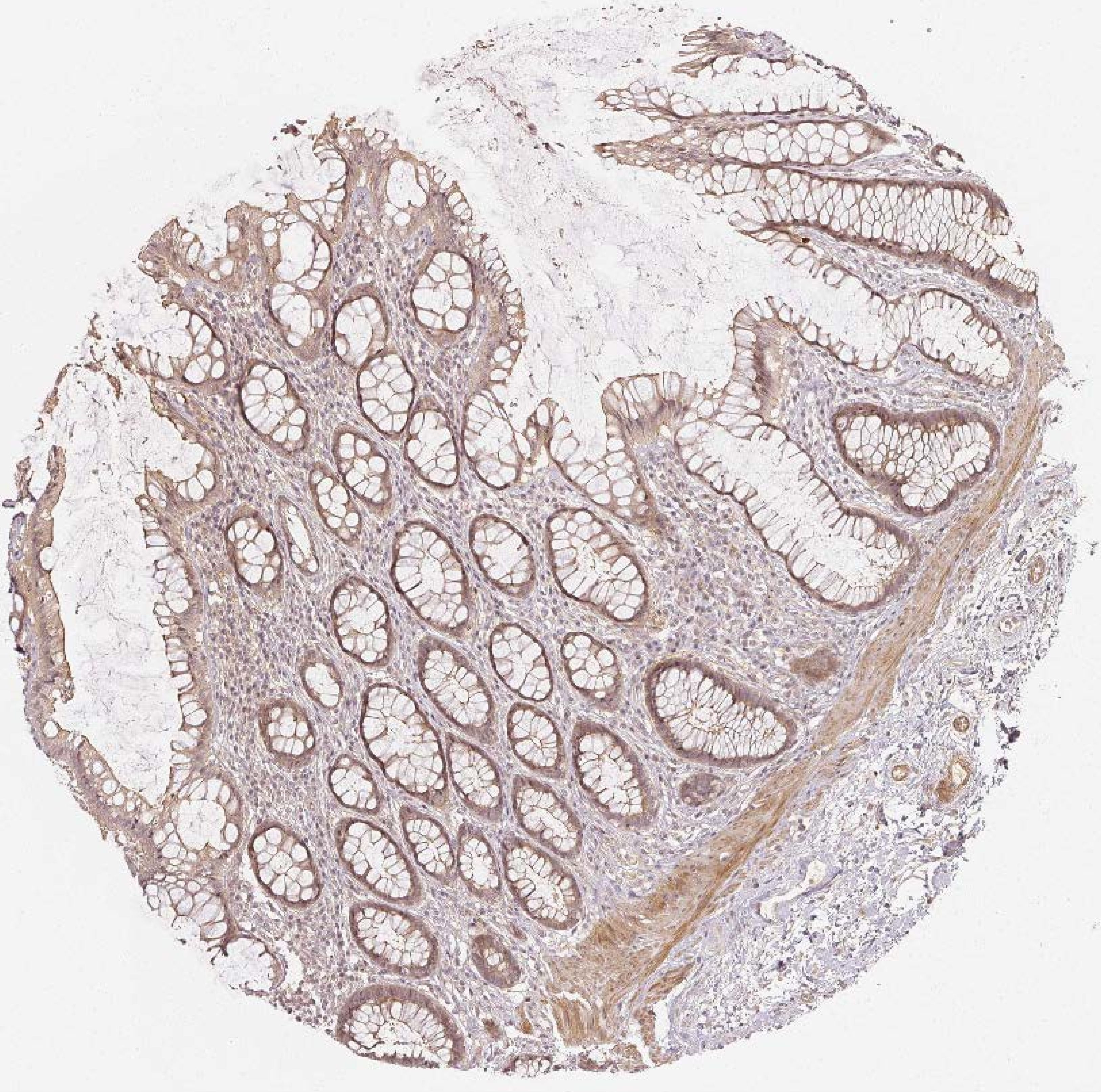

Supplement: Supplementary file 2 — Supplementary figure 2. [file jcav14p1956s2.pdf]
