# Supplementary material for: Network pharmacology and bioinformatics were used to construct a prognostic model and immunoassay of core target genes in the combination of quercetin and kaempferol in the treatment of colorectal cancer
Source: J Cancer. 2023 Jul 3;14(11):1956–80. doi: 10.7150/jca.85517 (PMC10367918; doi:10.7150/jca.85517)

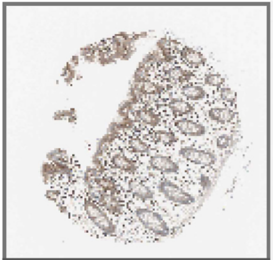

Colon

HPA007875

Male, age 14
Colon (T-67000)
Normal tissue, NOS (M-00100)
Patient id: 1990

Endothelial cells	
Staining:	**Not detected**
Intensity:	**Negative**
Quantity:	**None**

Glandular cells	
Staining:	**High**
Intensity:	**Strong**
Quantity:	**75%-25%**
Location:	**Cytoplasmic/ membranous**

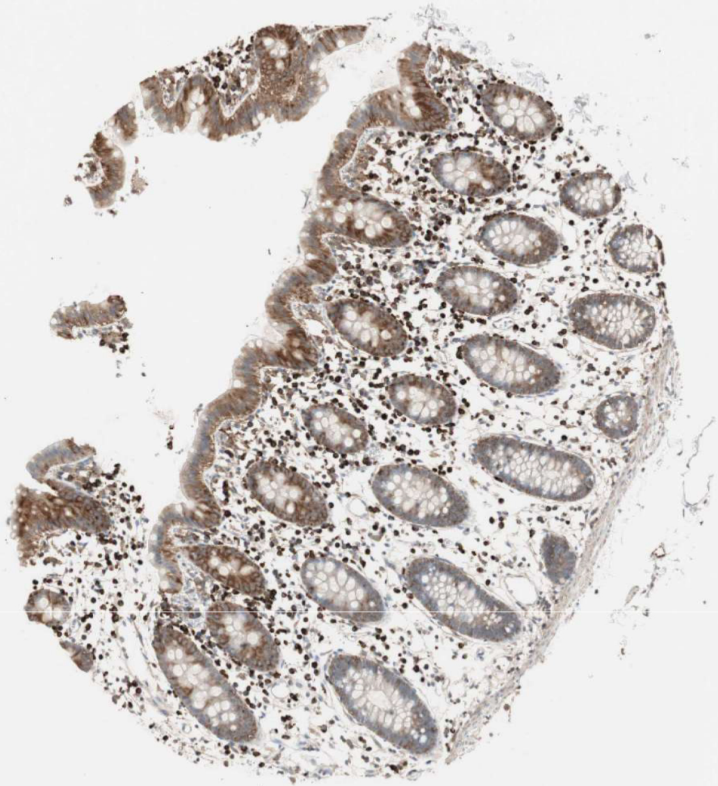

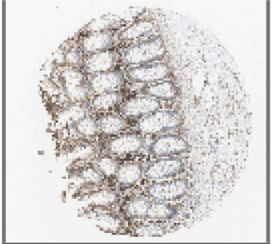

Rectum

HPA007875

Female, age 46
Rectum (T-68000)
Normal tissue, NOS (M-00100)
Patient id: 1876

Glandular cells

Staining: **High**

Intensity: **Strong**
Quantity: **75%-25%**
Location: **Cytoplasmic/ membranous**

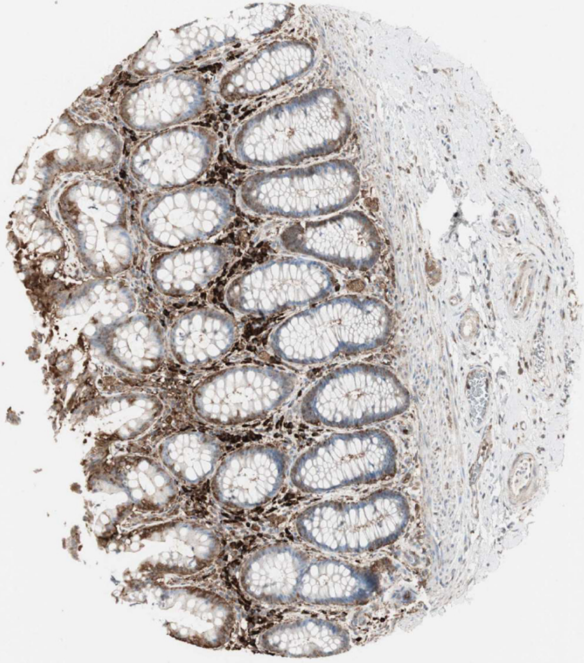

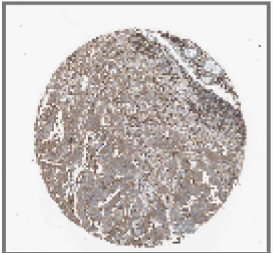

Colorectal cancer

HPA007875

Male, age 45
Colon (T-67000)
Adenocarcinoma, NOS
(M-81403)
Patient id: 693

Tumor cells

Staining: **High**

Intensity: **Strong**
Quantity: **>75%**
Location: **Cytoplasmic/ membranous**

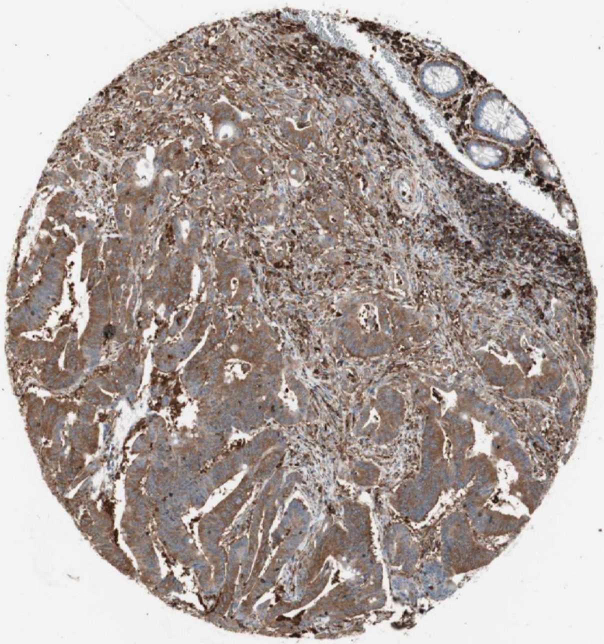

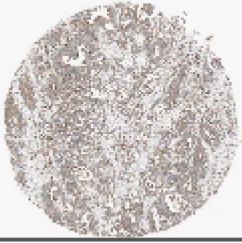

Colorectal cancer

HPA007875

Male, age 55
Rectum (T-68000)
Adenocarcinoma, NOS
(M-81403)
Patient id: 349

Tumor cells

Staining: **High**

Intensity: **Strong**
Quantity: **>75%**
Location: **Cytoplasmic/ membranous**

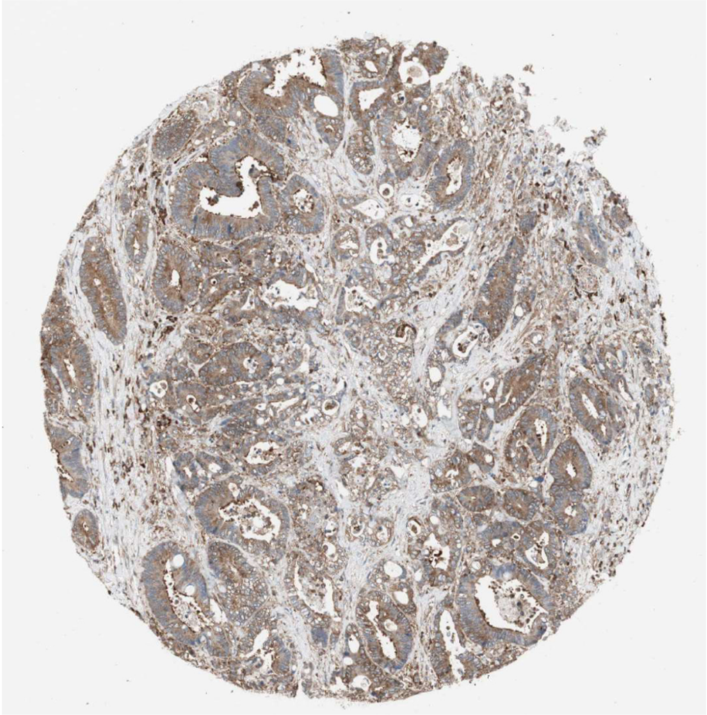

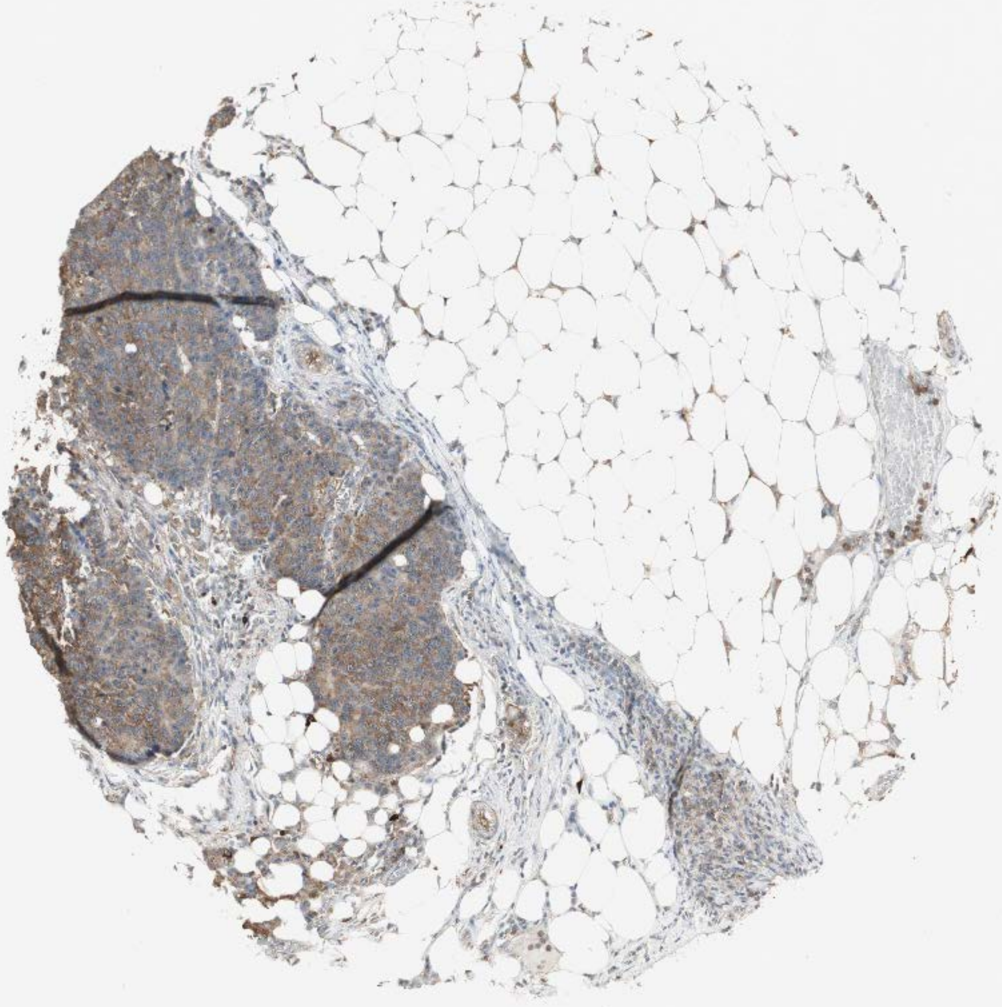

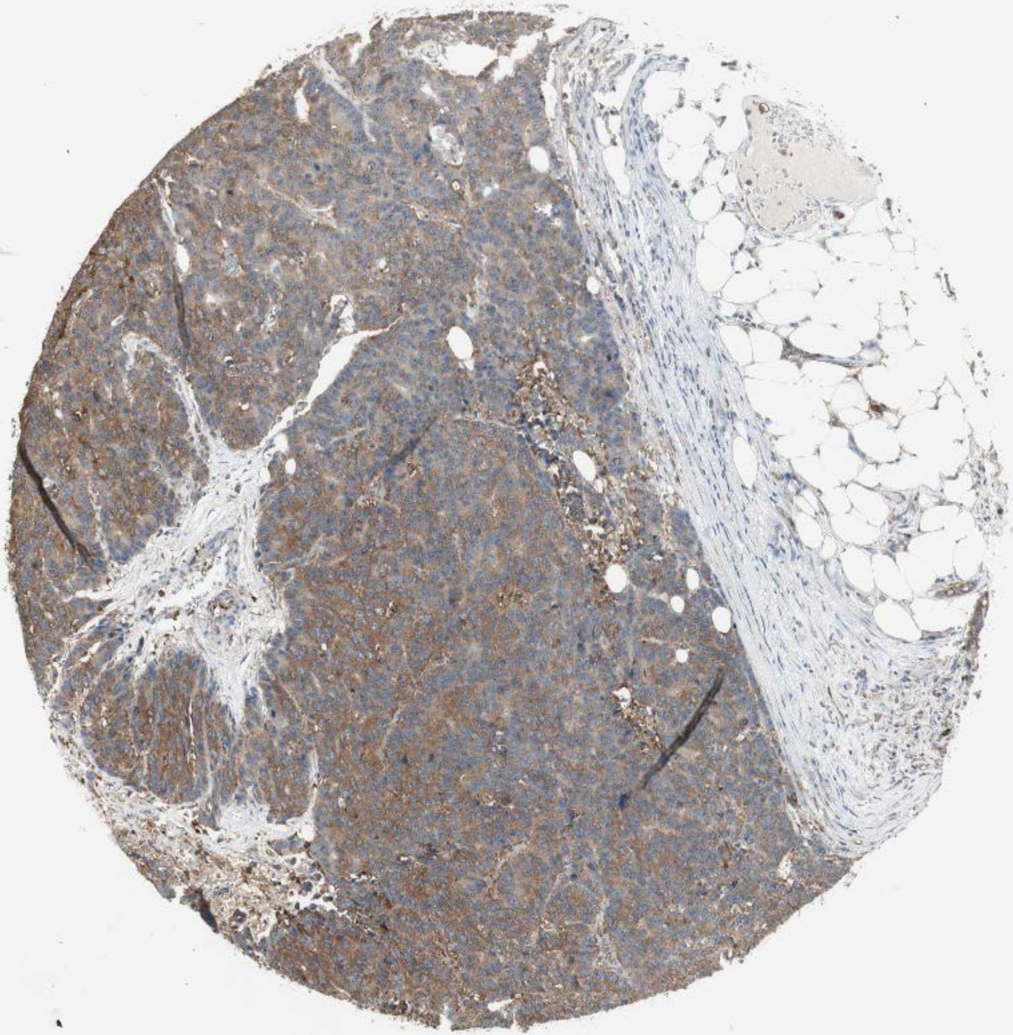

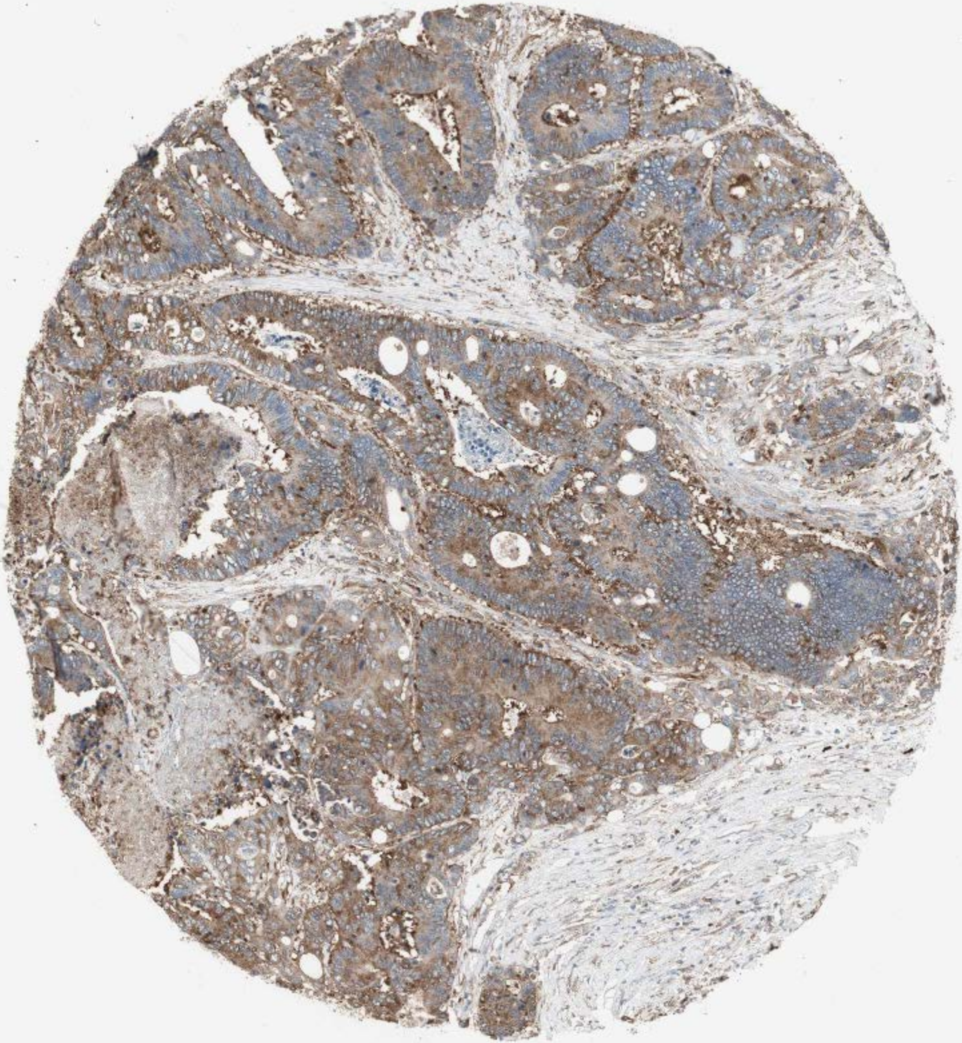

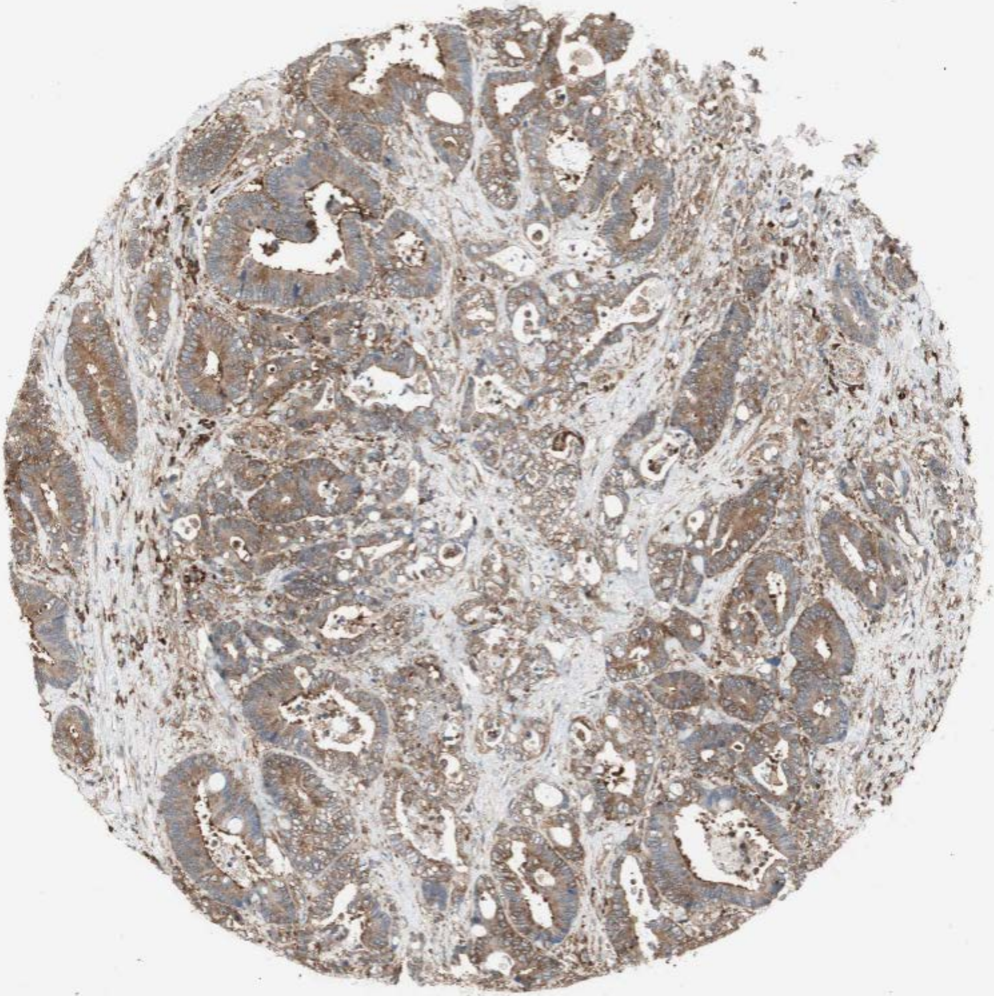

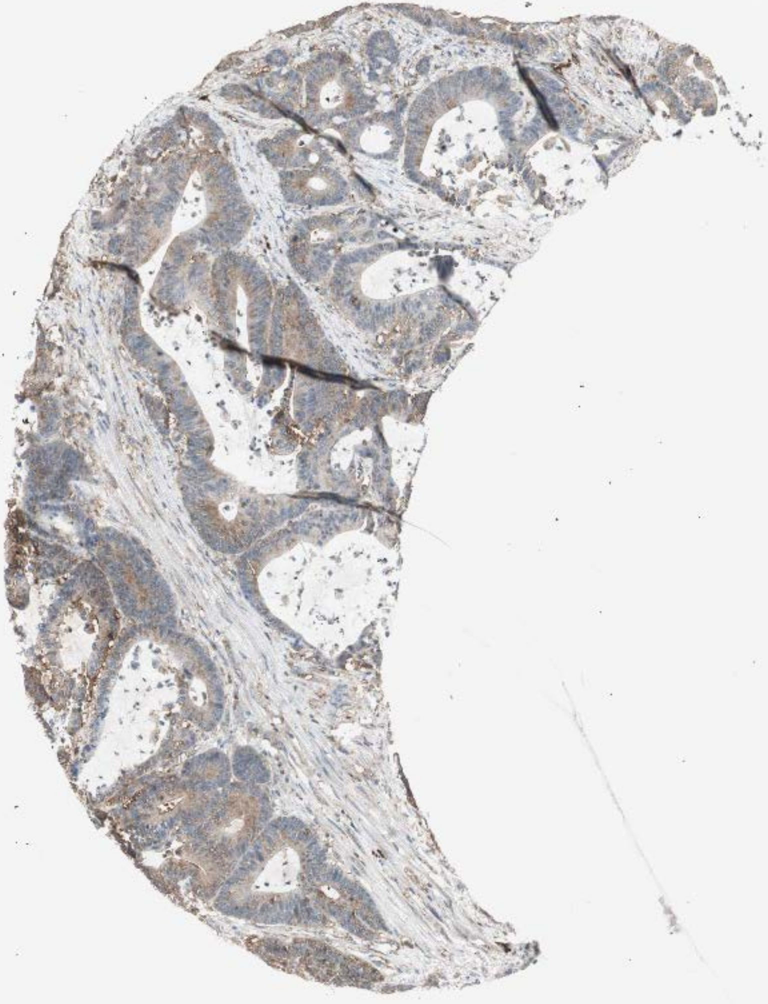

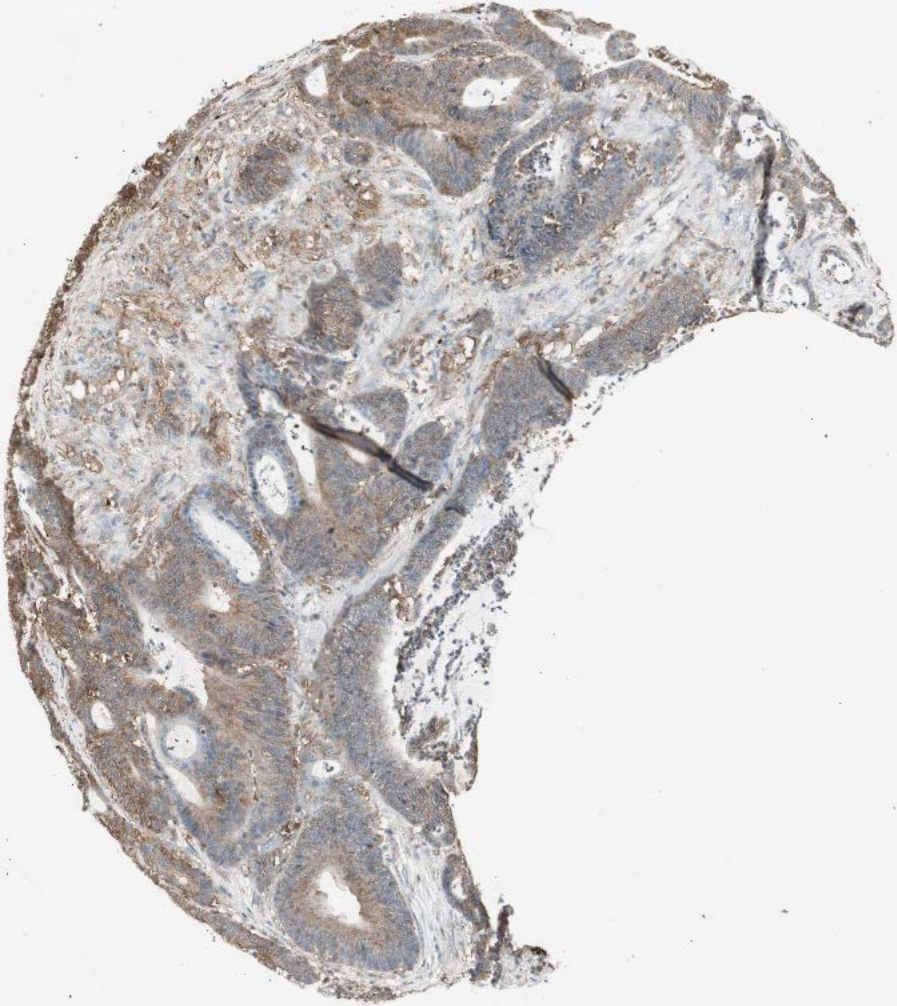

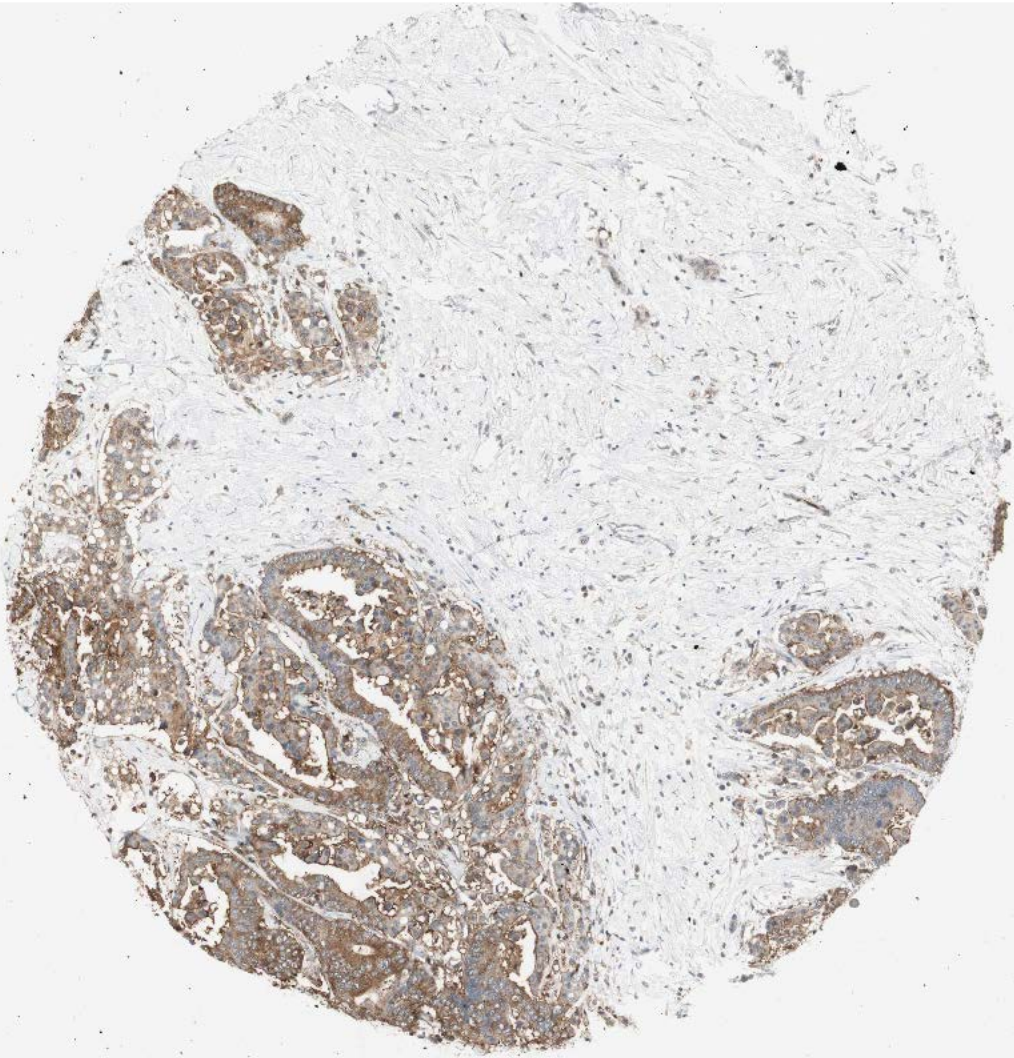

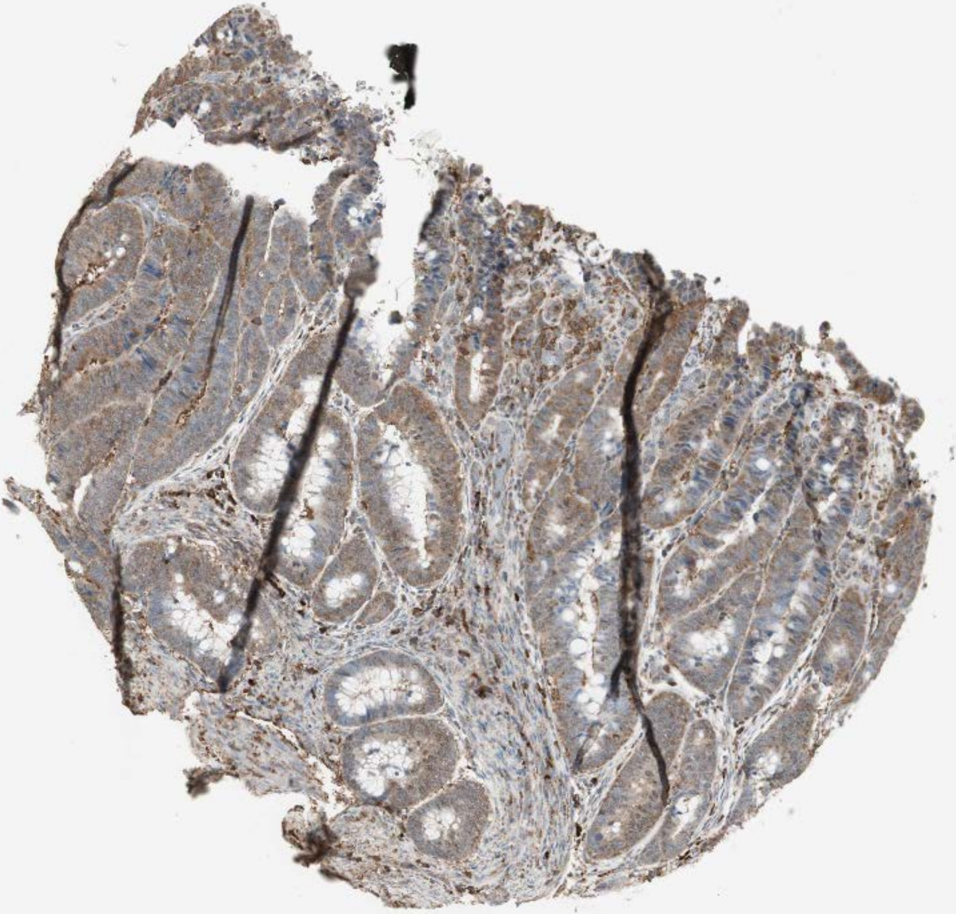

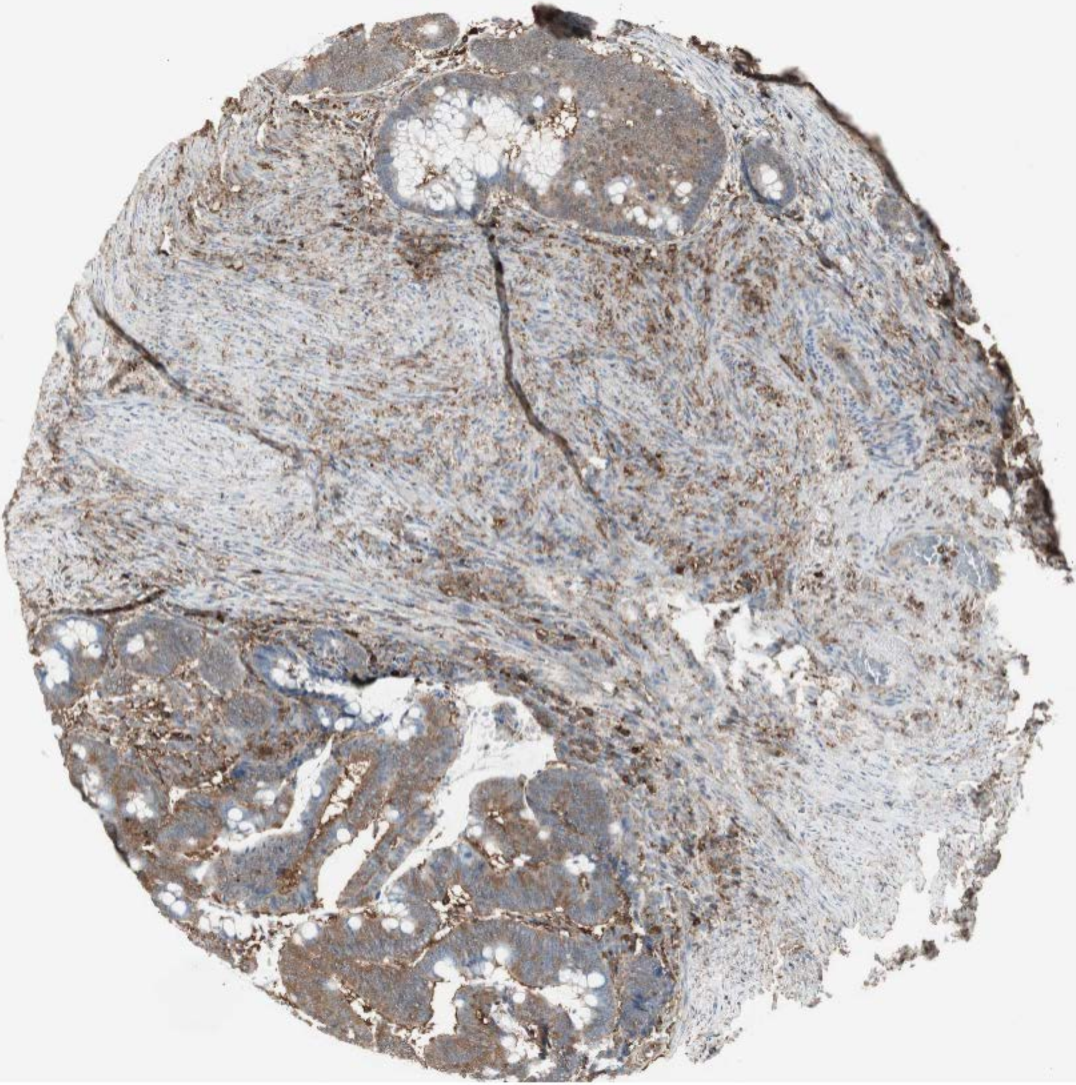

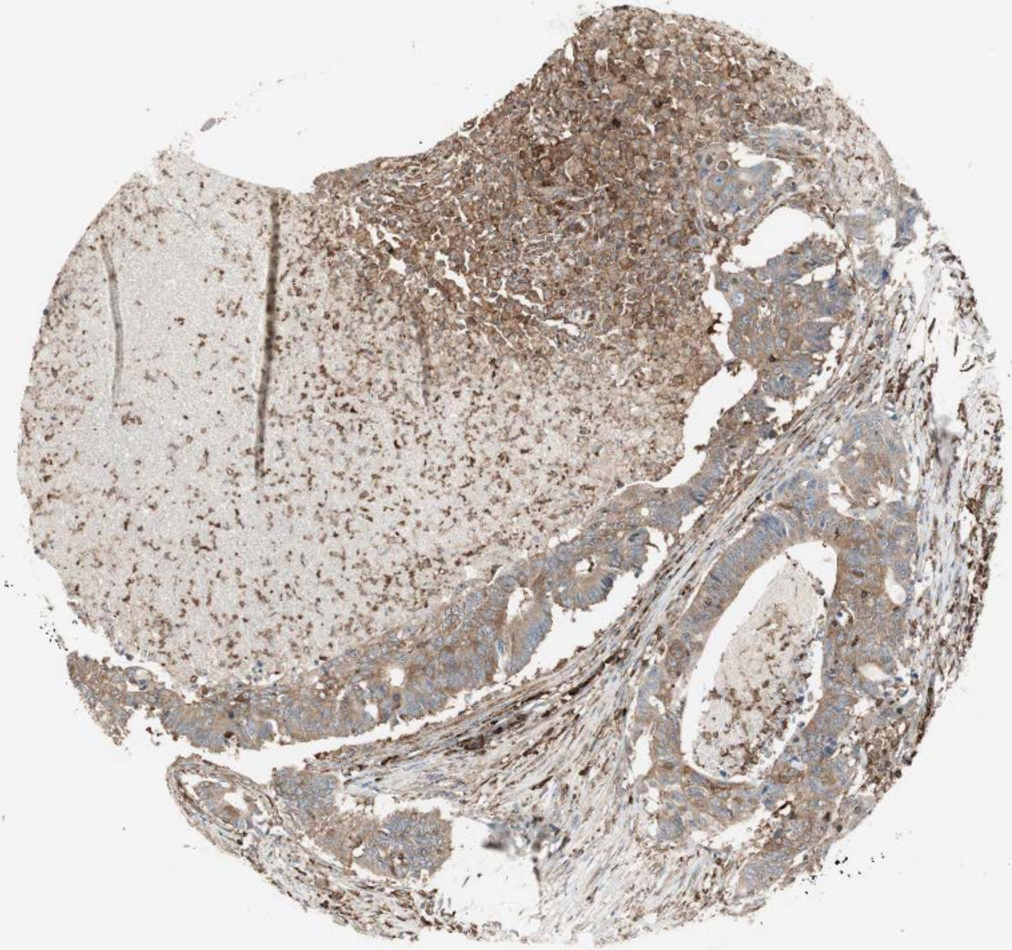

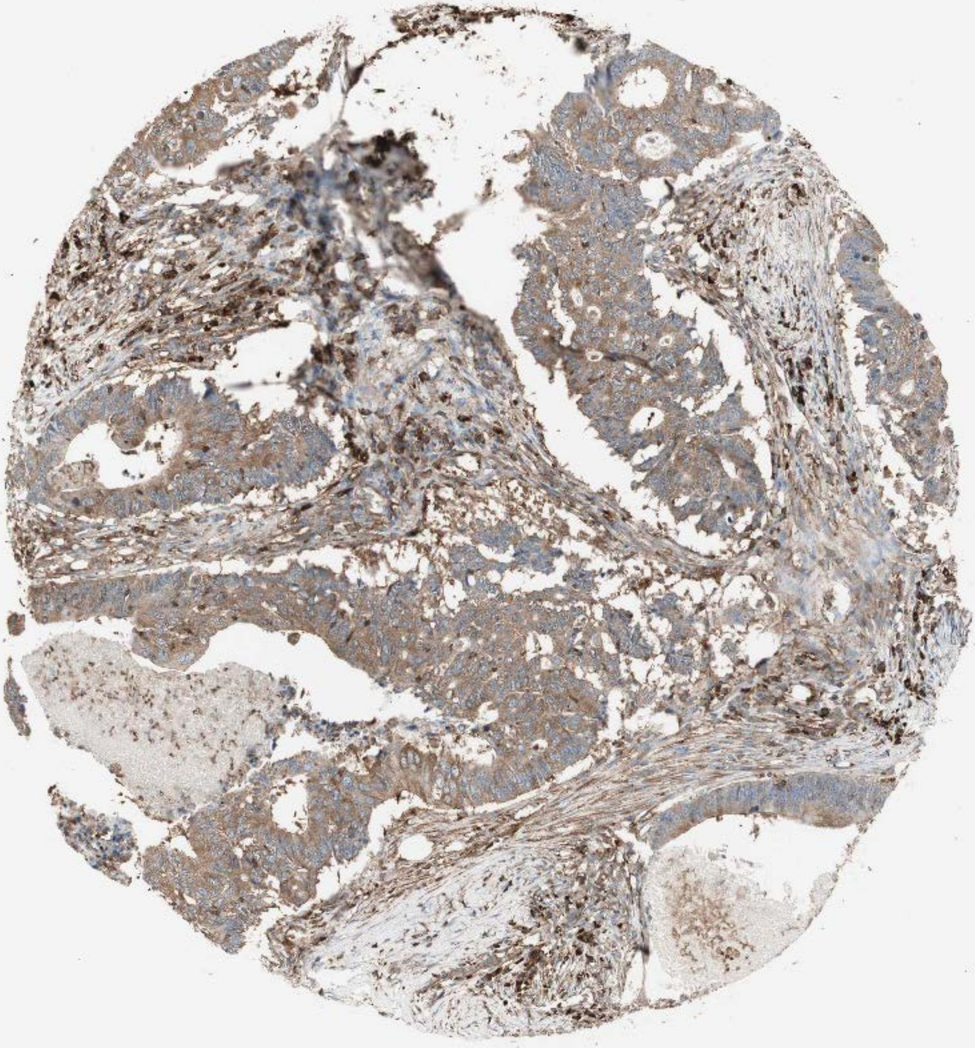

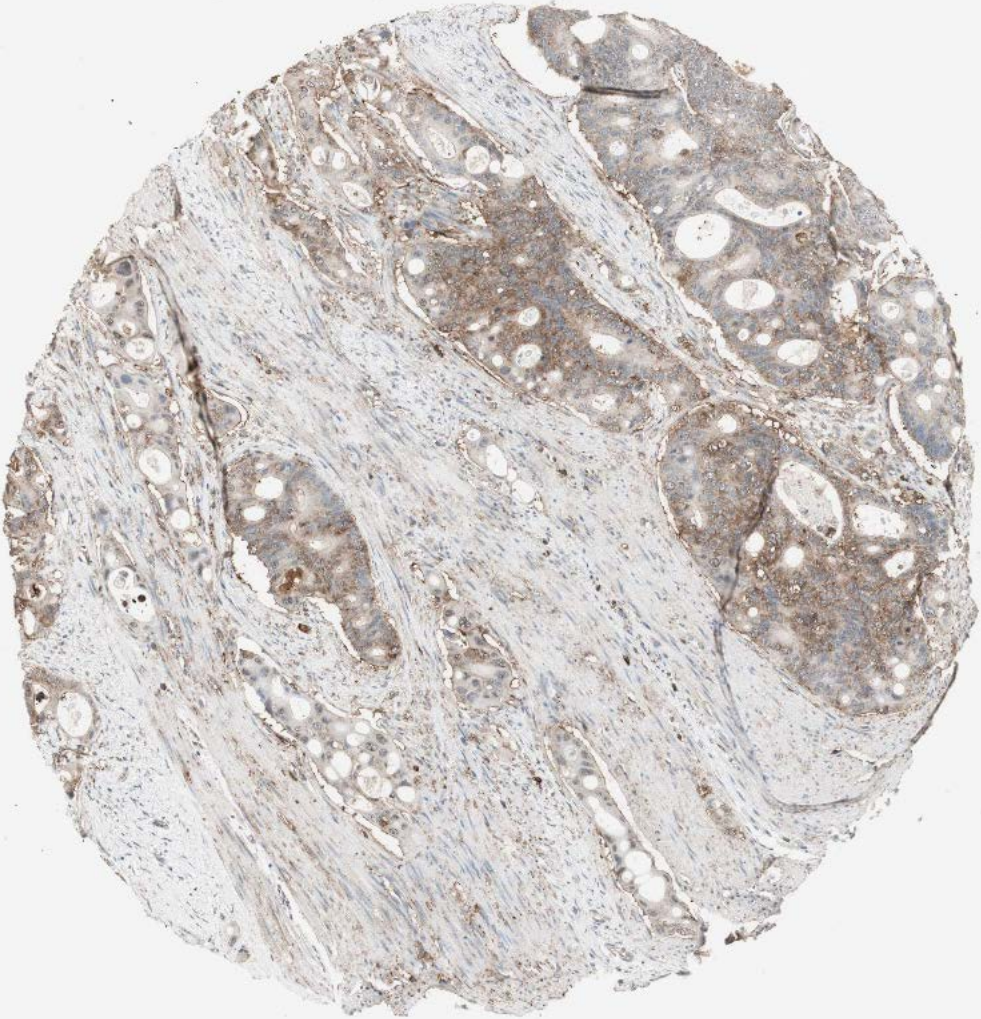

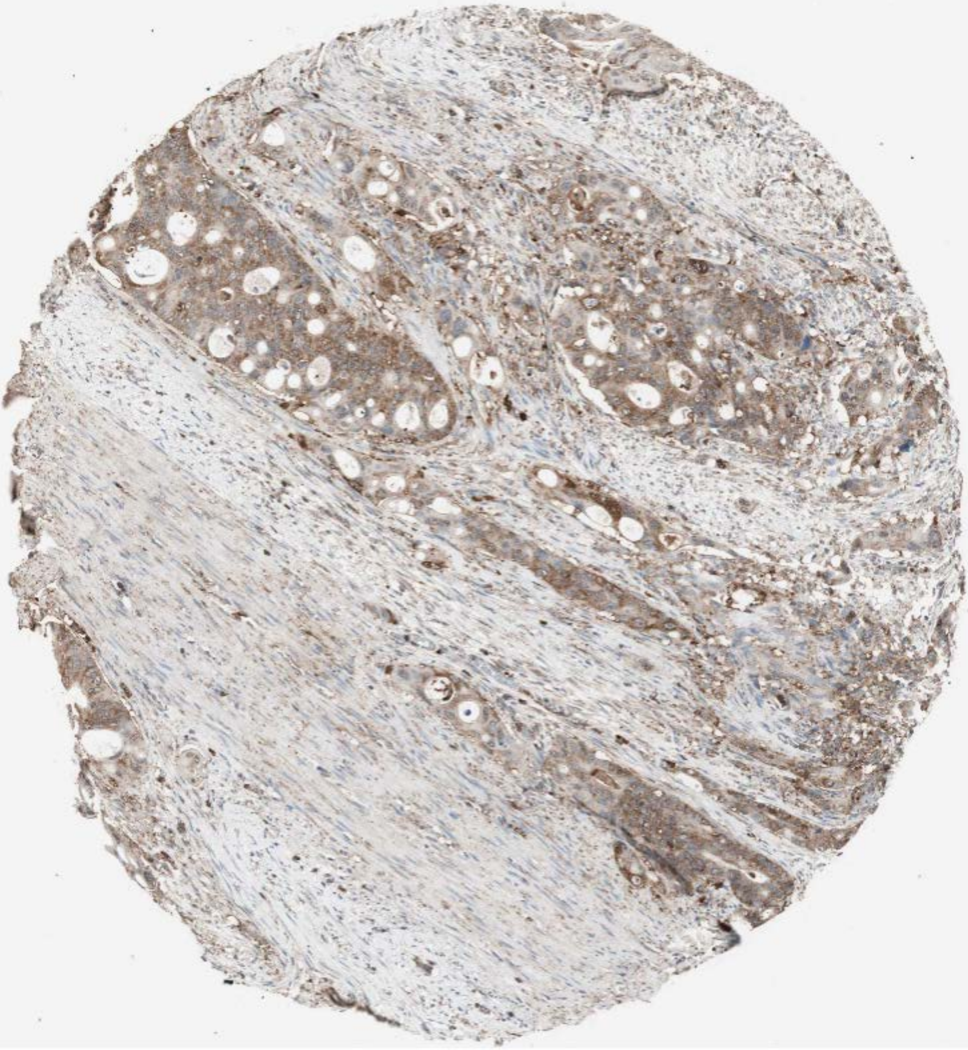

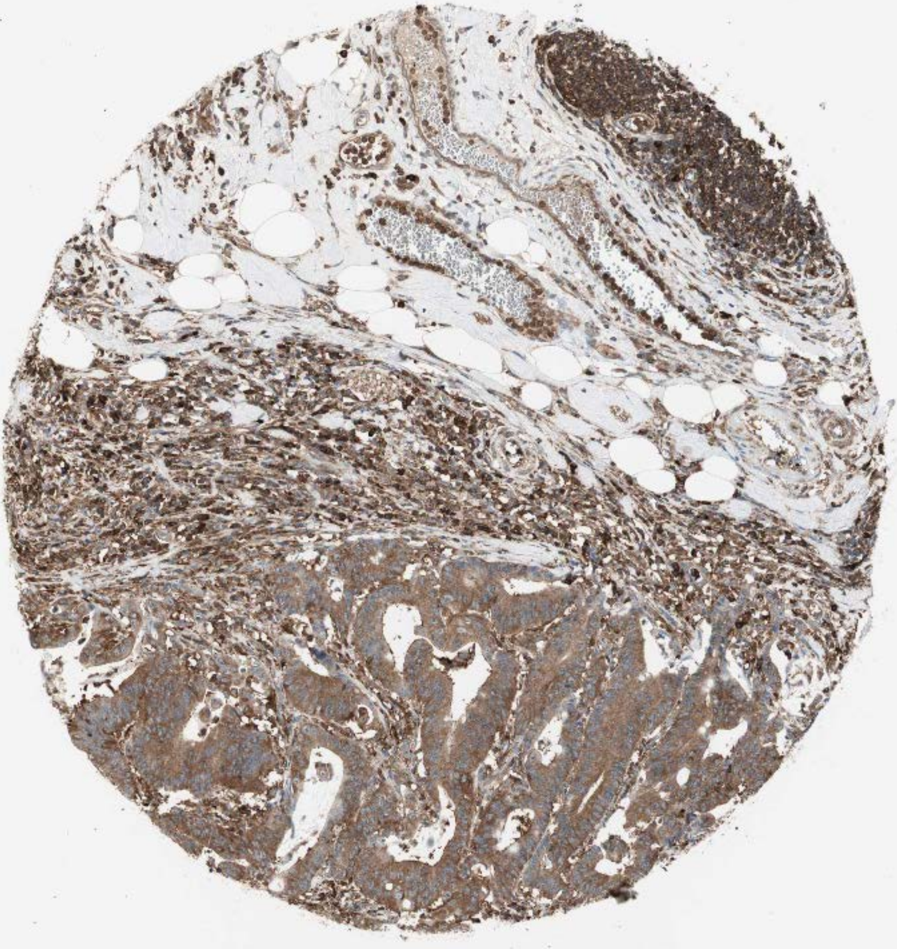

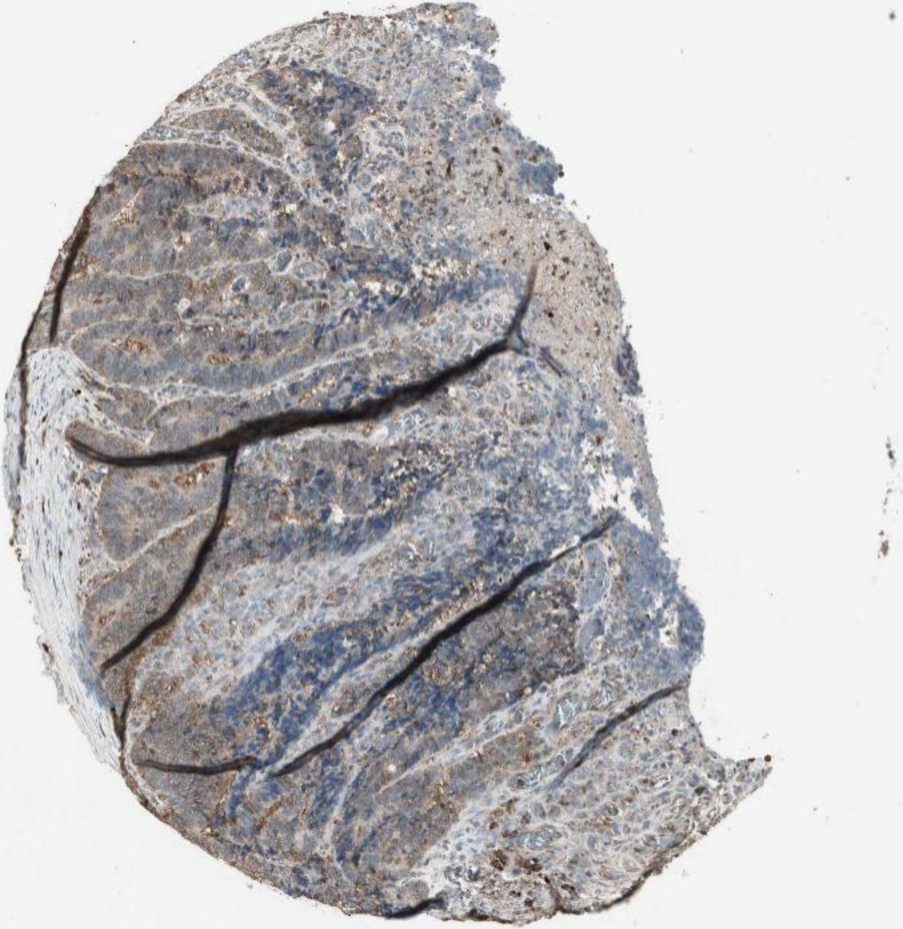

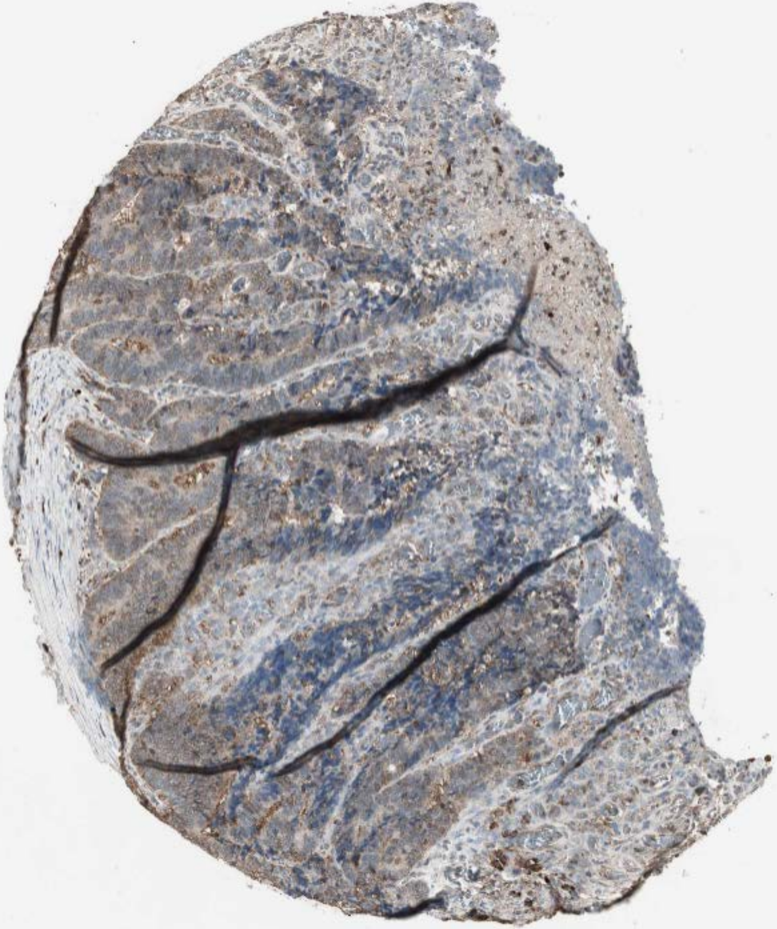

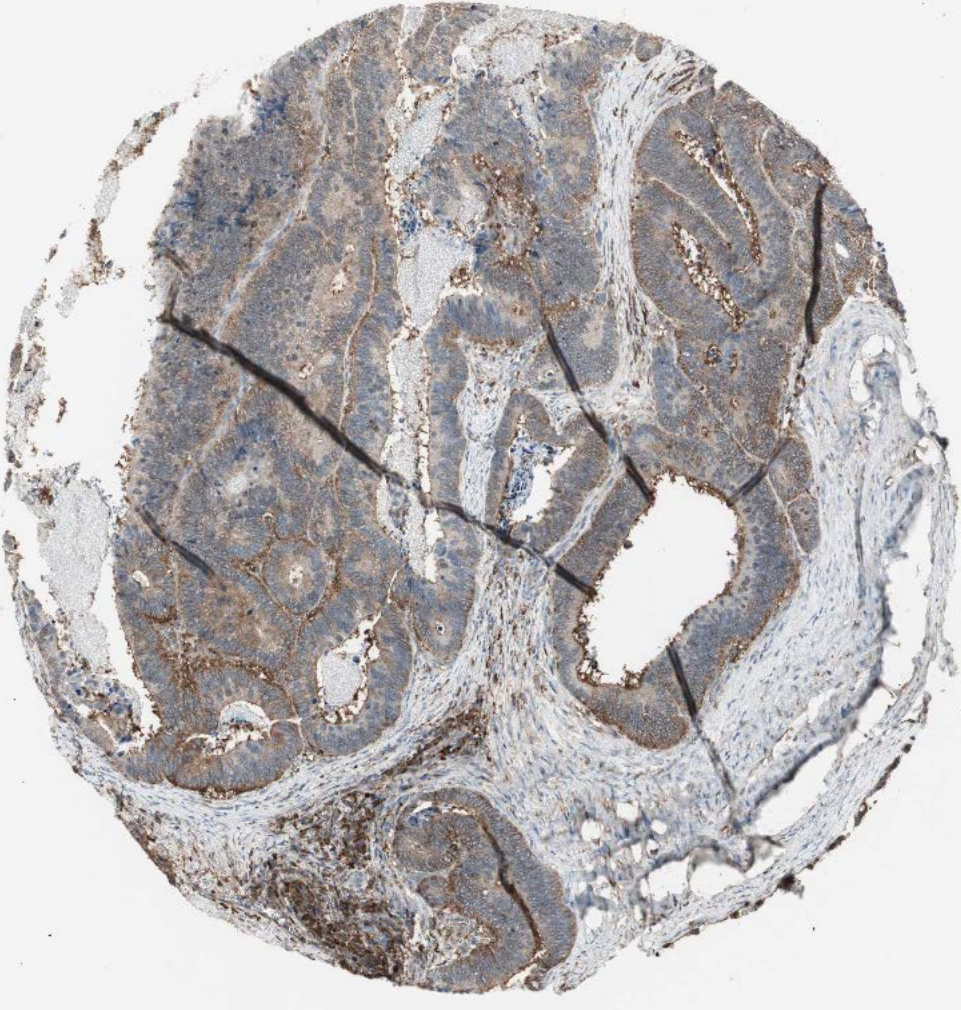

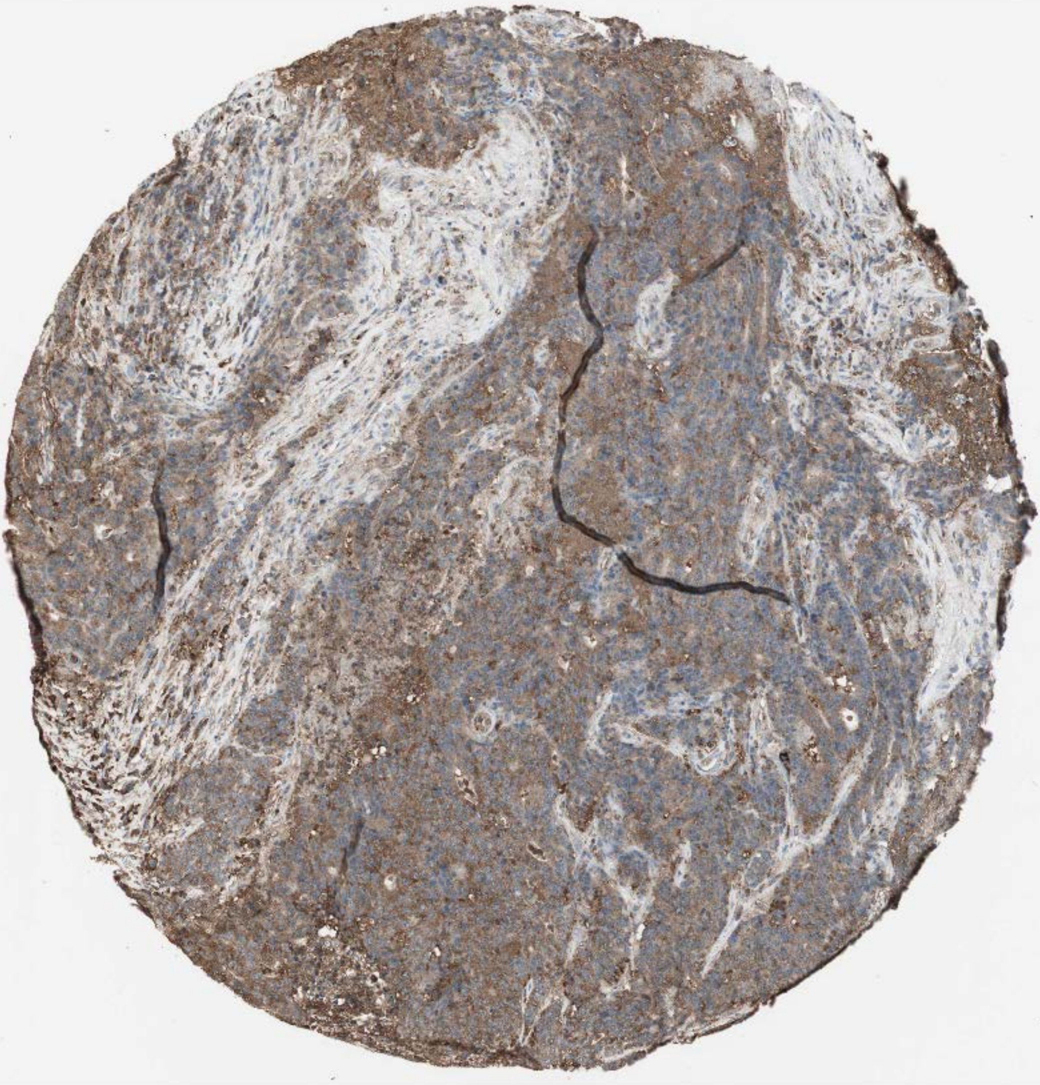

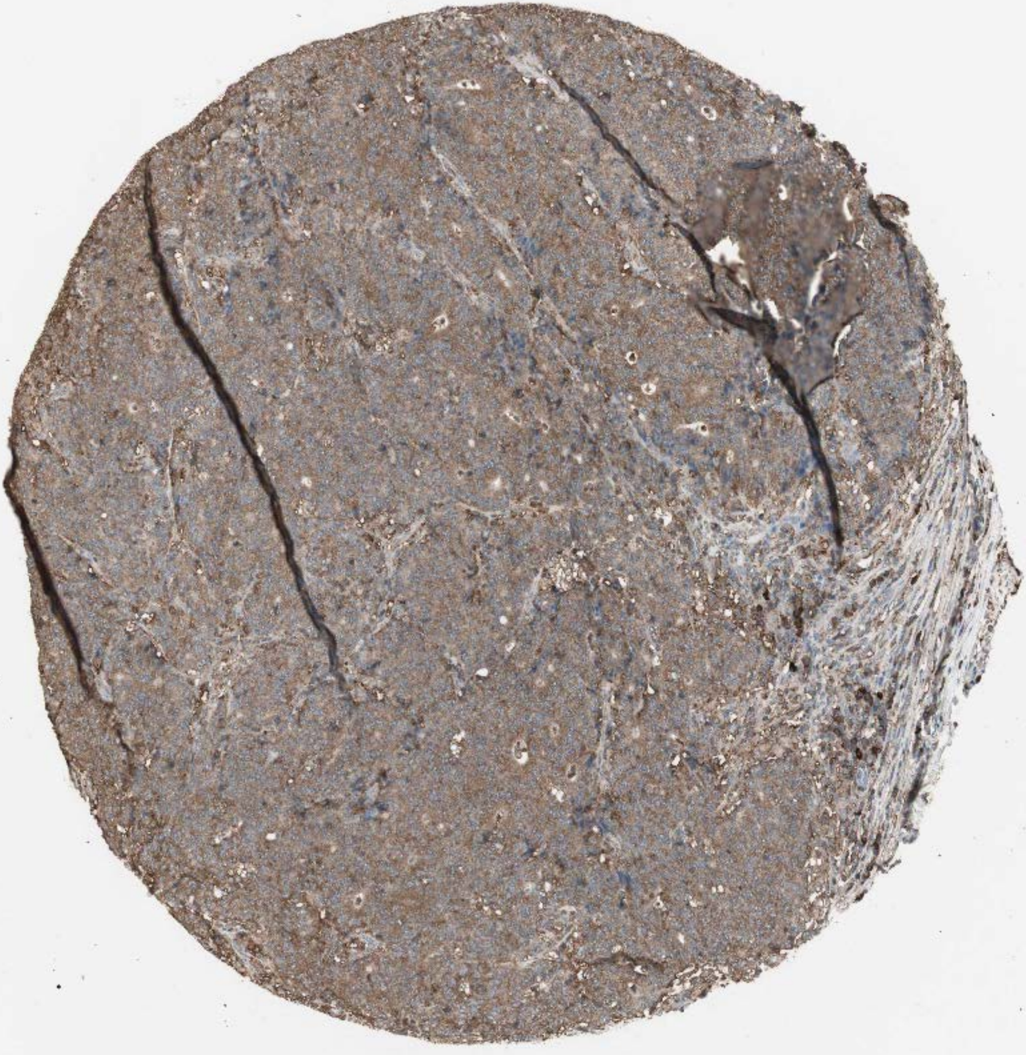

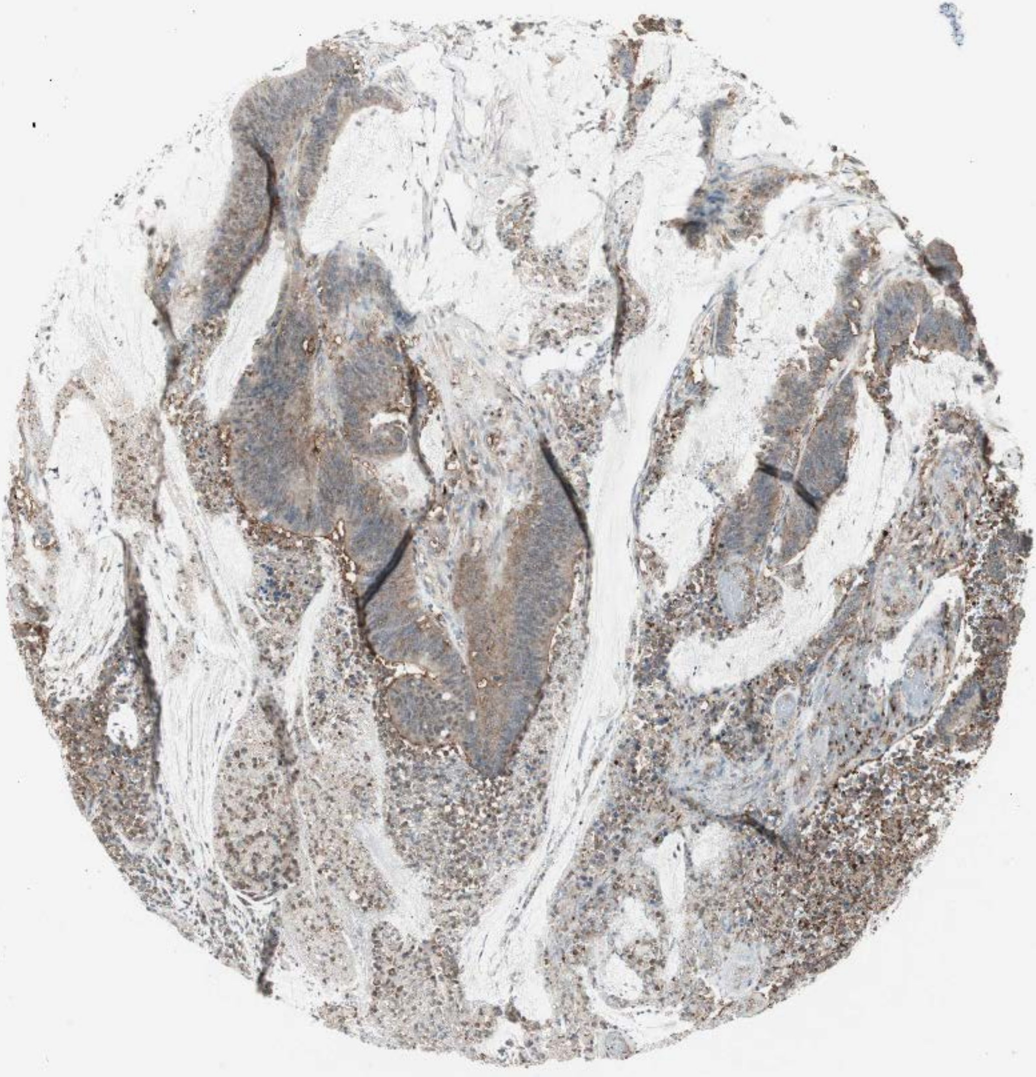

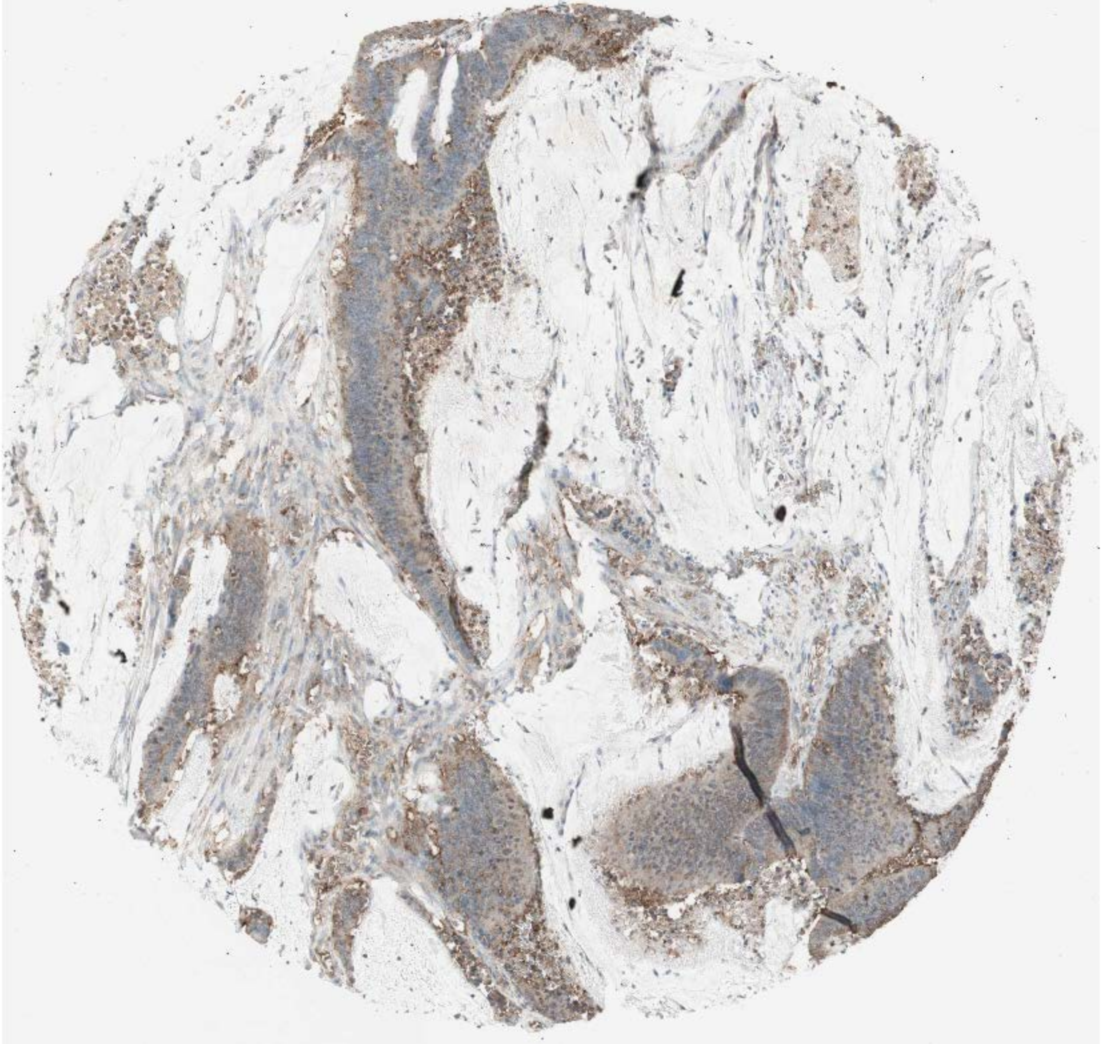

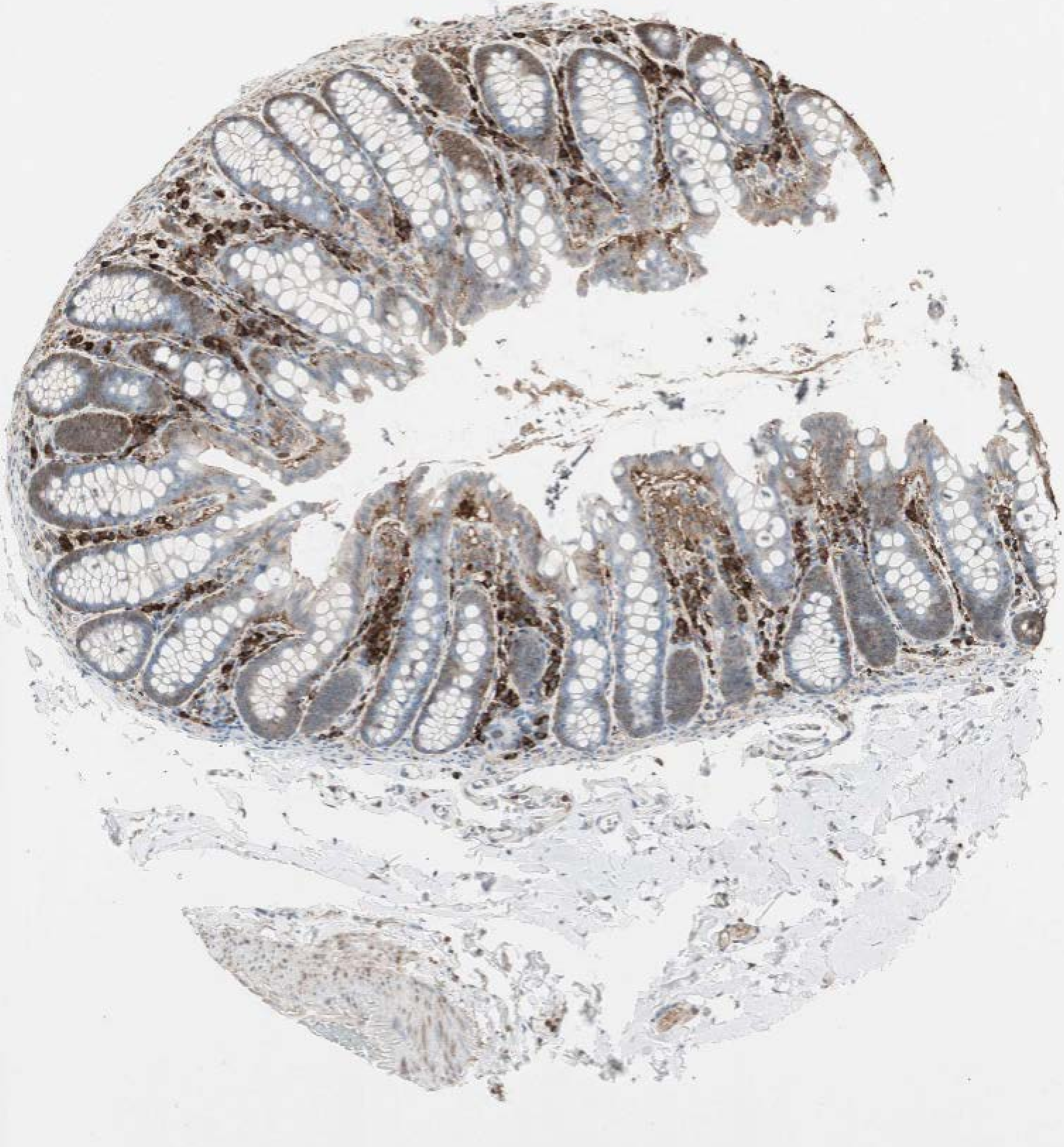

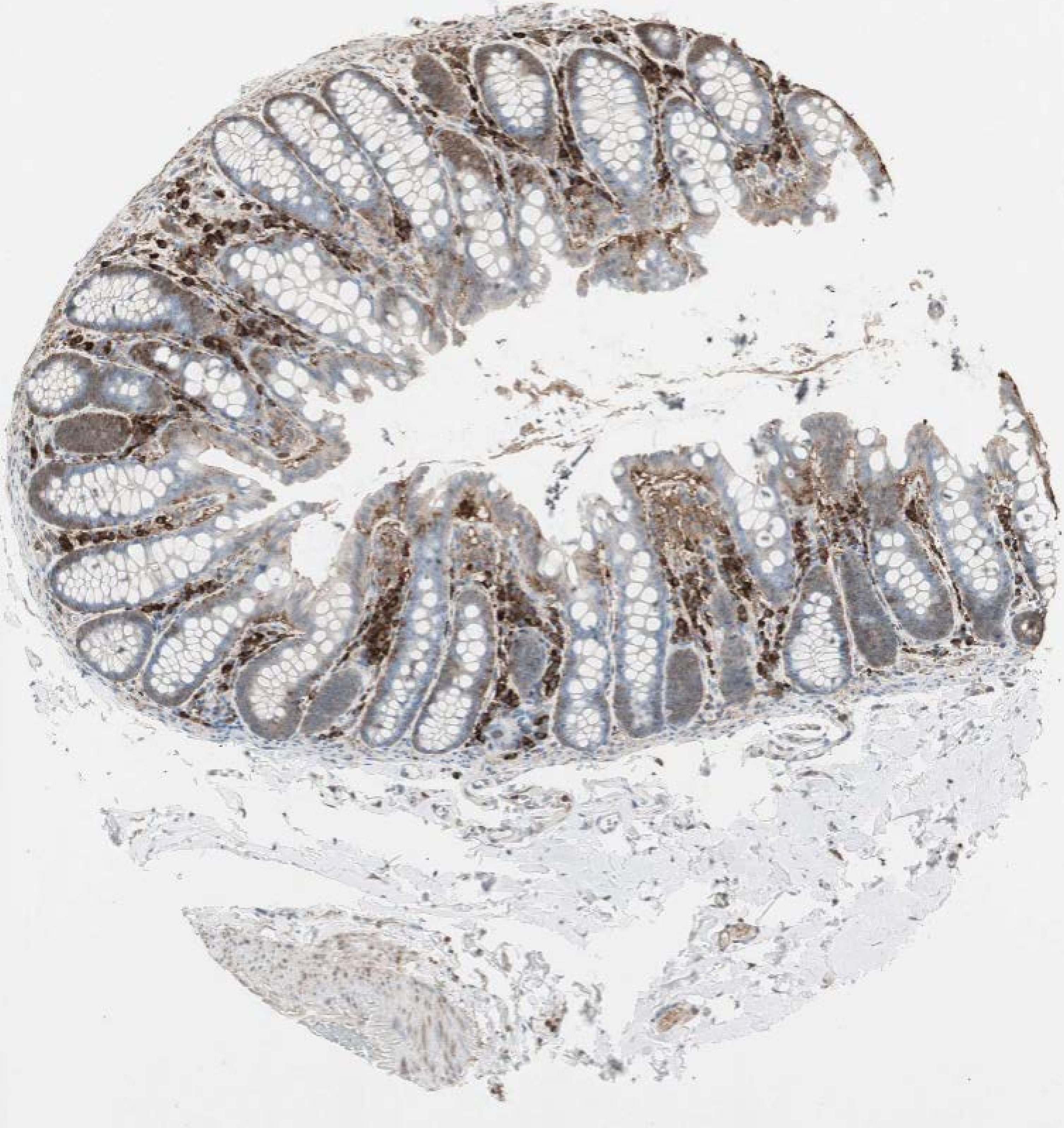

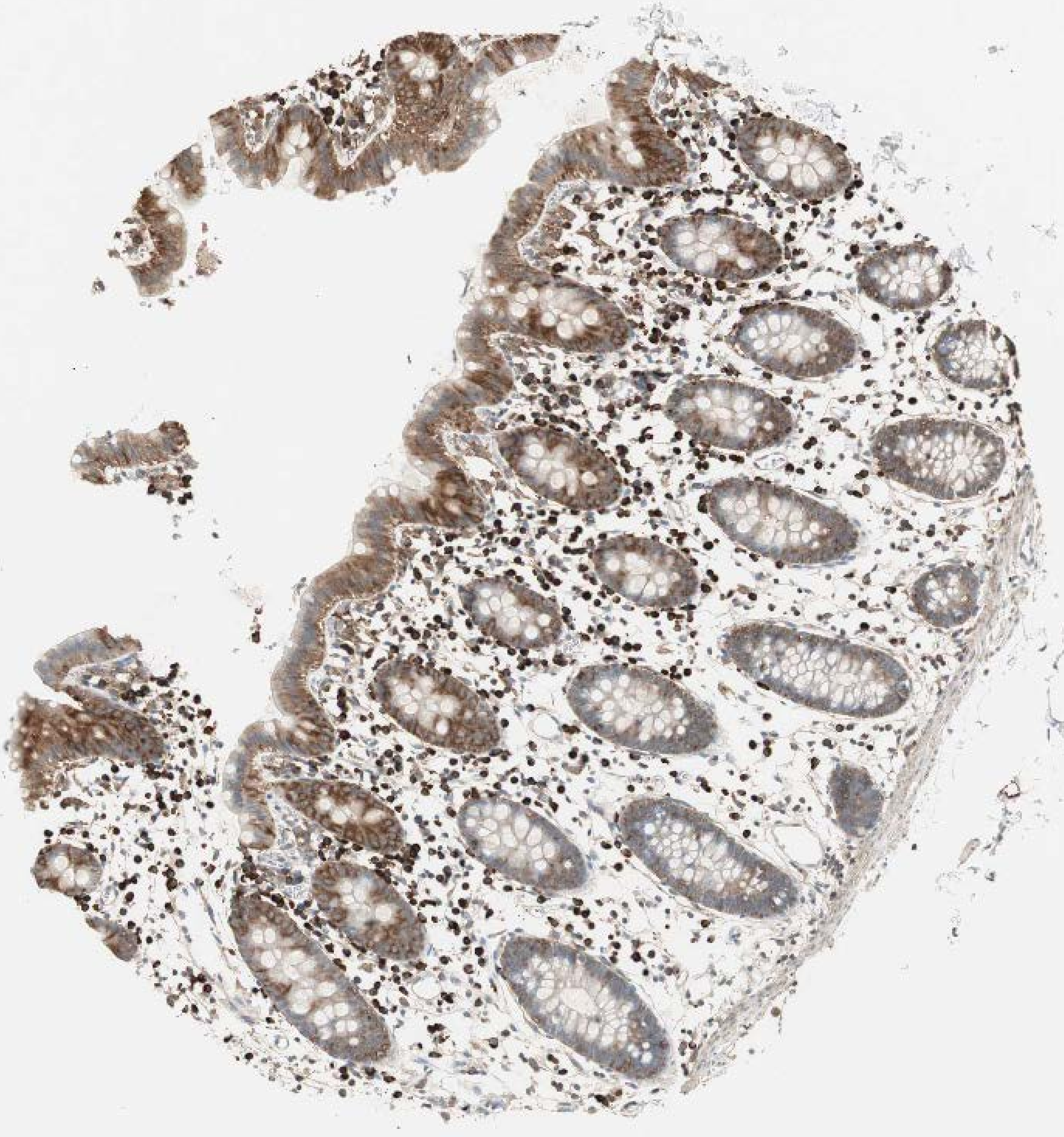

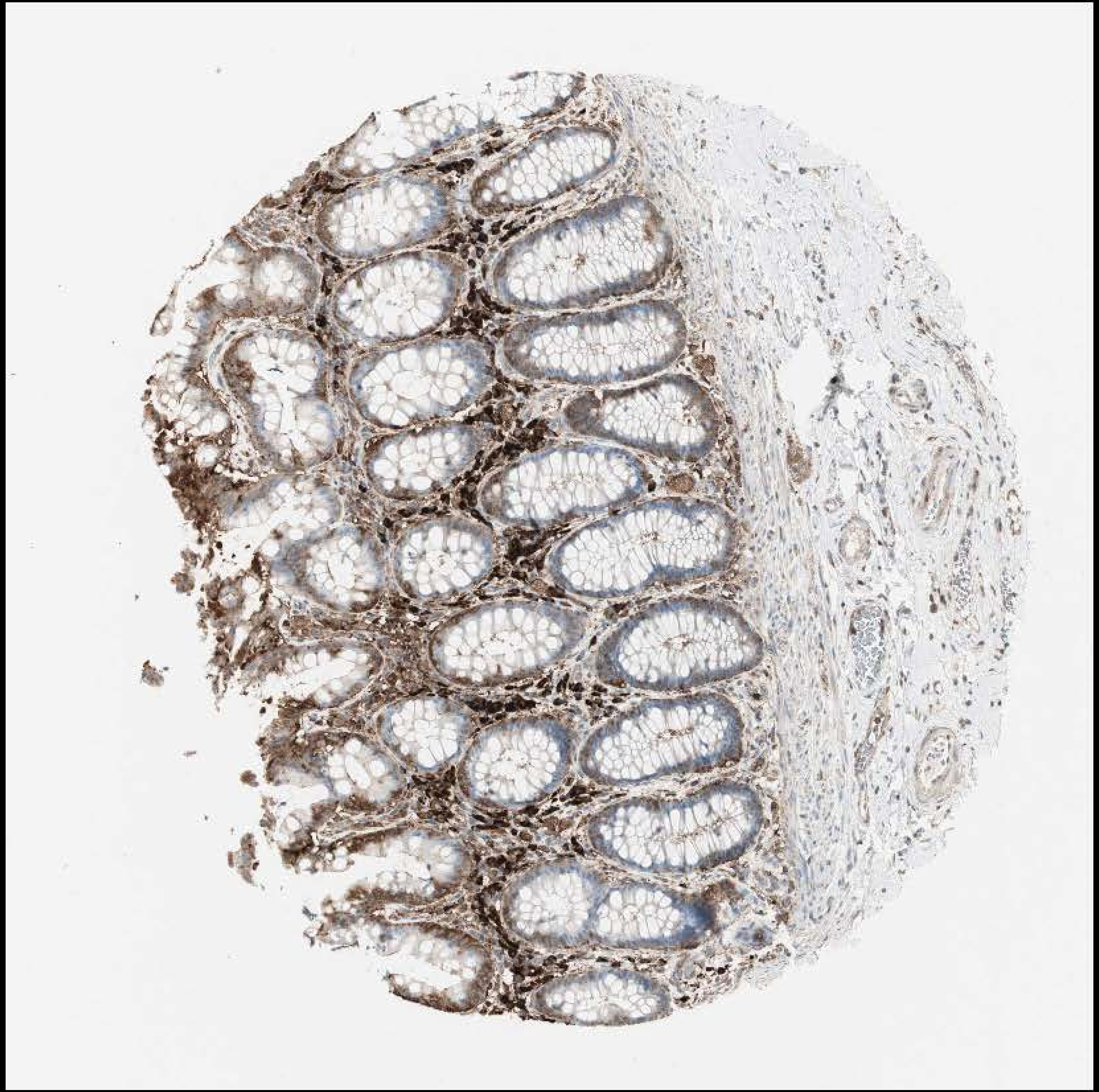

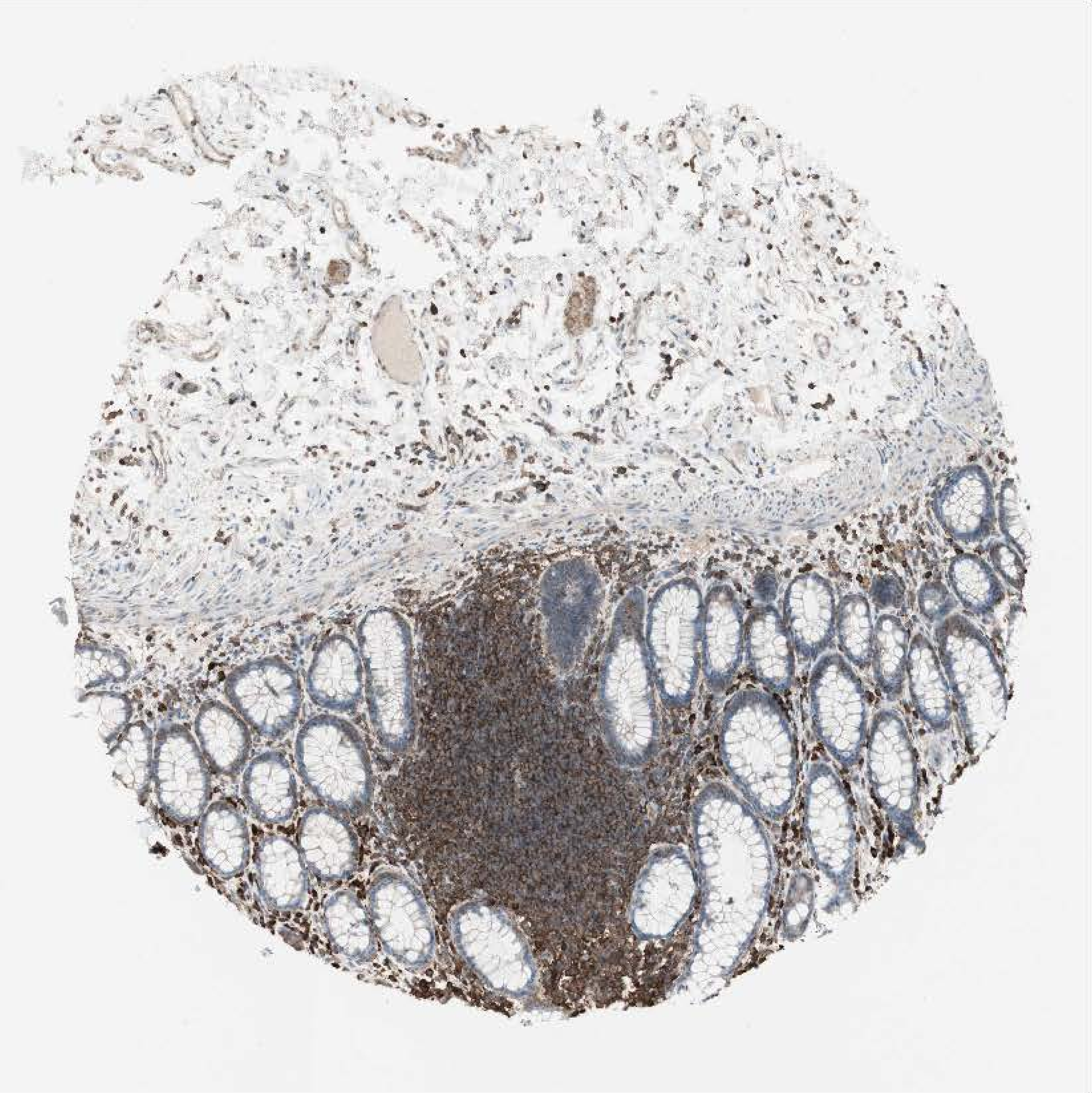

Supplement: Supplementary file 3 — Supplementary figure 3. [file jcav14p1956s3.pdf]
